# Patient-Specific iPSC-Derived Astrocytes Contribute to Non-Cell-Autonomous Neurodegeneration in Parkinson's Disease

**DOI:** 10.1016/j.stemcr.2018.12.011

**Published:** 2019-01-10

**Authors:** Angelique di Domenico, Giulia Carola, Carles Calatayud, Meritxell Pons-Espinal, Juan Pablo Muñoz, Yvonne Richaud-Patin, Irene Fernandez-Carasa, Marta Gut, Armida Faella, Janani Parameswaran, Jordi Soriano, Isidro Ferrer, Eduardo Tolosa, Antonio Zorzano, Ana Maria Cuervo, Angel Raya, Antonella Consiglio

**Affiliations:** 1 Department of Pathology and Experimental Therapeutics, Bellvitge University Hospital-IDIBELL, Hospitalet de Llobregat, Barcelona 08908, Spain; 2 Institute of Biomedicine of the University of Barcelona (IBUB), Barcelona 08028, Spain; 3 Center of Regenerative Medicine in Barcelona (CMRB), Hospital Duran i Reynals, Hospitalet de Llobregat, Barcelona 08908, Spain; 4 Institute for Research in Biomedicine (IRB), Carrer Baldiri Reixac 10, Barcelona 08028, Spain; 5 Centre for Networked Biomedical Research on Bioengineering, Biomaterials, and Nanomedicine (CIBER-BBN), Madrid 28029, Spain; 6 Centre Nacional d’Anàlisi Genòmica (CNAG-CRG), Parc Científic de Barcelona, Barcelona 08028, Spain; 7 Departament de Física de la Matèria Condensada, Universitat de Barcelona, Barcelona 08028, Spain; 8 Universitat de Barcelona Institute of Complex Systems (UBICS), Barcelona 08028, Spain; 9 Centre for Networked Biomedical Research on Neurodegenerative Diseases (CIBERNED), Madrid 28049, Spain; 10 Department of Neurology, Hospital Clínic de Barcelona, Institut d’Investigacions Biomédiques August Pi i Sunyer (IDIBAPS), University of Barcelona (UB), Barcelona 08036, Spain; 11 Albert Einstein College of Medicine, Bronx, NY 10461, USA; 12 Institució Catalana de Recerca i Estudis Avançats (ICREA), Barcelona 08010, Spain; 13 Department of Molecular and Translational Medicine, University of Brescia, Brescia 25121, Italy

**Keywords:** iPSC, Parkinson's disease, non-cell-autonomous, astrocytes, α-synuclein, LRRK2, CRISPR/Cas9, disease modeling, autophagy, neurodegeneration

## Abstract

Parkinson's disease (PD) is associated with the degeneration of ventral midbrain dopaminergic neurons (vmDAns) and the accumulation of toxic α-synuclein. A non-cell-autonomous contribution, in particular of astrocytes, during PD pathogenesis has been suggested by observational studies, but remains to be experimentally tested. Here, we generated induced pluripotent stem cell-derived astrocytes and neurons from familial mutant *LRRK2* G2019S PD patients and healthy individuals. Upon co-culture on top of PD astrocytes, control vmDAns displayed morphological signs of neurodegeneration and abnormal, astrocyte-derived α-synuclein accumulation. Conversely, control astrocytes partially prevented the appearance of disease-related phenotypes in PD vmDAns. We additionally identified dysfunctional chaperone-mediated autophagy (CMA), impaired macroautophagy, and progressive α-synuclein accumulation in PD astrocytes. Finally, chemical enhancement of CMA protected PD astrocytes and vmDAns via the clearance of α-synuclein accumulation. Our findings unveil a crucial non-cell-autonomous contribution of astrocytes during PD pathogenesis, and open the path to exploring novel therapeutic strategies aimed at blocking the pathogenic cross talk between neurons and glial cells.

## Highlights

• Astrocytes contribute to dopaminergic neurodegeneration in PD
• Non-cell-autonomous damage is triggered by impaired autophagy in PD astrocytes
• Dysfunctional PD astrocytes accumulate and transfer α-synuclein to healthy DAns
• CMA activator drug prevents α-synuclein accumulation and neurodegeneration

## Graphical Abstract

**Figure:**
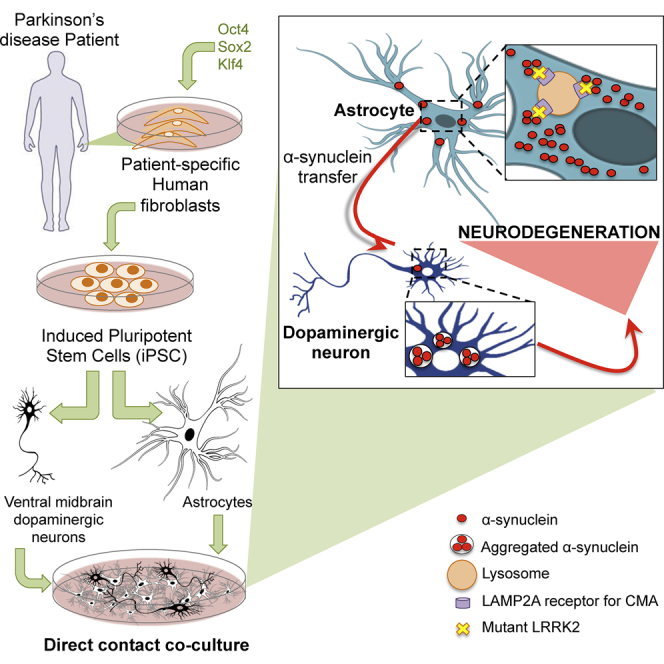

## Teaser

In this article, Consiglio and colleagues show that PD iPSC-derived astrocytes contribute to dopaminergic neurodegeneration, indicating an important role for astrocytes in the pathogenesis of Parkinson's disease. PD astrocytes display dysfunctional chaperone-mediated autophagy (CMA), impaired macroautophagy, and progressive α-synuclein accumulation. In co-culture, PD astrocytes transfer α-synuclein to vmDAns and trigger dopaminergic neuronal cell death that can be rescued by treatment with a chemical enhancement of CMA.

## Introduction

Parkinson's disease (PD) is the second most prevalent neurodegenerative disease after Alzheimer's disease, affecting 7 to 10 million people worldwide (Global Burden of Disease Study Collaborators, 2015). PD is characterized by a significant loss of ventral midbrain dopaminergic neurons (vmDAns) in the substantia nigra pars compacta. The presence of intracellular protein aggregates of α-synuclein (α-syn) in the surviving vmDAns has been reported in postmortem PD tissue (Greenamyre and Hastings, 2004). Most PD cases are sporadic (85%), but familial mutations are accountable for 15% of patients (Lill, 2016). Mutations in the gene encoding leucine-rich repeat kinase 2 (*LRRK2*), causing an autosomal dominant form of PD, account for 5% of familial cases and 2% of sporadic cases (Gilks et al., 2005, Nichols et al., 2005). LRRK2 is a highly complex protein with both GTPase and protein kinase domains involved in several cellular functions, including autophagy (Cookson, 2016, Orenstein et al., 2013, Su et al., 2015).

Correlations between mutant LRRK2 and several pathogenic mechanisms linked to PD progression have been previously reported, including alterations in autophagy and consequent accumulation of α-syn (Cookson, 2017). Neuronal mutant LRRK2 toxicity was found to depend on LRRK2 levels and α-syn accumulation as opposed to kinase activity or inclusion bodies in induced pluripotent stem cell (iPSC)-derived neurons (Skibinski et al., 2014). During PD pathogenesis, mutant LRRK2 was found to directly bind LAMP2A, the receptor responsible for chaperone-mediated autophagy (CMA) normally used by both LRRK2 and α-syn for degradation (Orenstein et al., 2013). This binding blocks the proper functioning of the CMA translocation complex, resulting in defective CMA, leading to the accumulation of α-syn and cell death.

iPSCs derived from healthy individuals and patients have accelerated advances in developing genuinely human experimental models of diseases (Zeltner and Studer, 2015). In the case of PD, previous studies by our groups and others have generated iPSCs from patients with PD associated with *LRRK2* mutations, and described the appearance of disease-specific phenotypes in iPSC-derived neurons, including impaired axonal outgrowth and deficient autophagic vacuole clearance (Heman-Ackah et al., 2017, Nguyen et al., 2011, Sanchez-Danes et al., 2012, Skibinski et al., 2014). Moreover, dopaminergic (DA) neurons from *LRRK2*-mutant iPSCs displayed alterations in CMA that were, at least in part, responsible for the abnormal accumulation of α-syn observed in these cells, which predated any morphological signs of neurodegeneration (Orenstein et al., 2013).

Studies investigating PD pathogenesis have been mostly focused on the mechanisms underlying vmDAn degeneration and death. However, there is evidence of astrocytes accumulating α-syn during PD through postmortem analysis (Braak et al., 2007, Wakabayashi et al., 2000). Altered α-syn released by axon terminals in the surrounding synapses is taken up by astrocytes, supporting the hypothesis of the spread of α-syn through neuron-astrocyte interactions (Braak et al., 2007, Lee et al., 2010). Overexpression of mutant *SNCA* in primary astrocytes altered their normal functioning and impaired proper blood-brain barrier control and glutamate homeostasis, and eventually resulted in a significant loss of vmDAns (Gu et al., 2010). In a study using human brain homogenates from PD patients with Lewy bodies, α-syn was found to be taken up and spread from astrocytes to neurons, leading to neuronal death (Cavaliere et al., 2017). As a result, a role of astrocyte dysfunction in PD pathogenesis is emerging (Booth et al., 2017).

In the present study, we generated patient-specific iPSC-derived astrocytes and vmDAns from PD patients with the *LRRK2* G2019S mutation and healthy individuals. We consistently generated a population of human vmDAns *in vitro* that expressed postmitotic dopaminergic markers and fired action potentials. Subsequently, we co-cultured healthy iPSC-derived vmDAns with iPSC-derived astrocytes expressing the mutated form of *LRRK2* associated with PD. In co-culture experiments, we detected a significant decrease in the number of vmDAns in the presence of *LRRK2*-PD astrocytes, which correlated with the abnormal accumulation of astrocyte-derived α-syn. Conversely, control astrocytes were able to partially rescue disease-related phenotypes in *LRRK2*-PD vmDAns during co-culture. A more in-depth investigation revealed impaired autophagic machinery, as well as progressive accumulation of endogenous α-syn in PD astrocytes, compared with control astrocytes. By treating the cells with an activator of CMA, we were able to prevent the appearance of PD-related phenotypes in patients' astrocytes. Overall, our findings represent a direct indication that dysfunctional astrocytes play a crucial role during PD pathogenesis and may have broad implications for future intervention in early stages of PD.

## Results

### Generation and Characterization of iPSC-Derived Patient-Specific Astrocytes

**Figure 1:**
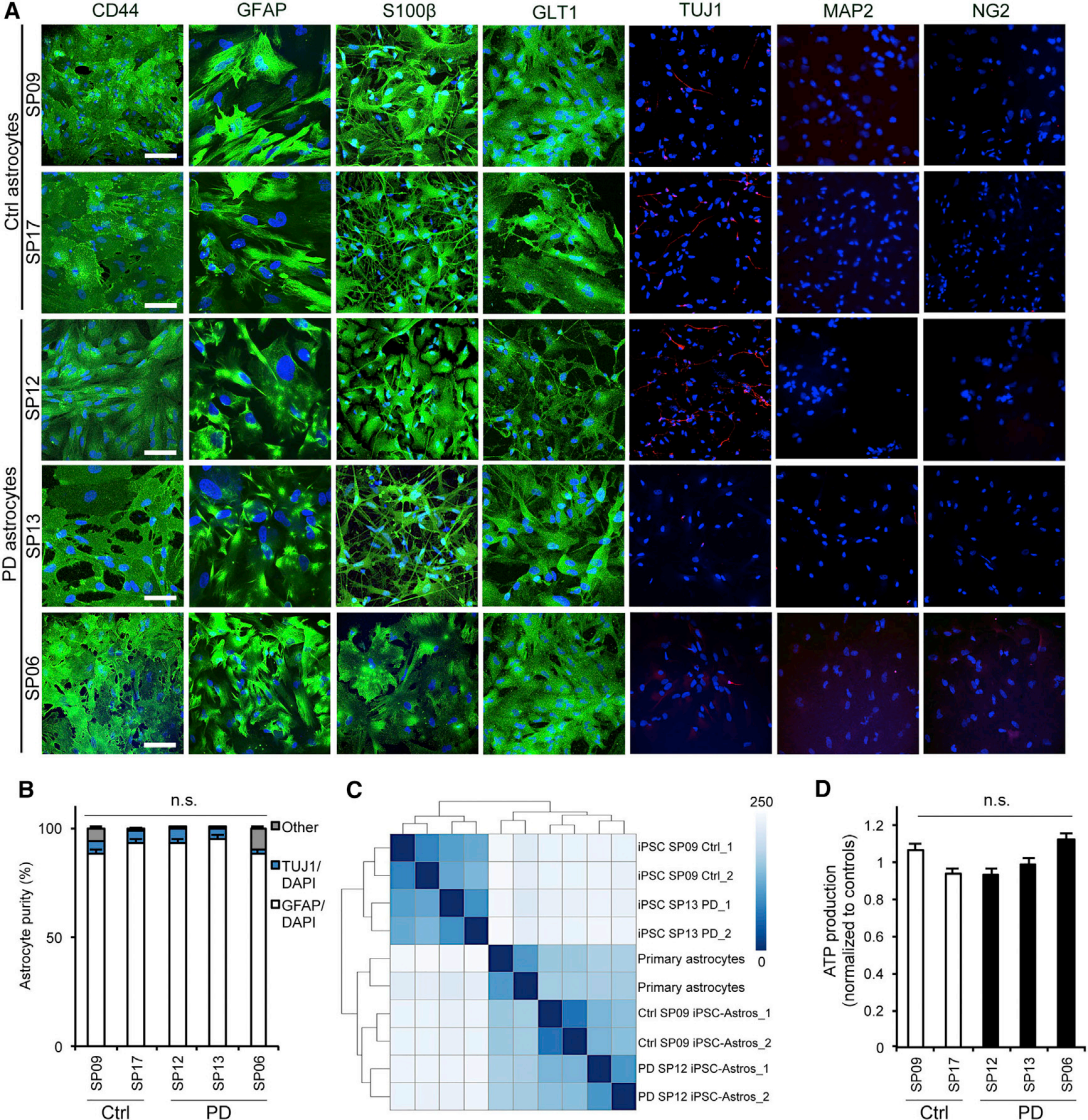
iPSC-Derived Patient-Specific Astrocyte Generation and Characterization (A) Representative ICC images of astrocytes from two Ctrl iPSC lines (Ctrl SP09 and Ctrl SP17) and three PD iPSC lines (PD SP12, PD SP13, and PD SP06) staining positive for CD44 (astrocytic precursor marker), GFAP (general astrocytes), S100β (mature astrocytes), and GLT1 (excitatory amino acid transporter 2), and negative for TUJ1 (immature neurons), MAP2 (mature neurons), and NG2 (oligodendrocytes) expression. Number of independent astrocyte lines generated from iPSC per patient = 3. Number of independent experiments per astrocyte line generated = 3. Scale bar, 100 μm. (B) Astrocyte cultures are composed of approximately 95% astrocytes, 4% neurons, and 1% other (n = 3). (C) Heatmap showing sample similarities taking the rlog transformed data and Euclidean distances between samples. iPSC-derived astrocyte (Ctrl SP09 and PD SP12) samples cluster closer to the human primary astrocytes than the corresponding iPSC group (n = 2). (D) Functional ATP production luminescence (counts normalized to controls) in both Ctrl (SP09 and SP17) and PD (SP13, SP12, and SP06) astrocytes (n = 3). Data are expressed as mean ± SEM, unpaired two-tailed Student's t test.

To establish an *in vitro* human cellular model for dissecting the interplay between neurons and astrocytes in PD, we first derived astrocyte-like cells from iPSCs, using a previously published protocol (Serio et al., 2013). Specifically, astrocyte cultures were successfully established from iPSC lines from three PD patients carrying the G2019S mutation in the *LRRK2* gene (PD SP06, PD SP12, and PD SP13) and two healthy age-matched controls (Ctrl SP09 and Ctrl SP17) (see Table 1 and Tables S1 and S2 for a summary of the iPSC lines used, and Experimental Procedures for details on their origin). Immunocytochemistry (ICC) detection of key astrocyte markers showed robust expression of CD44, glial fibrillary acidic protein (GFAP), and S100 calcium-binding protein β (S100β), as well as of the excitatory amino acid transporter 2 (EAAT2, also known as GLT1), in all human iPSC-derived astrocytes (Figure 1A). No evident contamination by other cell types, such as neurons or oligodendroglial progenitors, was found as assessed by immunostaining with anti-MAP2 or NG2 antibody, respectively (Figures 1A and 1B). The astrocytic identity was further confirmed by quantitative RT-PCR of *GFAP* and additional astrocyte-specific genes, including *MLC1*, *SOX9*, *ALDH1L1*, *AQP4*, *DIO2*, and *SLC4A4*, which were expressed in Ctrl and PD astrocytes, and in human primary astrocytes, but not in iPSCs (Figures S1A–S1C).

**Table 1:** Summary of the Healthy Controls and Patients Used in This Study

Code	Status	Sex	Age at Biopsy	Mutation	Isogenic Control
SP09	control	M	66	no	
SP11	control	M	52	no	
SP17	control	F	48	no	
SP06	Parkinson’s disease	M	44	*LRRK2* G2019S	
SP12	Parkinson’s disease	F	63	*LRRK2* G2019S	
SP13	Parkinson’s disease	F	68	*LRRK2* G2019S	*LRRK2* G2019S corrected

To validate astrocyte cell type identity, we also tested the expression of astrocyte-specific genes revealed by the Human Astrocyte RNA-Seq database (www.brainrnaseq.org/) in our iPSC-derived astrocytes, through RNA sequencing. We found that the transcriptomic profile of both Ctrl and PD iPSC-derived astrocytes was closer to that of human primary astrocytes than to that of their corresponding iPSC line, thus confirming their identity (Figure 1C). We next determined the functional maturation of iPSC-derived astrocytes by confirming their capacity to produce ATP (Figure 1D) and propagate intercellular Ca^2+^ waves (Figures S1D–S1H). Indeed, by using the Fluo-4 AM Ca^2+^ indicator, recordings from Ctrl and PD astrocytes showed a heterogeneous pattern of Ca^2+^ fluctuations under basal conditions, revealing their functionality. All together, these data support the successful generation of highly pure populations of functionally equivalent astrocyte-like cells, which represent a continuous source of human Ctrl and PD astrocytes for subsequent analyses.

### Generation of vmDAns and Setup of Neuron-Astrocyte Co-culture System

**Figure 2:**
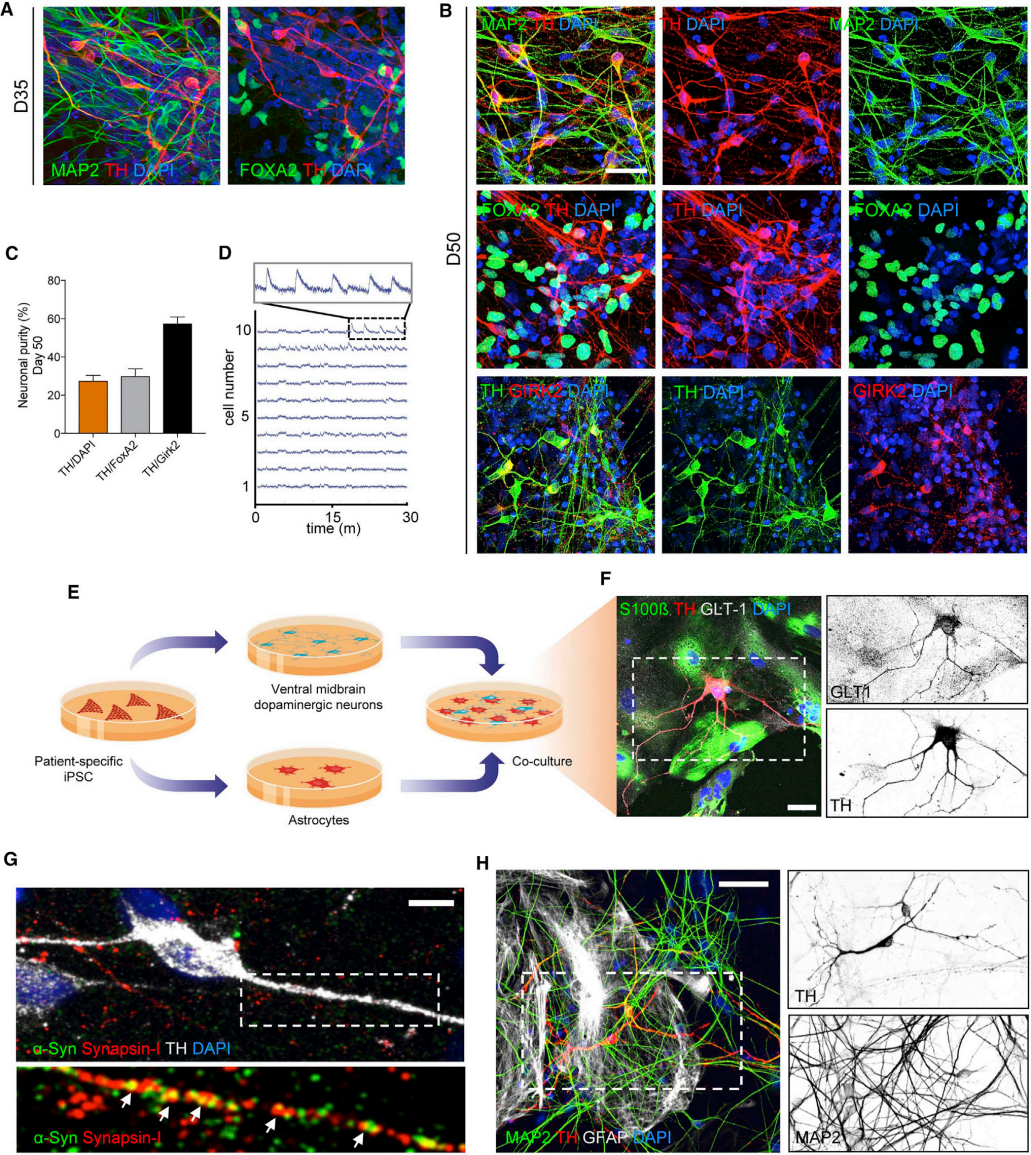
vmDAn Generation, Characterization, and Co-culture Setup (A and B) Representative immunofluorescence images of Ctrl SP11 vmDAn after (A) 35 or (B) 50 days of neuronal differentiation. iPSC-derived neural cultures express markers specific for neurons (MAP2), DAns (TH), and midbrain-type DAns (FOXA2 and GIRK2). Scale bar, 20 μm. (C) Percentage of differentiated cells that stained positive for TH and double positive for TH and FOXA2 and TH and GIRK2 after 50 days of differentiation (n = 4). (D) Calcium wave flux recording over 30 min with calcium tracer Fluo-8 AM of vmDAns at day 50 (n = 3). (E) Diagram of co-culture system. (F) Representative ICC images of 4-week co-culture staining positive for Ctrl SP11 vmDAns (TH), Ctrl SP17 astrocytes (S100β), excitatory amino acid transporter 2 (GLT1), and nuclear DAPI. Scale bar, 20 μm. (G) Representative ICC images of presynaptic markers α-syn and synapsin-1 of a Ctrl SP11 vmDAn (TH) on the top of Ctrl SP11 astrocytes after 4 weeks in co-culture. Scale bar, 10 μm. (H) Representative ICC images of Ctrl SP11 vmDAns (TH) and mature neurons (MAP2) on the top of Ctrl SP09 astrocytes (GFAP) during a 4-week co-culture period. Scale bar, 20 μm. Boxed area on the left in (F), (G), and (H) is shown on the right.

To investigate whether astrocytes contribute to PD pathogenesis, we established a co-culture system of iPSC-derived astrocytes and iPSC-derived vmDAns. PD vmDAns were generated from iPSC lines from two PD patients carrying the G2019S mutation in the *LRRK2* gene (iPSC lines PD SP12 and PD SP13), whereas Ctrl vmDAns were obtained from two independent iPSC clones (Ctrl SP11 and Ctrl SP11#4) from a healthy age-matched control (see Tables 1, S1, and S2 for a summary of the iPSC lines used). To differentiate iPSCs toward vmDAns, we used a combination of two previously published (Chambers et al., 2009, Kriks et al., 2011) midbrain floor-plate differentiation protocols that was comparably effective in all iPSC lines analyzed. Under these conditions, ∼20% of cells in the cultures were committed to DA neuron fate by day 35 of differentiation, as judged by the expression of tyrosine hydroxylase (TH) and forkhead box protein A2 (FOXA2) (Figure 2A). By day 50 of differentiation, the percentage of TH^+^ neurons reached ∼30%, most of which also expressed the A9-domain-specific marker G-protein-activated inward rectifier potassium channel 2 (GIRK2), and displayed spontaneous action potential firing (Figures S2B–S2D). For co-culture experiments with control astrocytes, we dissociated vmDAn cultures after 35 days of differentiation and plated them onto a confluent layer of Ctrl iPSC-derived astrocytes (Figure 2E). After 4 weeks of co-culture, we found that astrocyte-neuron glutamate exchange was present through glutamate transporter 1 (GLT1) expression (Figure 2F) and neuronal synapse formation (Figure 2G). Accordingly, an overall healthy neuronal network comprising MAP2-positive cells was formed upon co-culture (Figure 2H).

### Control vmDAns Show Morphological Signs of Neurodegeneration when Co-cultured with PD Astrocytes

**Figure 3:**
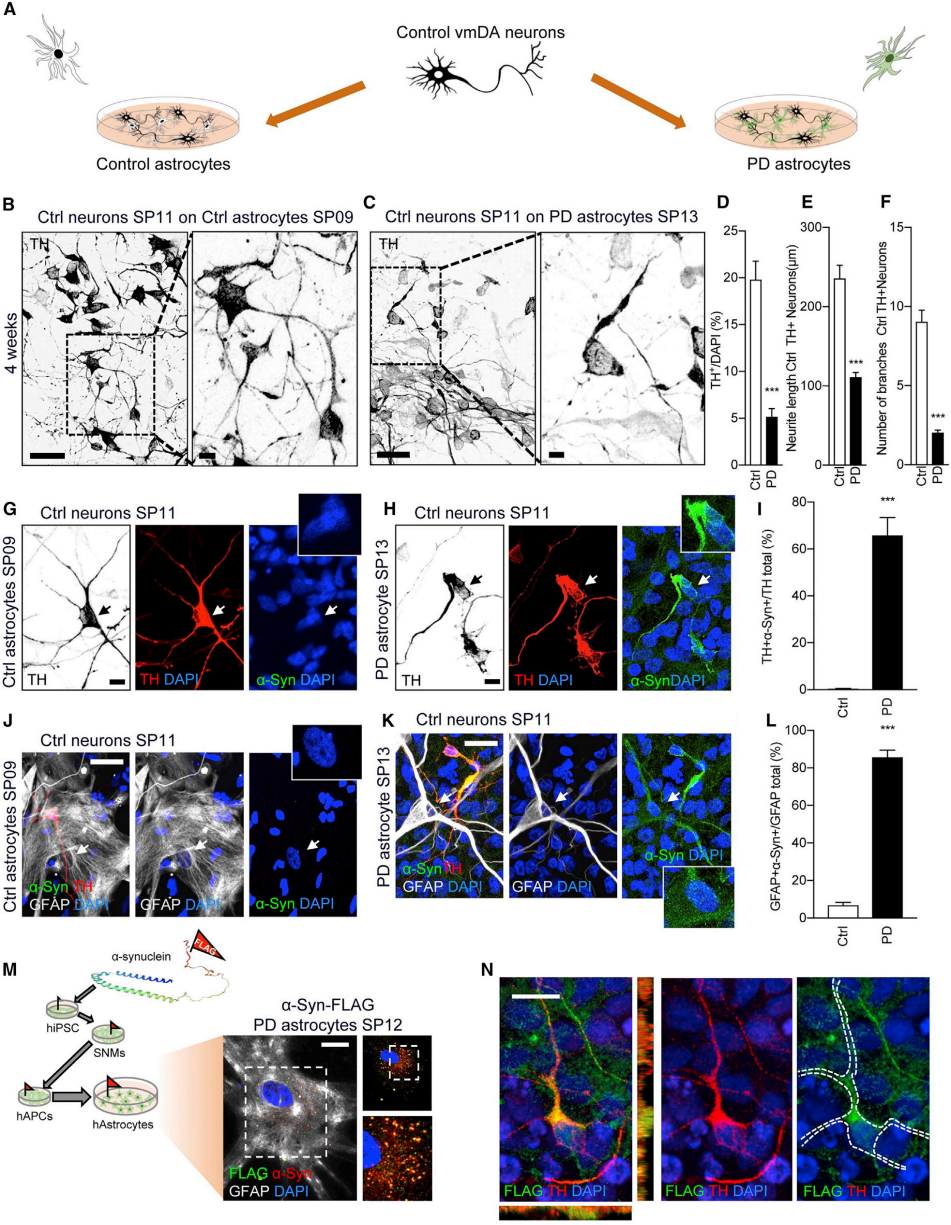
Ctrl Neurons Show Signs of Neurodegeneration and Accumulate α-syn when Co-cultured with PD Astrocytes (A) Scheme representing co-culture system of Ctrl neurons on the top of Ctrl or PD astrocytes for 4 weeks. (B and C) Representative ICC images of tyrosine hydroxylase (TH, black) from co-cultures of Ctrl SP11 neurons with (B) Ctrl SP09 astrocytes and (C) PD SP13 astrocytes for 4 weeks. Images on the right show a magnification of the area boxed in the left images; scale bars, (left) 20 μm and (right) 0.2 μm. (D) Percentage of TH^+^/DAPI of Ctrl SP11 and Ctrl SP11#4 neurons when co-cultured with Ctrl SP09, Ctrl SP17, or PD SP12, PD SP13, and PD SP06 astrocytes for 4 weeks (n = 3 per combination). (E and F) (E) Neurite length quantification and (F) number of branches of Ctrl SP11 TH-positive neurons and Ctrl SP11#4 TH-positive neurons when co-cultured on Ctrl SP09, Ctrl SP17, and Ctrl SP11 astrocytes or PD SP12, PD SP13, and PD SP06 astrocytes for 4 weeks (n = 3); 30 neurons counted per experiment. (G and H) Representative ICC images of Ctrl SP11 vmDAns co-cultured with (G) Ctrl SP09 and (H) PD SP13 astrocytes after 4 weeks and stained for TH (vmDAn), α-syn, and DAPI. Arrows indicate the selected cell for which an insert is shown at higher magnification. Scale bar, 0.2 μm. (I) Quantitative analysis of the percentage of vmDAns stained positive for α-syn when Ctrl SP11 and Ctrl SP11#4 neurons were co-cultured with Ctrl SP09, Ctrl SP17, or PD SP12, PD SP13, and PD SP06 astrocytes for 4 weeks (n = 3). (J and K) Representative ICC images of (J) Ctrl SP09 or (K) PD SP13 astrocytes co-cultured with Ctrl SP11 vmDAns for 4 weeks, stained for TH (vmDAn), GFAP (astrocytes), α-syn, and DAPI. Arrows indicate the selected cell for which an insert is shown at higher magnification. Scale bar, 20 μm. (L) Quantitative analysis of the percentage of astrocytes stained positive for α-syn when Ctrl SP11 and Ctrl SP11#4 neurons were co-cultured with Ctrl SP09, Ctrl SP17, or PD SP12, PD SP13, and PD SP06 astrocytes for 4 weeks (n = 3). (M) Scheme representing the generation of CRISPR/Cas9 edited α-syn-FLAG astrocyte line. Representative image of α-syn-FLAG PD SP12 astrocyte (GFAP) showing perfect α-syn (red) and FLAG (green) co-localization. Scale bar, 20 μm. (N) Representative ICC image depicting astrocyte-derived FLAG (green) inside of a TH-positive Ctrl SP11 neuron (red) during a 4-week co-culture period with PD SP12 α-syn-FLAG astrocytes (n = 3). Dashed line shows the outline of the cell. Scale bar, 10 μm. Data are expressed as mean ± SEM, unpaired two-tailed Student's t test, ^∗∗∗^p < 0.001.

We then examined the effects of astrocytes expressing mutated *LRRK2* on the survival of Ctrl iPSC-derived vmDAns upon co-culture (Figure 3A). After 2 weeks of co-culture with PD astrocytes, Ctrl vmDAns displayed morphological alterations, including shortened neurites, and significantly decreased cell survival compared with co-cultures with Ctrl astrocytes (Figures S2A–S2C). These alterations were much more evident after 4 weeks of co-culture, when Ctrl vmDAns cultured on top of PD astrocytes showed extensive signs of neurodegenerative phenotypes (fewer and shorter neurites, and abundance of beaded-necklace neurites) and severely compromised cell survival (less than ∼25% of control), compared with co-cultures with control astrocytes (Figures 3B–3F). The fact that the numbers of vmDAns did not change significantly during the co-culture with control astrocytes, but progressively declined when co-cultured with PD astrocytes, strongly suggests that vmDAns were lost under the latter conditions as a result of neurodegeneration, rather than a blockade in vmDAn differentiation or maturation.

Viability tests of both Ctrl and PD astrocytes at 2 and 4 weeks of co-culture revealed highly similar values, indicating that neurodegenerative signs displayed by Ctrl vmDAns were not caused by a dying PD astrocyte (Figure S2D). Interestingly, vmDAn neurodegeneration upon co-culture with PD astrocytes was specific to this type of neuron, because non-dopaminergic neurons (TH^−^/MAP2^+^) did not significantly change in numbers or morphology after co-culture with Ctrl or PD astrocytes (Figures S2E–S1J). All together, these results indicate a neurotoxic capacity of PD astrocytes toward vmDAns, with no effects on other neuronal types concomitantly present in cultures.

### Control vmDAns Accumulate α-syn when Co-cultured with PD Astrocytes

Given the relevance of α-syn in the context of PD pathogenesis (Braak et al., 2007), we sought to examine whether vmDAns co-cultured with PD astrocytes abnormally accumulated α-syn. α-syn was barely detectable in the cytoplasm of Ctrl vmDAns cultured alone (data not shown) or when co-cultured with Ctrl astrocytes (Figure 3G). In contrast, Ctrl vmDAns accumulated high levels of α-syn throughout the neurites and cell body after 4 weeks of co-culture with PD astrocytes (Figures 3H and 3I). Notably, while Ctrl astrocytes had undetectable levels of α-syn (Figure 3J), PD astrocytes displayed high levels of α-syn (Figures 3K and 3L), raising the intriguing possibility that α-syn from PD astrocytes might be transferred to Ctrl vmDAns. To directly address if this was the case, we genetically engineered two iPSC lines (representing one PD patient and one healthy control) using CRISPR/Cas9 technology so that the endogenous α-syn would be tagged with a FLAG peptide (α-syn-FLAG iPSC lines; Figures S2K and S2L and Tables S2 and S3). α-syn-FLAG-tagged astrocytes were generated and fully characterized (Figure S2M). As expected, PD α-syn-FLAG tagged astrocytes accumulated abnormally high levels of α-syn, which co-localized with anti-FLAG staining (Figure 3M). More importantly, the co-culture of Ctrl vmDAns on top of α-syn-FLAG-tagged PD astrocytes for 4 weeks resulted in FLAG-tagged α-syn accumulation in neurons, demonstrating the direct transfer of astrocytic α-syn to neurons (Figure 3N).

In addition to co-culturing cells with direct glia-neuron contact, we tested the effect of culturing Ctrl vmDAns with medium conditioned by Ctrl or PD astrocytes at different concentrations (Figure S3A). Exposure of Ctrl vmDAns to PD astrocyte-conditioned medium for 1 week, even at low concentrations, resulted in α-syn accumulation, morphological alterations suggestive of neurodegeneration, and decreased cell survival (Figures S3B–S3H), indicating that PD astrocytes secrete a molecule(s) that is toxic to vmDAns. Direct uptake by vmDAns of α-syn from conditioned medium was tested by exposing Ctrl vmDAns to medium collected from PD α-syn-FLAG-tagged astrocytes (Figures S3I–S3J), suggesting that the neurotoxic effect of PD astrocytes on vmDAns is, at least in part, mediated by secretion of α-syn.

### Control Astrocytes Partially Rescue Neurodegeneration of PD vmDAns

**Figure 4:**
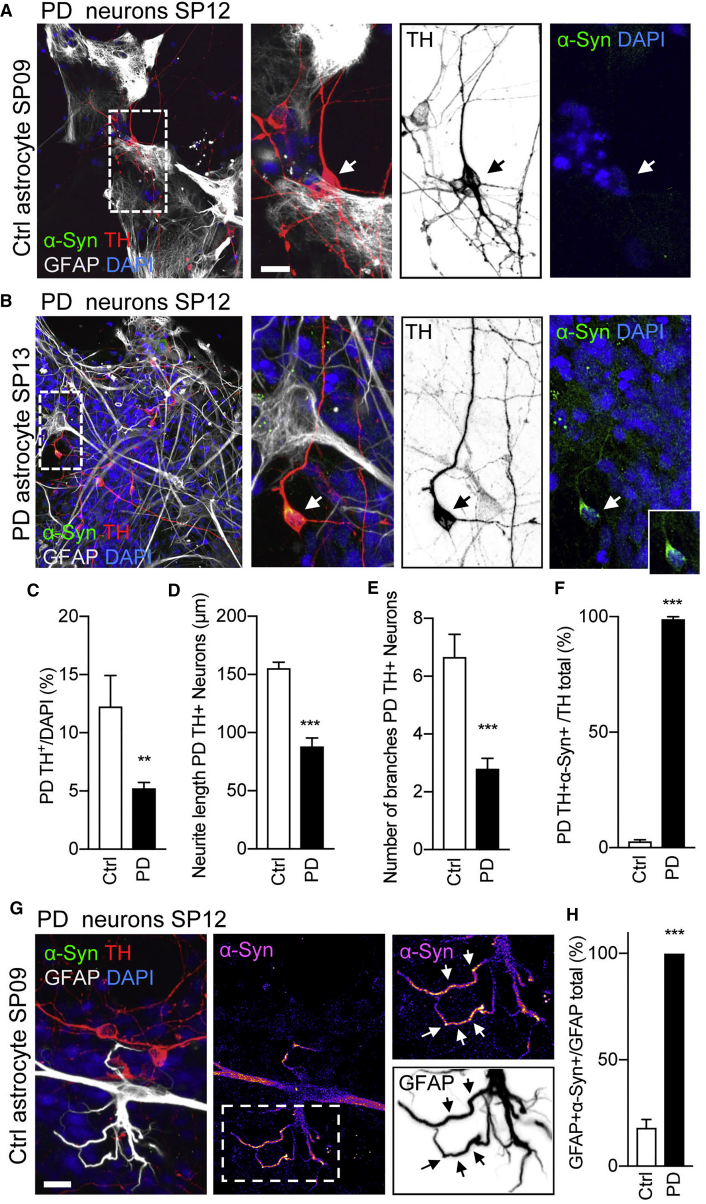
PD Neurons Restore Arborized Morphology and Accumulate Less α-syn when Co-cultured with Ctrl Astrocytes (A and B) Representative ICC images of PD SP12 vmDAns during 4-week co-cultures with (A) Ctrl SP09 or (B) PD SP13 astrocytes stained for TH (vmDAns), α-syn, GFAP (astrocytes), and DAPI. Images on the right show a magnification of the area boxed in the left images. Arrows indicate the selected cell for which an insert is shown at higher magnification with α-syn accumulation. Scale bar, 20 μm. (C) Quantitative analysis of the percentage of PD SP12 vmDAns remaining after 4-week co-culture with Ctrl SP09, PD SP13, and PD SP12 astrocytes (n = 3). (D and E) (D) Neurite length quantification and (E) number of branches of PD SP12 TH-positive neurons when co-cultured on PD SP12 or PD SP13 astrocytes for 4 weeks compared with the wild-type condition Ctrl SP11 neurons on Ctrl SP09, Ctrl SP17, and Ctrl SP11 astrocytes for 4 weeks (n = 3); 40 neurons counted per experiment. (F) Quantitative analysis of the percentage of PD SP12 vmDAns that stained positive for α-syn when co-cultured on the top of Ctrl SP09, Ctrl SP17, Ctrl SP11, PD SP13, and PD SP12 astrocytes for 4 weeks (n = 3). (G) Immunofluorescence analysis of PD SP12 neurons on the top of Ctrl SP09 astrocytes stained for TH, GFAP, α-syn, and DAPI. Images on the right show a magnification of the area boxed in the left image. Arrows in the inset shows α-syn accumulation inside Ctrl SP09 astrocyte processes. Inset scale bar, 20 μm. (H) Quantitative analysis of the percentage of astrocytes that stained positive for α-syn after being cultured with PD SP12 neurons for 4 weeks. Ctrl astrocytes were derived from SP09, SP11, and SP17 iPSCs, while PD astrocytes were derived from SP12 and SP13 iPSCs (n = 3). Data are expressed as mean ± SEM, unpaired two-tailed Student's t test, ^∗∗^p < 0.01, ^∗∗∗^p < 0.001).

We have previously shown that vmDAns derived from PD-iPSCs show signs of neurodegeneration (including reduced numbers of neurites and neurite arborization, as well as accumulation of abnormal α-syn in the soma) after 50 days of culture, which are not evident in Ctrl vmDAns (Sanchez-Danes et al., 2012). To test whether the neurodegeneration could be rescued or prevented by healthy astrocytes, we co-cultured PD vmDAns with Ctrl or PD astrocytes. After a 4-week co-culture, PD vmDAns showed a partially recovered neurite number and complex neurite arborization when co-cultured on control astrocytes, compared with co-cultures with PD astrocytes (Figures 4A–4E). This rescue was partial, since PD vmDAns co-cultured with control astrocytes did not reach the levels of cell survival and complex neurite arborization seen in co-cultures of Ctrl vmDAns and Ctrl astrocytes (compare Figures 4A and 4C–4E with 3B and 4D–4F). Moreover, co-culture with Ctrl astrocytes also prevented the accumulation of α-syn in PD vmDAns that was evident in co-cultures with PD astrocytes (Figures 4A, 4B, and 4F). Notably, most Ctrl astrocytes when co-cultured with PD vmDAns adopted a flat morphology with moderate levels of α-syn; however, some harbored a hypertrophic morphology with retracted processes that accumulated very high levels of α-syn (Figures 4G and 4H), suggesting that reactive astrocytes may contribute to the clearance of vmDAn α-syn accumulation. Culture of PD vmDAns with medium conditioned by Ctrl astrocytes also rescued cell survival, morphological alterations, and α-syn accumulation (Figures S3K–S3S), indicating that direct neuronal-glial contact was not necessary for the neuroprotective effect.

We next investigated the causative role of the genetic background of patient-specific astrocyte cells by ectopically expressing mutated *LRRK2* G2019S in Ctrl astrocytes. In these experiments, Ctrl astrocytes were transfected with a plasmid expressing V5-tagged *LRRK2* G2019S, or with a Ctrl plasmid expressing GFP, and analyzed 7 days after transfection for the presence of α-syn. Astrocytes transfected with *LRRK2* G2019S exhibited diffuse cytoplasmic accumulations of α-syn (Figure S4A), which were not present in GFP-transfected cells (Figure S4B). The transfection efficiencies (30%–40%, as evaluated by co-staining for V5/GFAP or GFP/GFAP) were comparable under both conditions (Figure S4C). Next, we co-cultured Ctrl vmDAns for 4 weeks with *LRRK2* G2019S-transfected Ctrl astrocytes, or with GFP-transfected astrocytes as a control, and we found α-syn accumulation in 50% of the TH^+^ neurons only in co-cultures with *LRRK2* G2019S-transfected astrocytes (Figures S4D–S4G). Overall in these cultures we found decreased survival of vmDAns and evident morphological alterations (Figures S4H–S4I), including fewer and shorter neurites compared with vmDAns cultured on top of GFP-transfected astrocytes, indicating that the expression of pathogenic LRRK2 in Ctrl astrocytes is deleterious for the survival of dopaminergic neurons.

For the converse experiment, we generated isogenic PD astrocytes lacking the *LRRK2* G2019S mutation by CRISPR/Cas9-mediated gene editing of PD iPSCs (iPSC line PD SP13, from here on referred to as PD iso), and fully characterized these cells (Figures S5A–S5E). Abnormal α-syn accumulation did not occur in gene-corrected astrocytes, in contrast with their isogenic mutant counterparts (Figures S5F and S5G). Moreover, co-culturing gene-corrected astrocytes with Ctrl vmDAns for 4 weeks prevented the accumulation of α-syn and decrease in neuron survival observed when Ctrl vmDAns were co-cultured with PD astrocytes (Figures S5H–S5J), further supporting that the expression of mutant *LRRK2* in astrocytes is pathogenic to Ctrl vmDAns.

### Dysfunctional Chaperone-Mediated Autophagy and Progressive α-syn Accumulation in PD Astrocytes

**Figure 5:**
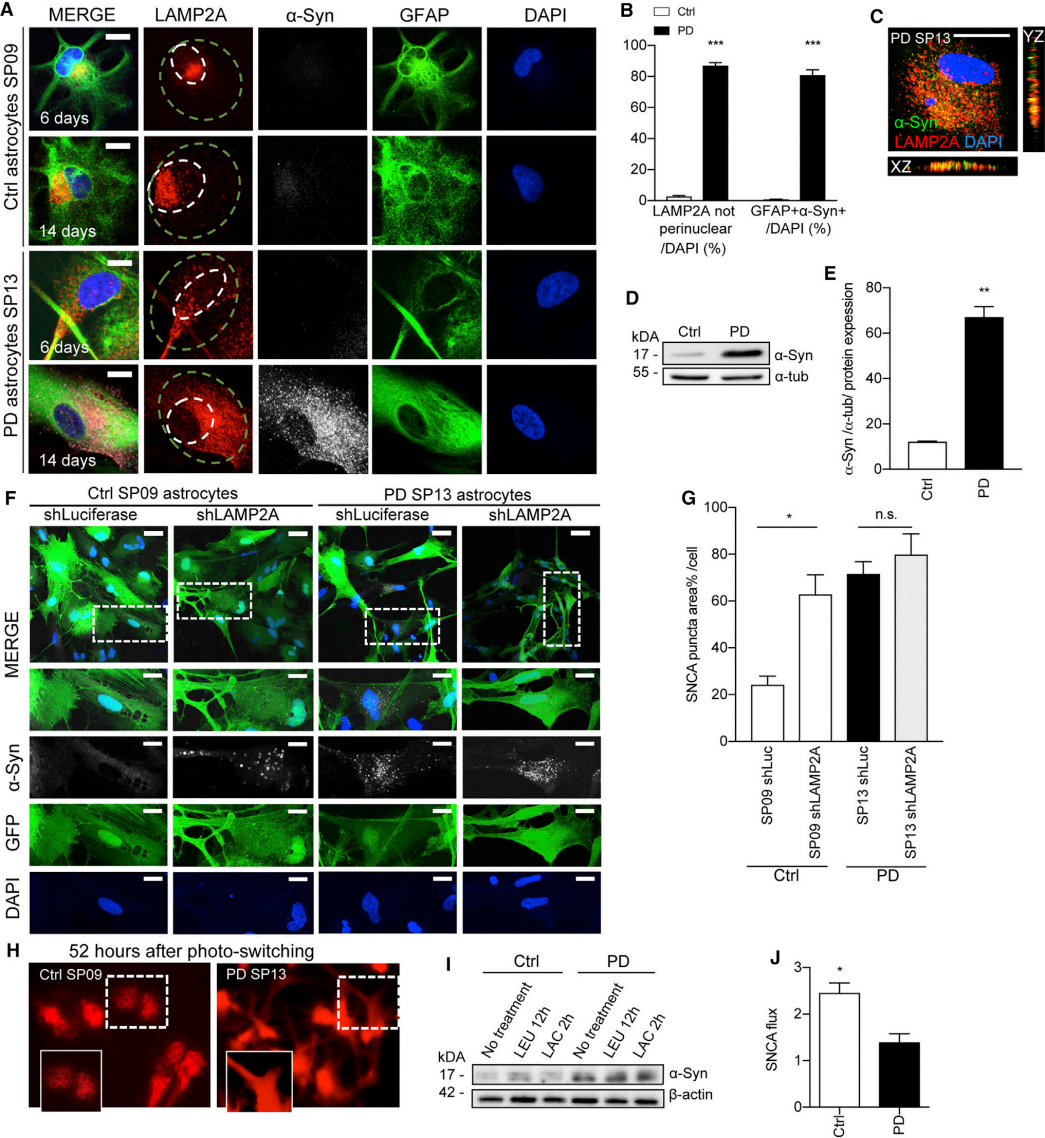
Altered CMA and α-syn Accumulation in LRRK2-PD Astrocytes (A) Representative ICC images of CMA receptor (LAMP2A), astrocyte marker GFAP, α-syn, and nuclear marker DAPI in Ctrl SP09 and PD SP13 astrocytes at 6 and 14 days. Scale bar, 20 μm. Smaller white circles represent perinuclear area, whereas larger green circle represents non-perinuclear area. (B) Percentage of astrocytes with LAMP2A-positive puncta positioning outside of perinuclear area and percentage of astrocytes that stained positive for α-syn. Astrocyte lines used in the experiment were Ctrl SP09, Ctrl SP17, PD SP12, and SP13 (n = 3). (C) Representative ICC image of positive co-localization of LAMP2A and α-syn in PD SP13 astrocytes. Scale bar, 10 μm. (D and E) (D) Western blot of α-syn and α-tubulin as a loading control and (E) quantification in Ctrl SP09 and PD SP13 astrocytes after 14 days in culture (n = 4). (F) Representative ICC images of Ctrl SP09 and PD SP13 astrocytes after 14 days of transduction with either LV-shLAMP2A or LV-shLuciferase (as a control) stained for α-syn, GFP, and DAPI. Boxed areas highlight the region for which high magnification images are shown. Scale bars, 20 and 10 μm, respectively. (G) Percentage of α-syn puncta area per cell in Ctrl SP09 and PD SP13 astrocytes transduced with LV-shLuciferase or LV-shLAMP2A (n = 3). (H) KFERQ-DENDRA (CMA reporter) in Ctrl SP09 and PD SP13 astrocytes 52 hr after photo-switching with UV light (n = 3). Images in the insets at the bottom are a magnification of the boxed area. (I and J) (I) Western blot of α-syn and β-actin as a loading control and (J) quantification of α-syn flux ratio normalized to β-actin in Ctrl SP09 and PD SP13 after the addition of inhibitors of lysosomal proteolysis (leupeptin [LEU], 100 μM) for 12 hr and proteasomal degradation (lactacystin [LAC], 5 μM) for 2 hr (n = 3). Data are expressed as mean ± SEM, unpaired two-tailed Student's t test, ^∗^p < 0.05, ^∗∗^p < 0.01, ^∗∗∗^p < 0.001.

Since PD astrocytes displayed higher levels of α-syn compared with controls, we next investigated possible differences in α-syn turnover in these cells. Degradation of α-syn in lysosomes occurs in large extent through CMA (Cuervo et al., 2004, Martinez-Vicente et al., 2008). To investigate possible changes in CMA in PD astrocytes, we first stained at 6 and 14 days for both α-syn and LAMP2A, the receptor for CMA (Figures 5A and S6A). Ctrl astrocytes showed LAMP2A in the perinuclear area (perinuclear lysosomal positioning occurs during CMA activation; Kiffin et al., 2004) and barely detectable levels of α-syn at both 6 and 14 days (Figures 5A, 5B, and S6A). In contrast, PD astrocytes displayed LAMP2A-positive vesicles all around the cell body as early as 6 days, which continued to be present after 14 days (Figures 5A, 5B, and S6A). Moreover, abnormal accumulation of α-syn was confirmed in PD astrocytes after 14 days of culture, compared with Ctrl astrocytes (Figures 5A–5E). Interestingly, this accumulation was not present after 6 days of culture, suggesting progressive α-syn accumulation over the 14-day time period. Co-localization analyses of α-syn with the LAMP2A receptor revealed a positive co-localization that was more evident in PD astrocytes (Figures 5C and S6B). CMA substrates are usually rapidly internalized and degraded inside lysosomes, but we have previously described a similar persistent association of α-syn with LAMP2A-positive lysosomes in PD models due to blockage in α-syn translocation inside lysosomes (Orenstein et al., 2013). These findings suggest, thus, a similar CMA blockage in the PD astrocytes at the receptor level. Also supportive of reduced α-syn degradation, western blot analysis confirmed a higher monomeric protein level of α-syn in PD astrocytes compared with controls (Figures 5D, 5E, and S7A). By using an antibody that detects specifically oligomeric α-syn, we were able to detect other pathogenic forms of α-syn in PD astrocytes, which were similar to those of PD postmortem brain tissue (Figure S7B).

To investigate the contribution of the defect in CMA to the progressive accumulation of α-syn in PD astrocytes, we next performed a knockdown of LAMP2A using lentiviral-mediated short hairpin RNA (shRNA) targeting and silencing the LAMP2A spliced transcript (shLAMP2A), or an shRNA targeting the Luciferase gene (shLuc) as a control (Figure 5F). The shLuc control astrocytes displayed an expected low level of α-syn, whereas after shLAMP2A transduction, there was a statistically significant (p < 0.001) 2.5-fold increase in α-syn puncta, comparable to the levels observed in PD astrocytes (Figure 5G). Knockdown of LAMP2A did not change α-syn puncta levels in PD astrocytes, further suggesting defective CMA for α-syn in these cells. CMA activity was monitored using a photoactivatable CMA reporter, KFREQ-Dendra (Koga et al., 2011), in all astrocyte lines at 52 hr after photoactivation (Figures 5H and S6C). KFREQ-Dendra is present in the cytosol (diffuse fluorescent pattern) but as it is delivered to lysosomes via CMA it changes to a fluorescent punctate pattern. Ctrl astrocytes displayed these puncta, indicative of functional CMA, whereas the signal in PD astrocytes remained diffused in the cytosol, suggestive of an inactive CMA.

Since PD astrocytes displayed higher levels of α-syn compared with Ctrl astrocytes, we next investigated possible differences in α-syn turnover in these cells. α-syn has previously been shown to undergo degradation both by the ubiquitin/proteasome system and by autophagy (Cuervo et al., 2004, Webb et al., 2003); therefore α-syn flux in the presence of lysosomal and proteasome inhibitors (leupeptin and lactacystin, respectively) was evaluated in Ctrl and PD astrocytes at 14 days (Figures 5I, 5J, and S7D). An increase of ∼40% in α-syn levels was found in Ctrl astrocytes after leupeptin treatment, while this increase was not found in PD astrocytes analyzed under the same conditions, indicating an impaired flux. No changes were found in either Ctrl or PD astrocytes after lactacystin treatment (Figures 5I, 5J, and S7D). These findings suggest major alterations in α-syn proteostasis due to poor degradation by lysosomal systems in PD astrocytes.

### Impaired Macroautophagy in PD Astrocytes

**Figure 6:**
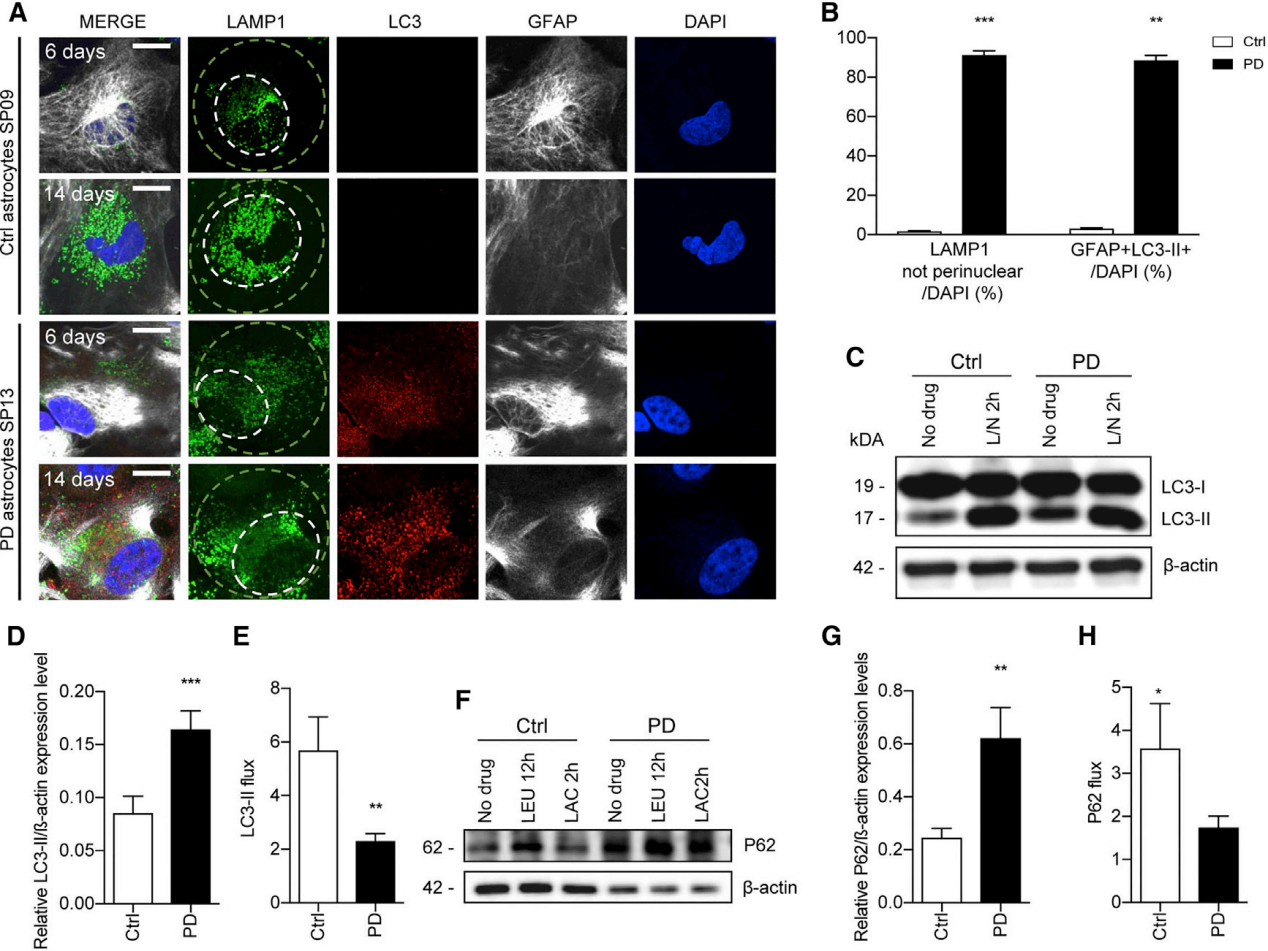
Dysfunctional Macroautophagy in LRRK2-PD Astrocytes (A) Representative ICC images of lysosomal protein marker LAMP1 and autophagosome marker LC3 in Ctrl SP09 and PD SP13 astrocytes (GFAP) at 6 and 14 days. Smaller white circles represent perinuclear area, whereas larger green circle represents non-perinuclear area. Scale bar, 20 μm. (B) Percentage of astrocytes with LAMP1-positive puncta positioning outside of perinuclear area and percentage of astrocytes that stained positive for LC3-II. Astrocyte lines used in the experiment were Ctrl SP09 and SP13 (n = 3). (C–E) (C) Western blot of LC3-II protein levels and β-actin as loading control with corresponding quantification of (D) the LC3-II basal expression and (E) LC3-II flux with or without lysosomal inhibitors NH_4_Cl and leupeptin (L/N) for 2 hr in Ctrl SP09 and PD SP13 astrocytes (n = 3). (F–H) (F) Western blot of p62 protein levels and β-actin as loading control with corresponding quantification of (G) the P62 basal expression and (H) P62 flux without inhibitors or with inhibitors leupeptin for 12 hr and lactacystin for 2 hr in Ctrl SP09 and PD SP13 astrocytes (n = 3). Data are expressed as mean ± SEM, unpaired two-tailed Student's t test, ^∗^p < 0.05, ^∗∗^p < 0.01, ^∗∗∗^p < 0.001.

Cells often respond to blockage in CMA by upregulating other autophagic pathways such as macroautophagy (Massey et al., 2006, Schneider et al., 2015). However, altered macroautophagy has also been reported in the context of PD (Sanchez-Danes et al., 2012, Winslow et al., 2010). To investigate the status of macroautophagy, the endo/lysosomal marker LAMP1, autophagosome marker LC3, astrocyte marker GFAP, and nuclear DAPI were used during ICC on all astrocyte lines at both 6 and 14 days. In Ctrl astrocytes, there was lysosomal LAMP1 staining in the perinuclear area and very few visible autophagic vacuoles (LC3-positive vesicles) at both 6 and 14 days (Figures 6A, 6B, and S6D). In PD astrocytes, as for LAMP2A, LAMP1-positive vesicles lost the preferable perinuclear distribution and were found throughout the entire cell (Figures 6A, 6B, and S6D). In addition, there was a marked increase in autophagic vacuoles starting as early as 6 days that continued increasing through the 14-day time point (Figures 6A, 6B, and S6D). Most of the accumulated LC3-positive vesicles in PD astrocytes did not co-localize with LAMP1 lysosomes (Figures S6E and S6F), suggesting that they were autophagosomes that persisted in these cells due to their poor clearance by lysosomes.

In agreement with the immunofluorescence studies, western blot analyses detected higher basal levels of LC3-II in PD astrocytes compared with Ctrls (Figures 6C, 6D, and S7C). To monitor the autophagy flux and to gain insights into the mechanism behind the accumulated LC3-II levels in PD astrocytes, both Ctrl and PD astrocytes were treated with leupeptin and NH_4_Cl, inhibitors of lysosomal proteolysis, to inhibit LC3-II degradation. Under these conditions, PD astrocytes exhibited lower increase in LC3-II levels compared with controls, suggesting an impairment of the autophagy flux in PD astrocytes (Figures 6E and S7C). Moreover, we found higher p62 levels in PD astrocytes at baseline compared with controls, and lower flux ratio in the presence of inhibitor (Figures 6F–6H and S7E). Overall these findings suggest that reduced function in both autophagic pathways, CMA and macroautophagy, contribute to the altered α-syn proteostasis observed in PD astrocytes.

### Restoration of α-syn Proteostasis in PD Astrocytes Alone and during Co-culture of Control Neurons with PD Astrocytes

**Figure 7:**
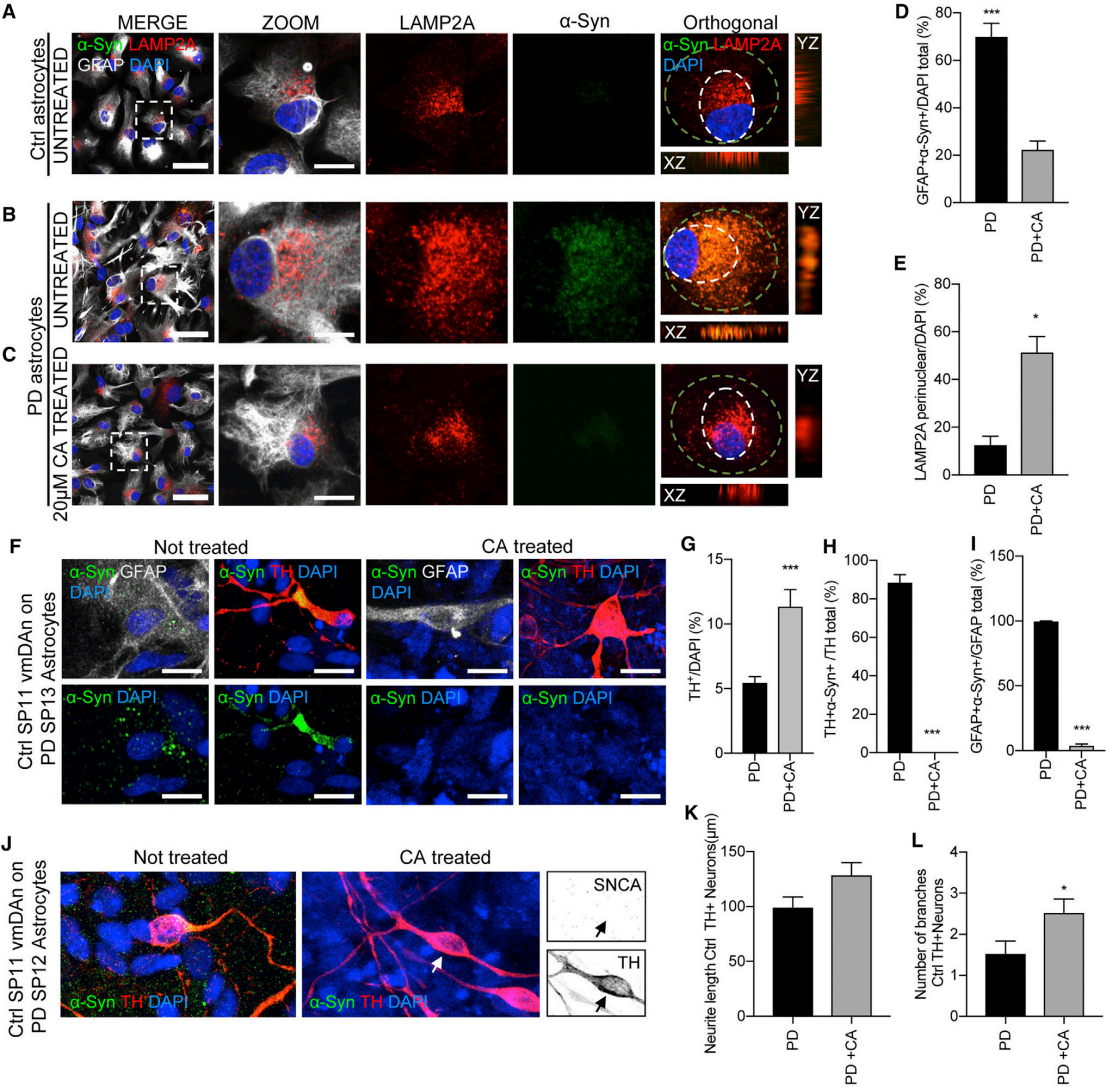
CMA Activator Drug Treatment Rescues α-syn Accumulation in Neurons Cultured with PD Astrocytes (A and B) Representative ICC images of (A) Ctrl and (B) PD astrocytes after 2 weeks in culture without treatment stained for LAMP2A, α-syn, GFAP, and DAPI. Orthogonal views reveal positive co-localization of α-syn to LAMP2A in PD untreated sample. Images on the right show a magnification of the area boxed in the left images. Dashed circles outline the perinuclear area of the cell. Scale bars, 100 and 20 μm in merge and zoom, respectively. (C) Representative ICC images of PD astrocytes after 20 μg of CA drug treatment stained for LAMP2A, α-syn, GFAP, and DAPI. Scale bars, 100 and 20 μm in merge and zoom, respectively. (D and E) (D) Quantitative analysis of PD astrocytes (SP13 and SP12) stained positive for α-syn either not treated or treated with CA; (E) quantitative analysis of the same astrocytes under the same conditions, showing LAMP2A puncta in the perinuclear area (n = 3). (F) Representative ICC images of 4-week Ctrl SP11 vmDAns co-cultured on PD SP13 astrocytes (left) or treated with CA for 2 weeks (right). Samples were stained for GFAP, TH, α-syn, and DAPI. Scale bars, 20 μm. (G–I) (G) Quantitative analysis of the percentage of vmDAns remaining after being co-cultured with PD SP12 or PD SP13 (without treatment or treated with CA) for 4 weeks. Percentage of the (H) vmDAns or (I) astrocytes that stained positive for α-syn 4 weeks after the same co-culture conditions (n = 6). (J) Representative ICC images of 4-week Ctrl SP11 vmDAns co-cultured on PD SP12 astrocytes with or without CA treatment for 2 weeks. Samples were stained for TH, α-syn, and DAPI. Arrows indicate the absence of α-syn accumulation in the selected TH-positive cell. Scale bars, 20 μm. (K and L) (K) Neurite length quantification and (L) number of branches of 4-week Ctrl SP11 vmDAns when co-cultured on PD SP12 or PD SP13 astrocytes with or without CA treatment for 2 weeks (n = 4); 20 neurons counted per experiment. Data are expressed as mean ± SEM, unpaired two-tailed Student's t test, ^∗^p<0.05; ^∗∗∗^p < 0.001.

Intracellular accumulation of α-syn has been shown to contribute to cellular toxicity in PD and to further disrupt functioning of cellular proteostasis systems (reviewed in Abeliovich and Gitler, 2016). We next investigated whether α-syn accumulation in PD astrocytes could be ameliorated by enhancing lysosomal activity. PD astrocytes were treated with a CMA activator (CA), which operates through the release of the endogenous inhibition of the retinoic receptor-α signaling pathway over CMA (Anguiano et al., 2013). Cells were treated with a concentration of 20 μM for 5 days and levels of α-syn were analyzed by immunofluorescence (Figure 7). LAMP2A-positive lysosomes in PD astrocytes treated with the CA (Figures 7C–7E) recovered the perinuclear distribution observed in Ctrl cells (Figure 7A) compared with when not treated (Figure 7B), suggesting reactivation of CMA in these cells. Consistent with higher CMA activity, CA-treated cells had significantly lower α-syn content than untreated cells (Figures 7C–7E).

In addition, we treated PD astrocytes when in co-culture with Ctrl neurons (Figure 7F). Under untreated conditions, Ctrl neurons accumulate astrocytic α-syn and show signs of neurodegeneration. Interestingly, the treatment with CA cleared out α-syn not only in astrocytes, but also in vmDAns, partially restored neuron survival, and decreased the number of TH-positive cells with a degenerative morphology (Figures 7G–7L). These findings suggest that although multiple protein degradation pathways fail to efficiently degrade α-syn in PD cells, reactivation of one of these pathways, in our case CMA, is able to restore functional α-syn proteostasis.

## Discussion

Astrocytes from three PD patients carrying the G2019S mutation in the *LRRK2* gene and two healthy individuals were successfully generated using a previously published protocol and fully characterized. By implementing a patient iPSC-based co-culture model, in this study we describe a role for PD astrocytes in midbrain neuronal cell death. Specifically, in a 4-week co-culture system, we found morphological alterations resembling those of neurodegeneration, such as short and few neurites as well as beaded necklace-like neurites, typically observed in neurons upon transport alterations (Fu et al., 2005, Garrido et al., 2011), and increased neuronal loss in Ctrl neurons co-cultured with PD astrocytes. We interpret these altered phenotypes as representing PD astrocyte-induced vmDAn neurodegeneration. An alternative explanation could be that PD astrocytes impinged on the differentiation and/or maturation of DAn progenitors in our iPSC-derived co-culture system. However, while we cannot formally rule out this possibility, two lines of evidence strongly argue against it playing a significant role in the phenotypes described here. First, we used vmDA neural differentiation cultures at 35 days of differentiation for our co-culture experiments. At this time, most vmDAns are already committed in fate (TH^+^/FOXA2^+^, see Figure 2A), but are still at a stage of maturation that does not compromise their survival upon cell dissociation and plating on top of the astrocyte cultures. Second, the numbers of vmDAns at different time points along the co-culture experiments showed progressive decline in co-cultures with PD astrocytes, but no significant changes when co-cultured with Ctrl astrocytes (Figure S2C). These results indicate that few new TH^+^ neurons are generated during co-culture, and further support that the decreased numbers of vmDAns observed upon co-culture with PD astrocytes are a consequence of vmDAn degeneration. Importantly, the altered phenotypes were specifically observed in the subpopulation of midbrain dopaminergic neurons, as numbers of MAP2^+^/TH^−^ neurons did not change significantly upon co-culture with Ctrl or PD astrocytes. In accordance with this, it has been already shown that α-syn toxicity was responsible for nigrostriatal neuronal cell death in midbrain cultures (Petrucelli et al., 2002), a relevant finding regarding the particular vulnerability of nigral neurons in PD. However, it remains to be tested whether, in prolonged culture, PD astrocytes also impair the survival of TH^−^ populations.

Postmortem brain tissue of PD patients revealed α-syn accumulation in astrocytes (Wakabayashi et al., 2000). It has been previously described that astrocytes accumulate neuronal-derived α-syn as a mechanism of neuroprotection (Booth et al., 2017). Indeed, in our study we found that Ctrl astrocytes accumulate α-syn when co-cultured with PD neurons and partially rescued the morphological phenotype of neurodegeneration and clearance of neuronal α-syn. This behavior suggests a neuroprotective effect via inflammatory-mediated activation of the Ctrl astrocytes. In addition, by using a CRISPR/Cas9 gene-edited cell line tagging the endogenous *SNCA* locus with an FLAG tag, our results reveal that PD astrocytes also accumulate and transfer α-syn to the surrounding neurons, suggesting that astrocytes actively contribute to the distribution of α-syn.

Taking into account that our PD astrocytes come from patients carrying the *LRRK2* G2019S mutation, we investigated whether disease-specific phenotypes related to the mutation were present. The α-syn accumulation in our co-culture system indicated a disruption in the way α-syn is usually degraded in PD astrocytes. Degradation of α-syn has been shown to occur by both proteasome and autophagic pathways, and conversely, high levels of α-syn have been demonstrated to be toxic for both systems (Tanaka et al., 2001, Webb et al., 2003, Winslow et al., 2010). Here we found that lysosomal degradation of α-syn was severely inhibited in PD astrocytes. We have previously described in PD neurons that one of the early events in the dysfunction of the proteostasis systems in these cells is the disruption of CMA by mutant LRRK2 binding to the LAMP2A, thus causing the accumulation of α-syn (Orenstein et al., 2013). Here we demonstrate that CMA is also altered in PD astrocytes and confirm that α-syn degradation by CMA in these cells was almost completely abolished.

The increase in intracellular levels of α-syn, due to its poor degradation in PD astrocytes by CMA, may contribute to precipitating malfunctioning of other proteostasis mechanisms, such as the proteasome and macroautophagy. In fact, we demonstrated that macroautophagy was also markedly impaired in these cells, by showing higher basal levels of autophagic vacuoles (LC3-II) and the autophagic cargo p62, and reduced autophagic flux (for both LC3-II and p62). The lower co-localization between the autophagosomal and lysosomal markers observed in PD astrocytes suggests that the reduced autophagic flux is due to a defect in autophagosome/lysosome fusion, similar to that previously described in PD neurons.

Taking into account the coordinate functioning of the proteolytic systems, and the fact that CMA disruption seems to occur early during the development of PD pathology, we attempted to restore normal α-syn proteostasis by enhancing CMA activity. Our findings in cells treated with a chemical activator of CMA suggest that upregulation of CMA is still possible in these cells and that this intervention is sufficient to return levels of α-syn close to those in Ctrl cells. Although α-syn was cleared, restoration during a co-culture with Ctrl neurons was only partial in terms of neurite length and number, suggesting that the neurodegeneration observed could also be due to other non-α-syn-related factors secreted by PD astrocytes.

Overall, our findings propose a specific role for astrocytes in mediating dopaminergic cell death during PD. PD-specific phenotypes specifically related to dysfunctions in the pathways of protein degradation have been observed in PD astrocytes and not in Ctrl astrocytes. Dysfunctional CMA, progressive α-syn accumulation, and glia-to-neuron transfer found in our PD astrocytes are all aspects that can compromise neuronal survival during PD pathogenesis. Future studies will identify whether additional factors other than α-syn are being secreted by (or lacking in) PD astrocytes, and thus contributing to triggering vmDA neuronal cell death. iPSC-based technology allows for the proper recapitulation of patient-specific disease-related phenotypes, which will aid in the discovery of new therapies.

## Experimental Procedures

Experimental procedures are also provided in Supplemental Information.

### iPSC-Derived Astrocyte Generation and Culture

The parental iPSC lines used in our studies were previously generated and fully characterized (Sanchez-Danes et al., 2012). The generation and use of human iPSCs in this work were approved by the Spanish competent authorities (Commission on Guarantees concerning the Donation and Use of Human Tissues and Cells of the Carlos III National Institute of Health). iPSCs were differentiated into astrocytes following a previously published protocol (Serio et al., 2013). See Supplemental Information for more details.

### iPSC-Derived vmDAn Generation

Four different iPSCs, two PD (SP12 and SP13) and two Ctrl (SP11 and SP11#4), were differentiated into dopaminergic neurons using a combination of two previously published protocols for midbrain induction (Chambers et al., 2009, Kriks et al., 2011). Detailed methods are provided in Supplemental Information.

#### ICC

ICC on cell cultures was performed as described in Supplemental Information.

#### Statistical Analysis

Statistical analyses of the obtained data were performed using two-tailed unequal variance Student’s t tests and ANOVA (^∗^p < 0.05, ^∗∗^p < 0.01, ^∗∗∗^p < 0.001), and the mean and standard error of the mean were plotted using Prism (Mac OS X). Number of independent experiments (n) is indicated in each figure legend.

## Author Contributions

Conceptualization, A.C. and A.R.; Methodology, I.F., J.P.M., A.Z., A.M.C., and J.S.; Investigation, Y.R.P, A.D., G.C., C.C., M.P.-E., M.G., I.F.-C., J.P., and A.F.; Validation, A.C. and A.R.; Writing – Original Draft, A.D.; Writing – Review & Editing, A.C. and A.R.; Visualization, A.D.; Resources, A.Z., A.M.C., J.S., E.T., A.C., and A.R.; Funding Acquisition, A.C.; Supervision, A.C.

## Supporting Citations

The following references appear in the Supplemental Information: Alwin et al., 2005, Kita-Matsuo et al., 2009, Massey et al., 2008, Mussolino et al., 2011, Park et al., 2015.

## Supplemental Information

Document S1. Supplemental Experimental Procedures, Figures S1–S7, and Tables S1–S3

Document S2. Article plus Supplemental Information
