# Supplementary material for: Patient-Specific iPSC-Derived Astrocytes Contribute to Non-Cell-Autonomous Neurodegeneration in Parkinson's Disease
Source: Stem Cell Reports. 2019 Jan 10;12(2):213–29. doi: 10.1016/j.stemcr.2018.12.011 (PMC6372974; doi:10.1016/j.stemcr.2018.12.011)
Supplement: Document S1. Supplemental Experimental Procedures, Figures S1–S7, and Tables S1–S3 [file mmc1.pdf]

Stem Cell Reports, Volume *12*

## Supplemental Information

# Patient-Specific iPSC-Derived Astrocytes Contribute to Non-Cell-Autonomous Neurodegeneration in Parkinson's Disease

**Angelique di Domenico, Giulia Carola, Carles Calatayud, Meritxell Pons-Espinal, Juan Pablo Muñoz, Yvonne Richaud-Patin, Irene Fernandez-Carasa, Marta Gut, Armida Faella, Janani Parameswaran, Jordi Soriano, Isidro Ferrer, Eduardo Tolosa, Antonio Zorzano, Ana Maria Cuervo, Angel Raya, and Antonella Consiglio**

**Supplementary Information**

# Patient-specific iPSC-derived astrocytes contribute to non-cell autonomous neurodegeneration in Parkinson's disease

Angelique di Domenico,[1,2,14] Giulia Carola,[1,2,14] Carles Calatayud,[1,2,3] Meritxell Pons-Espinal,[1,2] Juan Pablo Muñoz,[4] Yvonne Richaud-Patin,[3,5] Irene Fernandez-Carasa,[1,2] Marta Gut,[6] Armida Faella,[1,2] Janani Parameswaran,[1,2] Jordi Soriano,[7,8] Isidro Ferrer,[2,9] Eduardo Tolosa,[9,10] Antonio Zorzano,[4] Ana Maria Cuervo,[11] Angel Raya, [3,5,12,*] Antonella Consiglio[1,2,13,*,‡]

[1] Department of Pathology and Experimental Therapeutics, Bellvitge University Hospital-IDIBELL, 08908 Hospitalet de Llobregat, Spain.

[2] Institute of Biomedicine of the University of Barcelona (IBUB), Barcelona 08028, Spain.

[3] Center of Regenerative Medicine in Barcelona (CMRB), Hospital Duran i Reynals, Hospitalet de Llobregat, 08908 Barcelona, Spain.

[4] Institute for Research in Biomedicine (IRB), Barcelona 08028, Spain

[5] Centre for Networked Biomedical Research on Bioengineering, Biomaterials and Nanomedicine (CIBER-BBN), Madrid 28029, Spain.

[6] Centre Nacional d'Anàlisi Genòmica (CNAG-CRG), Parc Científic de Barcelona, Barcelona 08028, Spain.

[7] Departament d'Estructura i Constituents de la Matèria, Universitat de Barcelona, Barcelona 08028, Spain.

[8] Universitat de Barcelona Institute of Complex Systems (UBICS), Barcelona 08028, Spain.

[9] Centre for Networked Biomedical Research on Neurodegenerative Diseases (CIBERNED), Madrid 28049, Spain.

[10] Department of Neurology, Hospital Clínic de Barcelona, Institut d'Investigacions Biomèdiques August Pi i Sunyer (IDIBAPS), University of Barcelona (UB), Barcelona 08036, Spain.

[11] Albert Einstein College of Medicine, 300 Morris Park Ave, Bronx, NY 10461, United States

[12] Institució Catalana de Recerca i Estudis Avançats (ICREA), Barcelona 08010, Spain

[13] Department of Molecular and Translational Medicine, University of Brescia, Brescia 25121, Italy

[14] Co-first author

* Correspondence should be addressed to araya@cmrb.eu (A.R.) OR consiglio@ub.edu (A.C.)

‡ Lead contact

*Equal contribution

**Supplementary Information contains:**

**3 Supplementary Tables:**

**Table S1**. Patient information used in the study. Healthy donors are referred to: SP09, SP11 and SP17. *LRRK2* G2019S are referred to: SP06, SP12 and SP13. Related to Figure 1.

**Table S2.** Summary of the characterization of iPSC lines used in this study. Related to Table 1 and all the Figures.

**Table S3.** Summary of phenotypes observed in the co-cultures experiments. Duration of the co-culture, neurons and astrocytes viability, α-syn accumulation and cell morphology are described in all conditions tested. Related to Figure 3 and 4.

**7 Supplementary Figures**

Fig. S1. iPSC derived astrocyte characterization. Related to Fig. 1.

Fig. S2. Ctrl vmDAn neuronal morphology upon co-cultured with PD astrocytes and viability analysis and CRISPR/Cas9 gene editing strategy tagging *SNCA* locus with FLAG tag. Related to Fig. 3.

Fig. S3. Effects of Ctrl and PD/PD-FLAG astrocyte conditioned medium on α-synuclein accumulation in Ctrl vmDA neurons. Related to Fig. 3 and 4.

Fig. S4. Effect of *LRRK2* G2019S transfected in control astrocytes. Related to Fig. 3.

Fig. S5. Schematic description of the generation of isogenic control line and phenotypic comparison between isogenic clones, alone or in co-culture with Ctrl-SP11 neurons. Related to Fig. 3 and 4.

Fig. S6. Altered CMA, dysfunctional macroautophagy and α-syn accumulation in PD astrocytes. Related to Fig. 5. and 6.

Fig. S7. Original western blots. Related to Fig. 5 and 6.

**Supplementary Experimental Procedures**

**Table S1. Patient Information**

| Subject Identifier (**SP**) | *LRRK2* G2019S Patients | | | Healthy Donors | | |
|---|---|---|---|---|---|---|
| | **SP12** | **SP13** | **SP06** | **SP09** | **SP17** | **SP11** |
| **Age at donation** | 63 | 68 | 44 | 66 | 52 | 48 |
| **Age at diagnosis** | 50 | 57 | 33 | N/A | N/A | N/A |
| **Gender** | Female | Female | Male | Male | Male | Female |
| **Ethnicity** | Caucasian | Caucasian | Caucasian | Caucasian | Caucasian | Caucasian |
| **Mutation** | G2019S | G2019S | G2019S | No | No | No |
| **PD diagnosis** | Criteria - fPD | Criteria - fPD | Criteria - fPD | No | No | No |
| **Family history** | Yes | Yes | Yes | N/A | N/A | N/A |
| **Unilateral onset** | Yes | Yes | Yes | N/A | N/A | N/A |
| **Resting tremor** | Yes | Yes | Yes | N/A | N/A | N/A |
| **Progressive disease** | Yes | Yes | Yes | N/A | N/A | N/A |
| **L-dopa responsive** | Yes | Yes | Yes | N/A | N/A | N/A |
| **L-dopa responsive >5yrs** | Yes | Yes | Yes | N/A | N/A | N/A |
| **L-dopa induced Chorea** | Yes | No | Yes | N/A | N/A | N/A |
| **Clinical course >10yrs** | Yes | Yes | Yes | N/A | N/A | N/A |
| **Head trauma** | No | No | No | N/A | N/A | N/A |
| **Dementia** | No | No | No | N/A | N/A | N/A |
| **Deep brain stimulation** | No | No | Yes | N/A | N/A | N/A |
| **Others** | Non-smoker. No alcohol consumption | Coffee drinker. Non-smoker. No alcohol consumption. No anti-inflammatory treatment | Coffee drinker. Smoker. No anti-inflammatory treatment | N/A | N/A | N/A |
| **Treatment** | Neupro 12mg, Pantoprazol 40mg, Mirapexin, Hidroferol, Sinemet Plus, Hidroclorotiazida | Digoxina, BoiK, Dinisor Retard, Mirapexin, Omeprazol, Sinemet Plus, Sintrom | Stalevo, amantadine, Rivotril, amitriptilina | N/A | N/A | N/A |

*fPD = familial PD

**Table S2. Summary of the characterization of iPSC lines used in this study. Related to Table 1 and all Figures**

| iPSC line | Reprogramming method | # iPSC clone generated | Morphology | Pluripotency gene expression[1] | Pluripotency protein expression[2] | Three germ layers[3] | Karyotype | Reference | # astrocyte generation |
|---|---|---|---|---|---|---|---|---|---|
| **Ctrl SP09** | Retrovirus | 4 | + | + | + | + | Normal | Sanchez Danes (EMBO Mol Med. 2012) | 3 |
| **Ctrl SP11** | Retrovirus | 4 | + | + | + | + | Normal | Sanchez Danes (EMBO Mol Med. 2012) | 3 |
| **Ctrl SP11#4** | Retrovirus | 4 | + | + | + | + | Normal | Sanchez ıes (EMBO Mol Med. 2012) | 3 |
| **Ctrl SP11- α-syn-FLAG** | N/A | 3 | + | + | + | + | Normal | This study | 2 |
| **Ctrl SP17** | Retrovirus | 4 | + | + | + | + | Normal | Sanchez Danes (EMBO Mol Med. 2012) | 3 |
| **PD SP06** | Retrovirus | 4 | + | + | + | + | Normal | Sanchez Danes (EMBO Mol Med. 2012) | 3 |
| **PD SP12** | Retrovirus | 4 | + | + | + | + | Normal | Sanchez Danes (EMBO Mol Med. 2012) | 3 |
| **PD SP12-α-syn-FLAG** | N/A | 3 | + | + | + | + | Normal | This study | 2 |
| **PD SP13** | Retrovirus | 4 | + | + | + | + | Normal | Sanchez Danes (EMBO Mol Med. 2012) | 3 |
| **PD Iso** | N/A | 3 | + | + | + | + | Normal | This study | 2 |

[1]Expression levels of *OCT4*, *SOX2*, *NANOG* and *LIN28* by qRT-PCR

[2]Expression of OCT4, NANOG, TRA 1-81 and SSEA4 by immunocytochemistry

[3]Expression of AFP (endoderm), SMA (mesoderm) and TUJ1 (ectoderm) by immunocytochemistry

**Table S3. Summary of Phenotypes During Co-cultures**

| Co-culture | | | Phenotypes** | | | | | | | | |
|---|---|---|---|---|---|---|---|---|---|---|---|
| | | | | | | | Cell Morphology | | | | |
| Line | | Duration | Cell viability (fold change*) | | Alpha-synuclein accumulation (%/total cell type) | | Neuron | | | Astrocyte | |
| Neuron | Astrocyte | weeks | Neuron | Astrocyte | Neuron | Astrocyte | Status | Average neurite intersection #/neuron | Average neurite length/neuron | Resting (flat, large) | Reactive (retracted) |
| SP11 | SP09 | 2 | 1 | 1 | 0 | 0 | immature | N/A | N/A | 100% | 0 |
| | SP09 | 4 | 1 | 1 | 0 | 0 | normal | 10 | 304 microns | 100% | 0 |
| | SP17 | 4 | 1 | 1 | 0 | 0 | normal | 10 | 311 microns | 100% | 0 |
| | isoSP13 | 4 | 0,5 | 5,6 | 7% | 38% | degenerated | 1 | 112 microns | 100% | 0 |
| | SP11 α-syn-FLAG | 4 | 1 | 1 | 0 | 0 | normal | 10 | 302 microns | 100% | 0 |
| | SP06 | 4 | 0,4 | 3 | 46% | 81% | degenerated | 1 | 80,3 microns | 0 | 100% |
| | SP13 | 2 | 0,5 | 1 | 0 | 100% | immature | N/A | N/A | 0 | 100% |
| | SP13 | 4 | 0,2 | 1,1 | 45% | 100% | degenerated | 2 | 108 microns | 0 | 100% |
| | SP12 | 4 | 0,2 | 1,6 | 59% | 100% | degenerated | 2 | 101 microns | 0 | 100% |
| | SP12 α-syn-FLAG | 4 | 0,3 | 1,5 | 57% | 100% | degenerated | 2 | 103 microns | 0 | 100% |
| | pDEST51-LRRK2-SP09 | 4 | 0,5 | 1,4 | 50% | 100% | degenerated | N/A | N/A | 0 | 100% |
| | pDEST51-LRRK2-SP17 | 4 | 0,6 | 1,6 | 53% | 100% | degenerated | N/A | N/A | 0 | 100% |
| SP11#4 | SP06 | 4 | 0,2 | 0,6 | 100% | 94% | degenerated | 1,5 | 138 microns | 0 | 100% |
| | SP13 | 4 | 0,1 | 0,3 | 97% | 72% | degenerated | 1 | 96 microns | 0 | 100% |
| SP12 | SP09 | 2 | 1 | 1 | 0 | 0 | immature | N/A | N/A | 100% | 0 |
| | SP09 | 4 | 1,5 | 1 | 0 | 18% | normal | 5 | 155 microns | 82% | 18% |
| | SP11 α-syn-FLAG | 4 | 1,5 | 1 | 0 | 12% | normal | 5 | 162 microns | 88% | 12% |
| | SP13 | 2 | 0,5 | 1,2 | 100% | 100% | immature | N/A | N/A | 0 | 100% |
| | SP13 | 4 | 0,2 | 1,5 | 100% | 100% | degenerated | 2 | 88 microns | 0 | 100% |
| | SP12 | 4 | 0,3 | 1,5 | 100% | 100% | degenerated | 2 | 85 microns | 0 | 100% |
| | SP12 α-syn-FLAG | 4 | 0,2 | 1,5 | 100% | 100% | degenerated | 2 | 91 microns | 0 | 100% |
| SP13 | isoSP13 | 4 | 0,2 | 8,4 | 5% | 60% | degenerated | 1 | 127,5 microns | 100% | 0 |

* cell number normalized to average of both Ctrl astrocytes (SP09 and SP17) per condition
** average of all independent tripicates

| Legend | |
|---|---|
| (light grey) | Ctrl (control) |
| (green) | PD (mutant) |

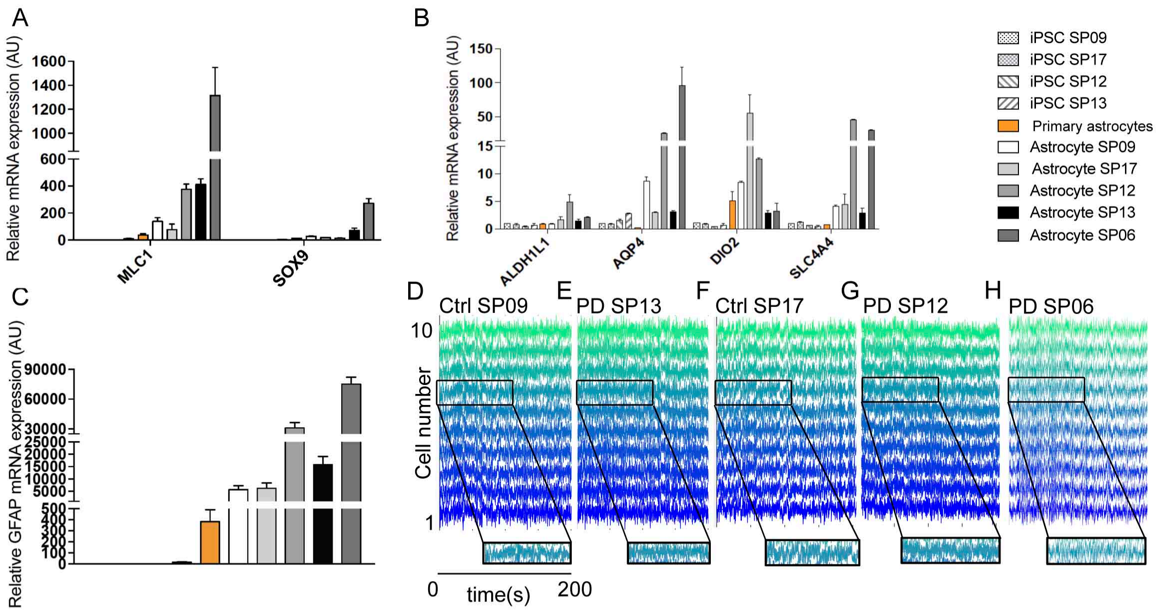


**Figure S1. iPSC-derived astrocyte characterization.**

(A-C) qRT-PCR analysis revealing comparable levels of *MLC1, SOX9, ALDH1L1, AQP4, DIO2, SLC4A4, GFAP* mRNA expression in Ctrl (SP09 and SP17) and PD (SP12, SP13 and SP06) iPSC-derived astrocytes, compared to human primary astrocytes and their corresponding iPSC.

(D-H) Graph representing single functional astrocyte calcium waves of Ctrl (SP09 and SP17) and PD (SP12, SP13 and SP06) astrocytes (n=3).

Data are expressed as mean ± s.e.m, unpaired two-tailed Student's t-test, *p<0.05, **p<0.01, ***p<0.001.

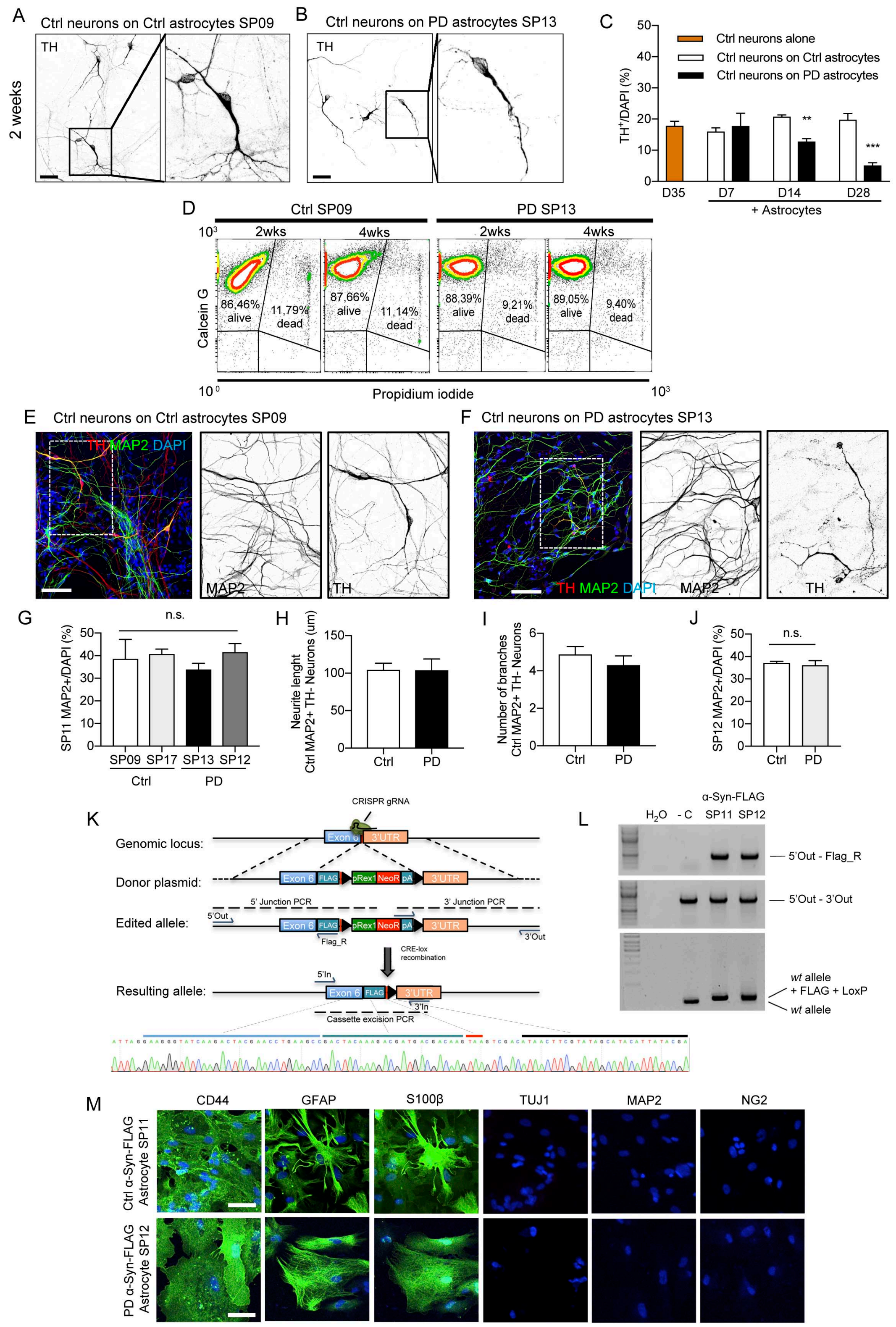


**Figure S2. Ctrl vmDAn neuronal morphology upon co-cultured with PD astrocytes and viability analysis and CRISPR/Cas9 gene editing strategy tagging *SNCA* locus with FLAG tag.**

(A-B) Representative images staining Ctrl SP11 vmDAn (TH, in black) on the top of Ctrl SP09 astrocytes (A) or PD SP13 astrocytes (B) during a 2-week co-culture period. Scale bar 20µm.

(C) Percentage of TH+/DAPI Ctrl SP11 neurons alone (orange bar) or when co-cultured with Ctrl SP09,

Ctrl SP17 or PD SP12, PD SP13 astrocytes after 7, 14 or 28 days of co-culture (n=3).
(D) Contour plot of Ctrl SP09 and PD SP13 astrocyte viability at 2 and 4 weeks.
(E-F) Representative image showing Ctrl SP11 vmDAn on the top of Ctrl SP09 (E) astrocytes or PD SP13 (F) astrocytes during a 4-week co-culture period. Samples were stained for TH, MAP2, and DAPI. Scale bar 20μm.
(G) Quantitative analysis of Ctrl SP11 neurons (MAP2 positive) remaining after 4 weeks in co-culture with Ctrl SP09, Ctrl SP17, PD SP12, or PD SP13 astrocytes (n=3).
(H-I) Quantification of the neurite length (H) and number of branches (I) of MAP2 positive TH negative neurons after 4 weeks of co-culture (n=3); total neurons counted per experiment n=30.
(J) Quantitative analysis of PD SP12 neurons (MAP2 positive) remaining after 4 weeks in co-culture with Ctrl SP09 or PD SP13 astrocytes (n=3).
Data are expressed as mean ± s.e.m, unpaired two-tailed Student's t-test, ***$p<0.001$.
(K) Scheme of the knock-in strategy of a FLAG-tag into the C terminus of the endogenous *SNCA* gene using CRISPR/Cas9. Blue arrows represent the primers used for the PCR screening procedure. Black triangles represent LoxP sites surrounding the selection cassette.
(L) PCR analysis of α-syn-FLAG knock-in iPSC lines Ctrl SP11 and PD SP12.
(M) Representative images of Ctrl SP11 and PD SP12 α-syn-FLAG astrocytes stained positive for CD44, GFAP, S100β and negative for TUJ1, MAP2, NG2. Scale bar 100μm.

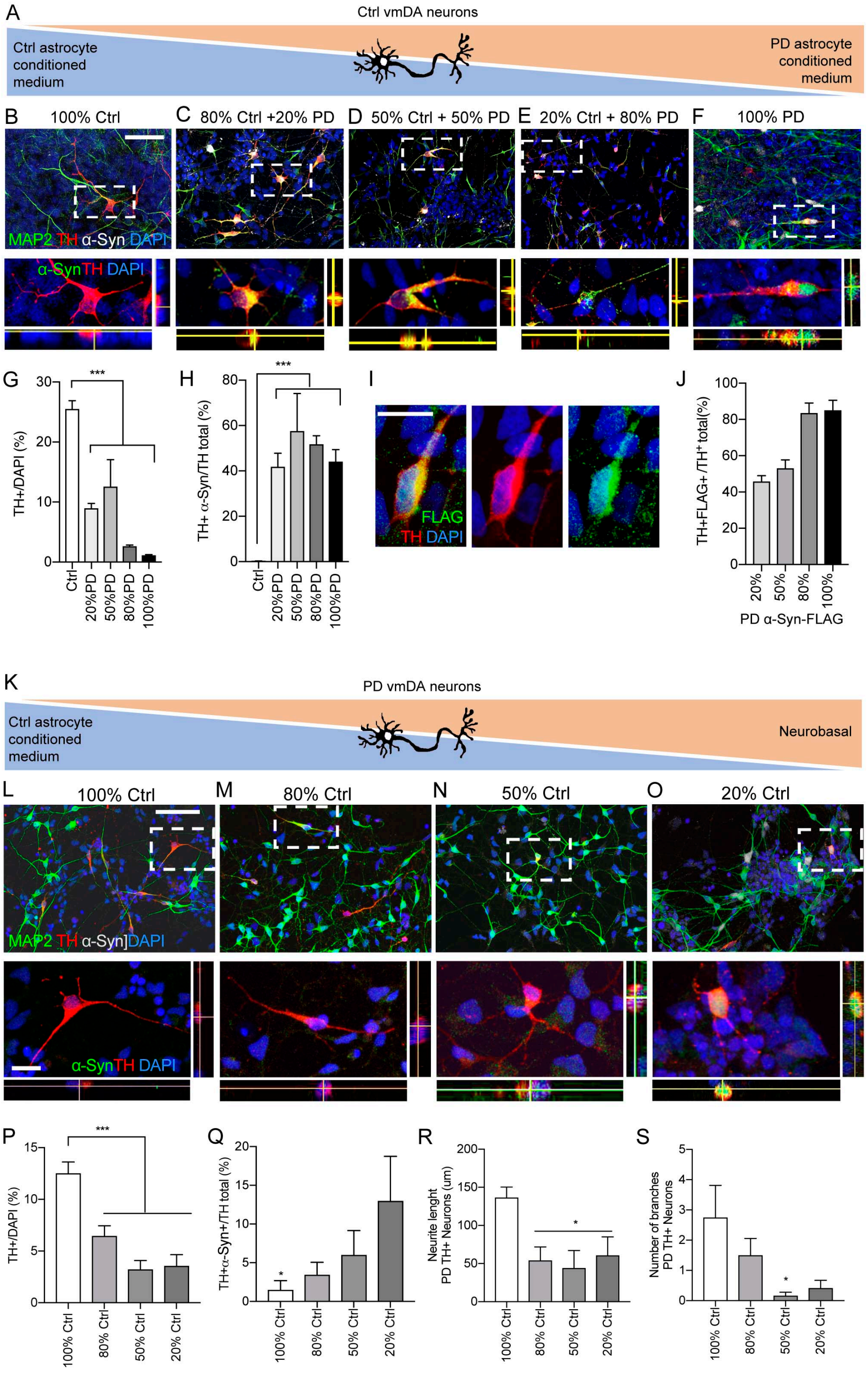


**Figure S3. Effects of Ctrl and PD astrocyte conditioned medium on α-syn accumulation in Ctrl and PD vmDA neurons.**

(A) Diagram representing experimental procedure for astrocyte conditioned medium (indirect contact co-culture) assay.

(B-F) Representative images showing morphology and the α-syn accumulation of Ctrl SP11 vmDAn treated with Ctrl or PD astrocytes-conditioned medium mixed in different proportions for 7 days: 100% Ctrl astrocyte medium (B) 80%Ctrl+20%PD (C), 50%Ctrl+50%PD (D), 20%Ctrl+80%PD (E) and 100%

PD (F) of Ctrl SP09 and PD SP12, respectively. Samples were stained for TH, MAP2, α-syn and DAPI. Scale bar 50μm for top picture and 20μm for all insets.

(G-H) Quantification of vmDAn over all cells (G), and those vmDAn accumulating α-syn (H) in Ctrl neurons treated with PD astrocyte-conditioned medium (n=3).

(I) Representative image showing a FLAG positive Ctrl SP11 neuron cultured with astrocyte-conditioned medium from PD SP12 α-syn -FLAG. Scale bar 20μm.

(J) Quantification of the percentage of Ctrl vmDAn stained positive for FLAG after being treated with astrocyte-conditioned medium from PD SP12 α-syn-FLAG (n=3).

All graphs plot mean ± s.e.m, unpaired two-tailed Student's t-test ***p<0.001.

(K) Diagram representing experimental procedure for astrocyte conditioned medium (indirect contact co-culture) assay.

(L-O) Representative images showing morphology and the α-syn accumulation of PD SP12 vmDAn treated with Ctrl astrocyte-conditioned medium mixed with basic neurobasal (NB) medium at different proportions for 7 days: 100% of Ctrl astrocyte-conditioned medium (L) 80%Ctrl+20%NB (M), 50%Ctrl+50%NB (N), 20%Ctrl+80%NB (O). Samples stained for TH, MAP2, α-syn and DAPI. Scale bar 50μm for top picture and 20μm for all insets.

(P-Q) Quantification of vmDAn over all cells (P), and those vmDAn accumulating α-syn (Q) in PD neurons treated with Ctrl astrocyte-conditioned medium (n=3).

(R-S) Quantification of neurite length (R) and number of branches (S) of PD vmDAn cultured with different concentrations of Ctrl astrocyte-conditioned medium (n=3); total neurons counted per experiment n=10.

All graphs plot mean ± s.e.m, unpaired two-tailed Student's t-test *p<0.05 and ***p<0.001.

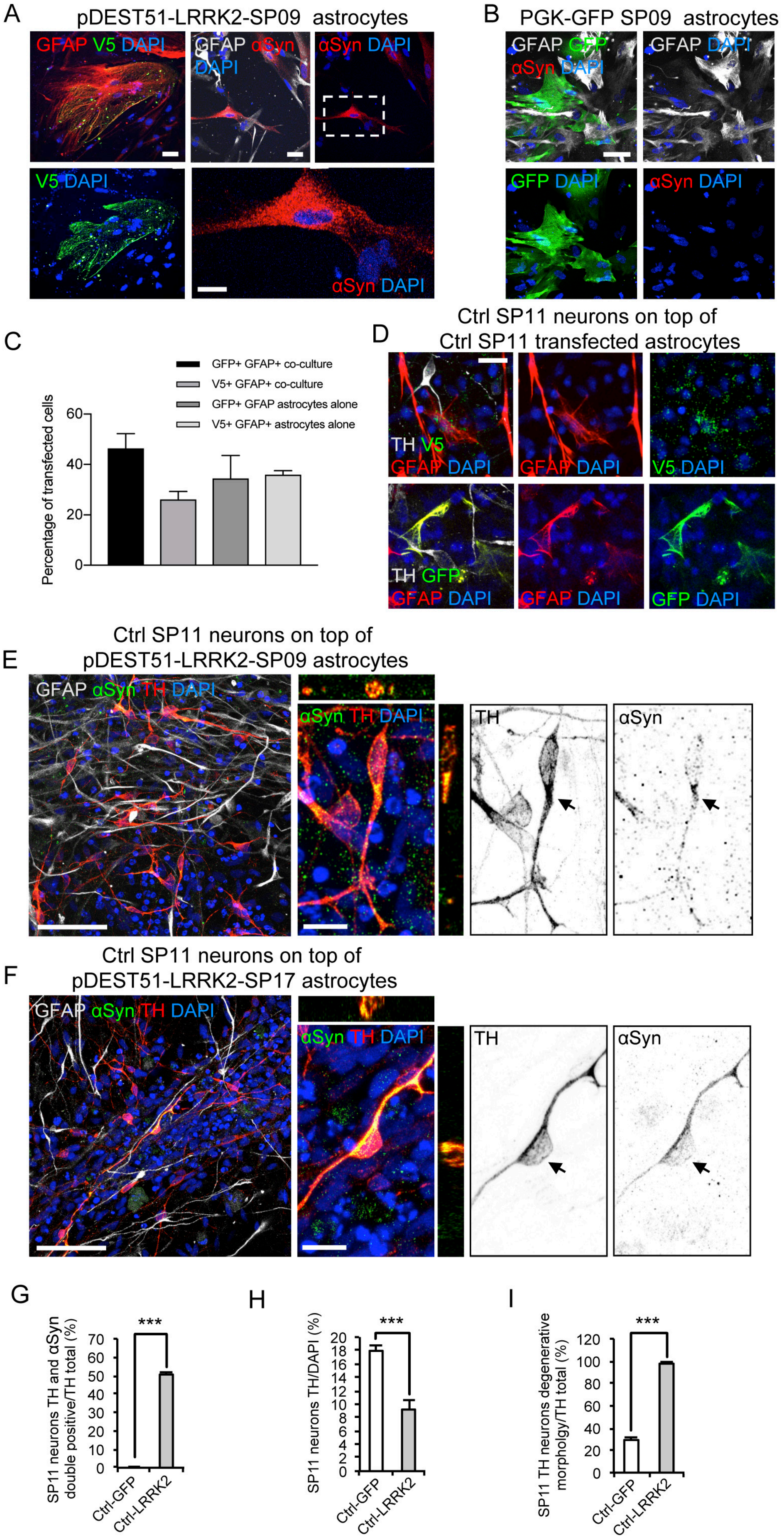

A
pDEST51-LRRK2-SP09 astrocytes
GFAP V5 DAPI
GFAP αSyn DAPI
αSyn DAPI
V5 DAPI
αSyn DAPI
B
PGK-GFP SP09 astrocytes
GFAP GFP αSyn DAPI
GFAP DAPI
GFP DAPI
αSyn DAPI
C
GFP+ GFAP+ co-culture
V5+ GFAP+ co-culture
GFP+ GFAP astrocytes alone
V5+ GFAP+ astrocytes alone
Percentage of transfected cells
60
40
20
0
D
Ctrl SP11 neurons on top of
Ctrl SP11 transfected astrocytes
TH V5 GFAP DAPI
GFAP DAPI
V5 DAPI
TH GFP GFAP DAPI
GFAP DAPI
GFP DAPI
E
Ctrl SP11 neurons on top of
pDEST51-LRRK2-SP09 astrocytes
GFAP αSyn TH DAPI
αSyn TH DAPI
TH
αSyn
F
Ctrl SP11 neurons on top of
pDEST51-LRRK2-SP17 astrocytes
GFAP αSyn TH DAPI
αSyn TH DAPI
TH
αSyn
G
SP11 neurons TH and αSyn double positive/TH total (%)
70
60
50
40
30
20
10
0
***
Ctrl-GFP
Ctrl-LRRK2
H
SP11 neurons TH/DAPI (%)
20
18
16
14
12
10
8
6
4
2
0
***
Ctrl-GFP
Ctrl-LRRK2
I
SP11 TH neurons degenerative morpholgy/TH total (%)
120
100
80
60
40
20
0
***
Ctrl-GFP
Ctrl-LRRK2

**Figure S4**. **Effect of p*LRRK2* G2019S transfected in control astrocytes.**

(A) Representative image of pDEST51-*LRRK2* G2019S-transfected Ctrl SP09 astrocytes stained positive for V5 epitope and showing α-syn accumulation after 14 days. Samples were stained for GFAP, V5 (control of transfection) and DAPI; or GFAP, α-syn and DAPI. Scale bar 50μm and 20μm for inset.

(B) Representative image of a Ctrl SP09 astrocyte stained positive for GFP and negative for α-syn 14 days after transfection with a GFP expression plasmid. Samples were stained for GFAP, GFP (control of transfection), α-syn and DAPI. Scale bar 20μm.

(C) Percentage of *LRRK2* G2019S (V5)- and control (GFP)-transfected astrocytes (Ctrl SP09 and Ctrl SP17) after 4 weeks in co-culture with Ctrl SP11 vmDAn or cultured alone for 2 weeks (n=3).

(D) Representative image of Ctrl SP11 vmDAn co-cultured with Ctrl SP11 GFP- or LRRK2- transfected astrocytes for 4 weeks. Samples were stained for TH, GFAP, V5 and DAPI or TH, GFAP, GFP and DAPI. Scale bar 20μm.

(E-F) Representative image of Ctrl SP11 vmDAn co-cultured with Ctrl SP09 (E) and Ctrl SP17 (F) astrocytes overexpressing *LRRK2* G2019S for 4-weeks. Samples stained for TH, GFAP, α-syn and DAPI. Scale bar, 50μm for large picture and 20μm for all zooms.

(G-I) Quantification of the percentage of Ctrl vmDAn showing α-syn accumulation (G) quantification of the percentage of survival vmDAn (H) and quantification of percentage of vmDAn showing degenerative morphology (I) after 4-week co-culture with GFP- or *LRRK2* G2019S- transfected Ctrl astrocytes (n=3). All graphs plot mean ± s.e.m, unpaired two-tailed Student's t-test, *p<0.05, **p<0.01, ***p<0.001.

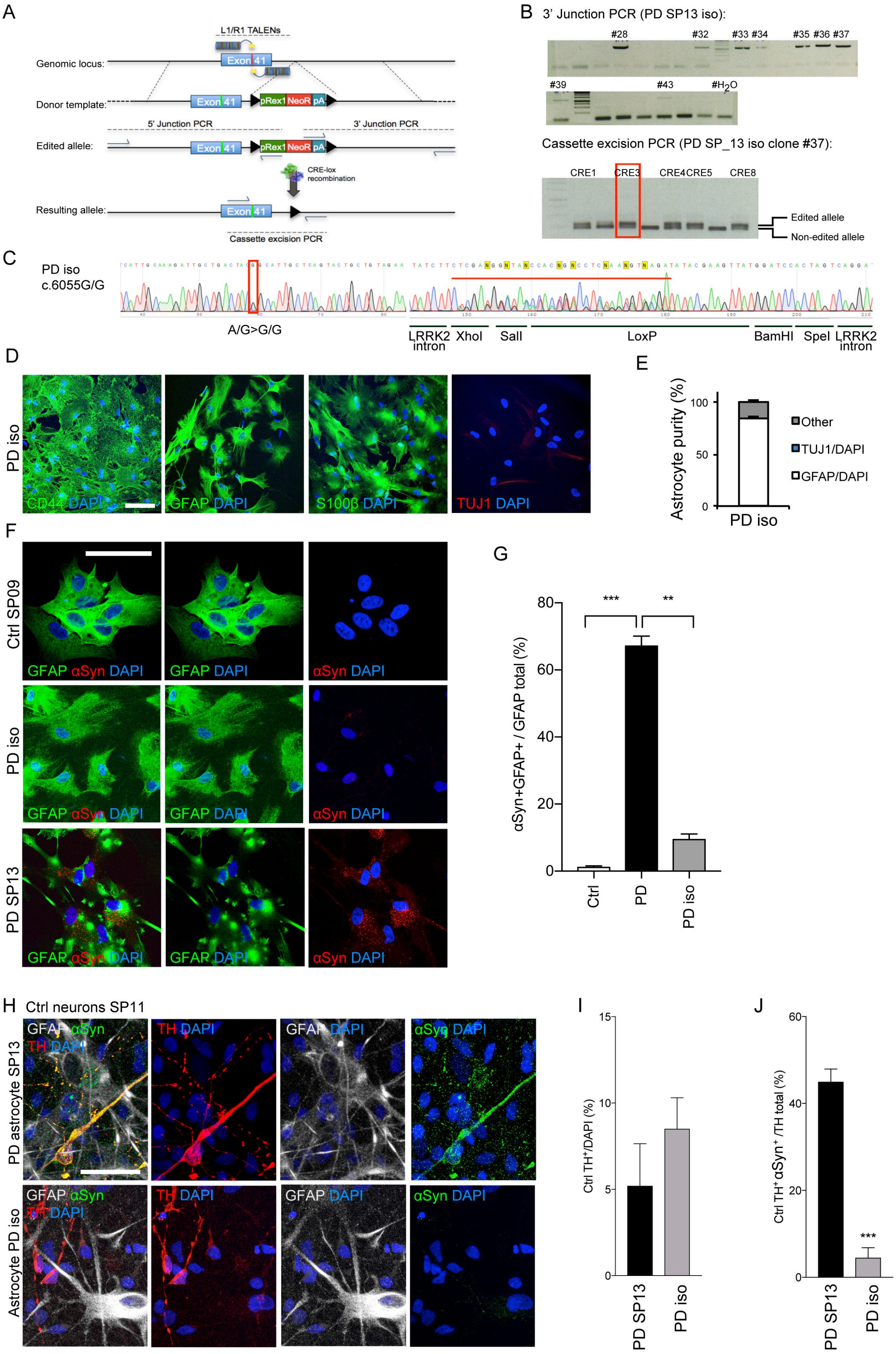
A
L1/R1 TALENs
Genomic locus:
Exon 41
Donor template:
pRex1 NeoR pA
5' Junction PCR
3' Junction PCR
Edited allele:
CRE-lox recombination
Resulting allele:
Cassette excision PCR
B
3' Junction PCR (PD SP13 iso):
#28
#32
#33 #34
#35 #36 #37
#39
#43
#H2O
Cassette excision PCR (PD SP_13 iso clone #37):
CRE1
CRE3
CRE4CRE5
CRE8
Edited allele
Non-edited allele
C
PD iso
c.6055G/G
A/G>G/G
LRRK2 intron
XhoI
SalI
LoxP
BamHI
SpeI
LRRK2 intron
D
PD iso
CD44 DAPI
GFAP DAPI
S100β DAPI
TUJ1 DAPI
E
Astrocyte purity (%)
Other
TUJ1/DAPI
GFAP/DAPI
PD iso
F
Ctrl SP09
PD iso
PD SP13
GFAP αSyn DAPI
GFAP DAPI
αSyn DAPI
G
αSyn+GFAP+ / GFAP total (%)
Ctrl
PD
PD iso
H
Ctrl neurons SP11
PD astrocyte SP13
Astrocyte PD iso
GFAP αSyn TH DAPI
TH DAPI
GFAP DAPI
αSyn DAPI
I
Ctrl TH+/DAPI (%)
PD SP13
PD iso
J
Ctrl TH+ αSyn+ /TH total (%)
PD SP13
PD iso

**Figure S5. Schematic description of the generation of isogenic control line and phenotypic comparison between isogenic clones, alone or in co-culture with Ctrl-SP11 neurons.** (A) Scheme describing the gene editing process resulting in the generation of isogenic *LRRK2* G2019S iPSC line using TALENs. Blue arrows represent the primers used for the PCR screening procedure. Black triangles represent LoxP sites surrounding the selection cassette. Green and red bars in Exon 41 represent the *wild type* and the G2019S alleles respectively.

(B) Molecular analysis of the resistant clones checking proper pRex1-NeoR cassette integration and subsequent cassette excision in PD SP13 iPSC line. In the lower gel, the increase in size of the edited clone is due to the remaining LoxP site. Red rectangles indicate selected #37CRE3 clone.

(C) Sanger sequencing, confirmed both successful excision of the LoxP site-flanked cassette and the successful correction of the mutation.

(D) Representative images of PD iso astrocytes staining positive for CD44, GFAP, S100β and negative for TUJ1. Scale bar 100 μm.

(E) Astrocyte cultures of PD iso line are approximately composed of 84% astrocytes, 1% neurons and 15% other (n=3).

(F) Representative images of Ctrl SP09, PD iso and PD SP13 astrocytes at 14 days stained for GFAP and α-syn. Scale bar 50 μm.

(G) Percentage of PD SP13 and PD iso astrocytes positive for α-syn after 14 days in culture (n=3).

(H) Representative images of Ctrl SP11 vmDAn on top of PD SP13 (upper panel) and PD iso astrocytes (bottom panel). Samples were stained for TH, GFAP, α-syn and DAPI. Scale bar 20 μm.

(I-J) Quantification of the percentage of Ctrl vmDAn remaining after 4-week co-culture with PD SP13 or PD iso astrocytes (I). Percentage of those remaining vmDAn showing α-syn accumulation (J) (n=3).

All graphs plot mean ± s.e.m, unpaired two-tailed Student's t-test, $*p<0.05$, $**p<0.01$, $***p<0.001$.

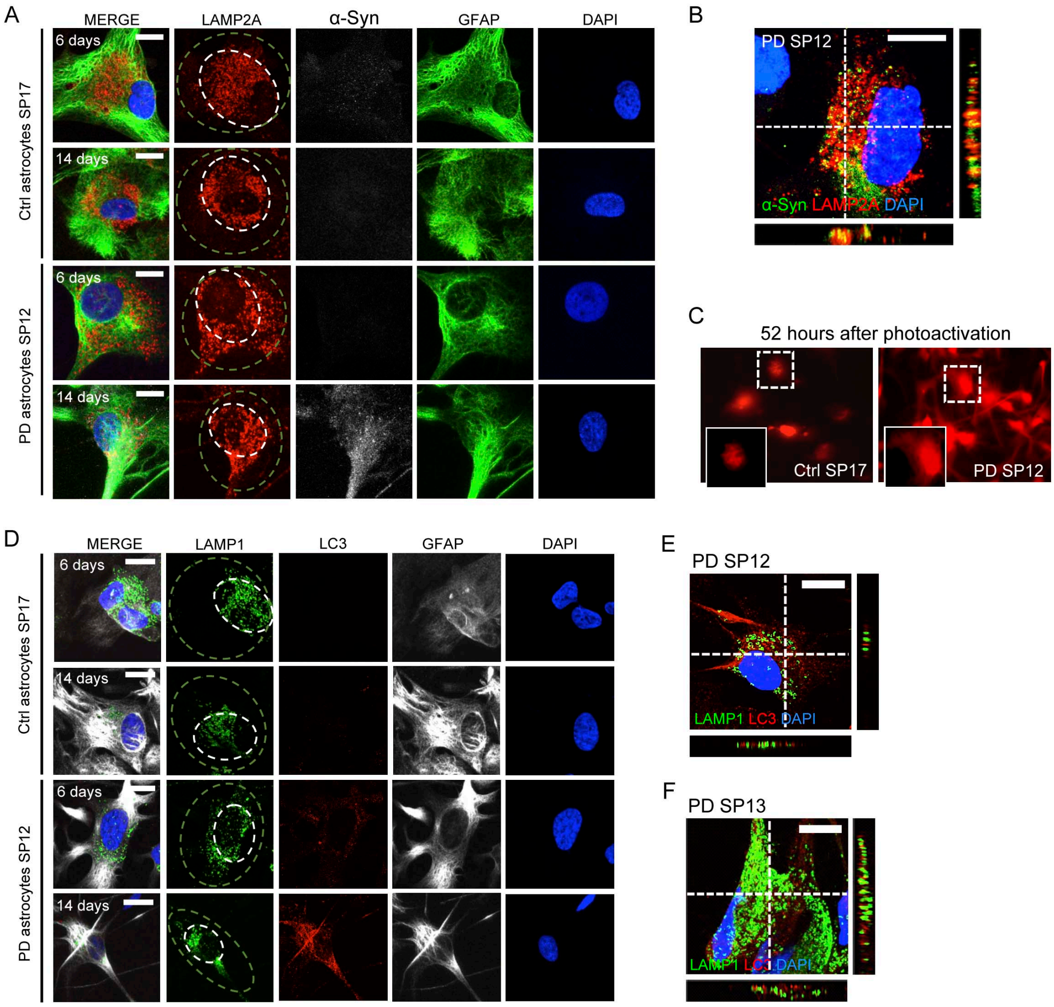


**Figure S6. Altered CMA, dysfunctional macroautophagy and α-syn accumulation in PD astrocytes.** (A) Representative images of Ctrl SP17 and PD SP12 astrocytes stained positive for the LAMP2A, GFAP, α-syn and DAPI after 6 and 14 days in culture. Scale bar 20µm. Smaller white circles represent perinuclear area, whereas larger green circles represent non-perinuclear area.

(B) Positive co-localization between LAMP2A and α-syn in PD SP12 astrocyte line. Scale bar 10µm.

(C) KFERQ-DENDRA (CMA reporter) in Ctrl SP17 and PD SP12 astrocytes 52 hours after photo-switching with UV light (n=3).

(D) Representative images of lysosomal protein marker LAMP1 and autophagosome marker LC3 in Ctrl SP17 and PD SP12 astrocytes (GFAP) at 6 and 14 days. Scale bar 20µm. Smaller white circles represent perinuclear area, whereas larger green circles represent non-perinuclear area (n=3).

(E-F) Lack of co-localization between lysosomes (LAMP1) and autophagosomes (LC3) in astrocyte lines PD SP12 and PD SP13. Scale bar 10µm.

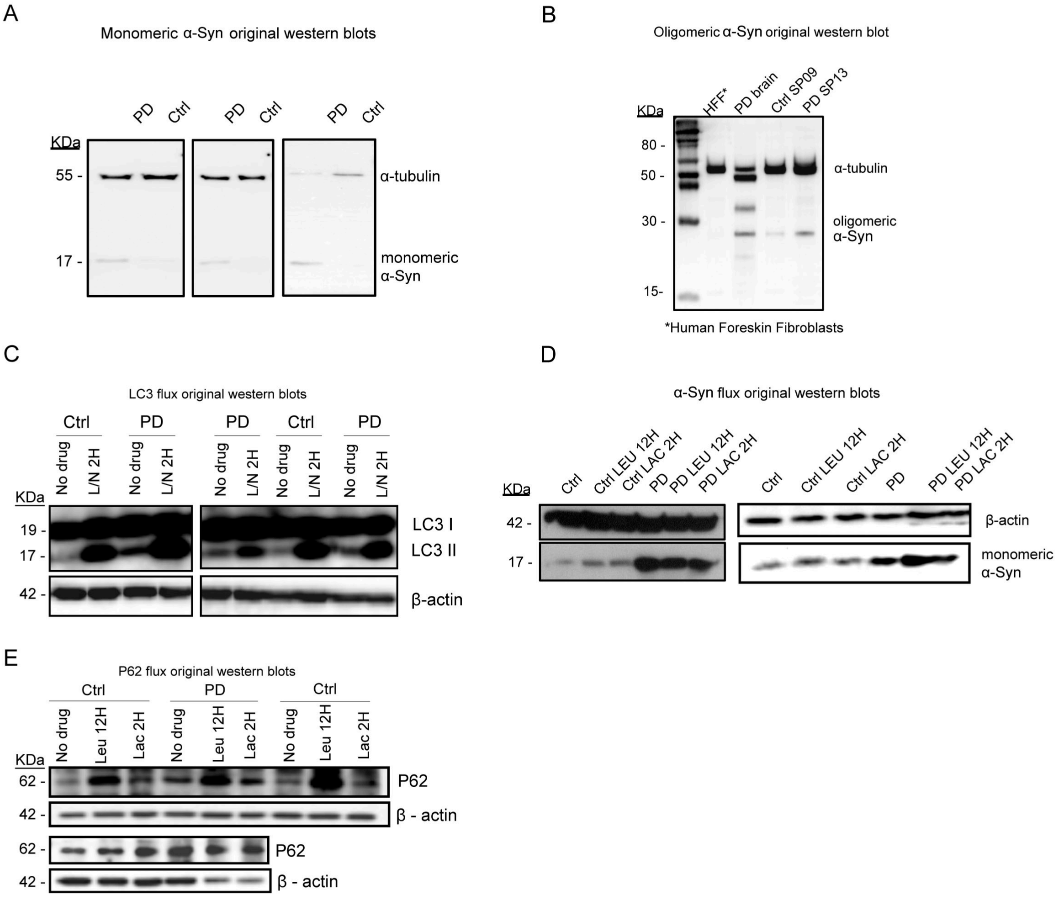


**Figure S7. Original uncropped Western blots.**

(A) Monomeric α-syn protein Western blots.

(B) Oligomeric α-syn protein Western blot.

(C) LC3 flux Western blots.

(D) α-Syn flux Western blots.

(E) p62 flux Western blots.

## SUPPLEMENTAL EXPERIMENTAL PROCEDURES

### iPSC-derived astrocyte generation and culture

The parental iPSC lines used in our studies were previously generated and fully characterized (Sanchez-Danes et al., 2012). Specifically, we used iPSC generated from three patients harboring the G2019S mutation on the *LRRK2* gene (PD SP06, PD SP12, and PD SP13), and from three healthy age-matched controls (Ctrl SP09, Ctrl SP11, and Ctrl SP17). The generation of isogenic control and α-syn-FLAG-tagged iPSC lines is described below. The generation and/or use of human iPSCs in this work were approved by the Spanish competent authorities (Commission on Guarantees concerning the Donation and Use of Human Tissues and Cells of the Carlos III National Institute of Health). All procedures were done in accordance with institutional guidelines and the human iPSC lines have been (or are in the process of being) deposited at the Spanish National Stem Cell Bank, according to the Spanish legislation. iPSC were differentiated into spherical neurospheres containing neuroectodermal progenitors and then differentiated toward an astrocytic lineage following a previously published protocol (Serio et al., 2013). First, the SNMs were grown in suspension for 28 days with Induction Medium (DMEM/F12, 1% N2 supplement, 0.1% B27 supplement (Life, 17504-044), 1% nonessential amino acids (NEAA), 1% penicillin/streptomycin (PenStrep), 1% Glutamax) supplemented with 20 ng/mL LIF (Sigma) and 20 ng/mL EGF (R&D Systems), and then for further 21 days with Propagation Medium (DMEM/F12, 1% N2 supplement, 0.1% B27 supplement, 1% NEAA, 1% PenStrep, 1% Glutamax) containing 20 ng/mL FGF-2 (PeproTech) and 20 ng/mL EGF (R&D Systems). Finally, SNMs were incubated with accutase (LabClinics) for 15 minutes at 37ºC, mechanically dissaggregated and plated on matrigel-coated plates as a monolayer. The monolayer of neural progenitors was cultured for 14 more days in Propagation Medium and then for 14 more days in CNTF medium (Neurobasal, 1% Glutamax, 1% PenStrep, 1% NEAA, 0.2% B27 supplement, 10 ng/mL CNTF (Prospec Cyt-272), a stage in which they were considered astrocyte progenitors and therefore characterized. These astrocyte progenitors were successfully frozen in Astrocyte Freezing Medium (90% FBS and 10% DMSO) and stored in liquid nitrogen for future use. When needed for an experiment, vials were thawed in medium containing FBS, resuspended in CNTF medium and plated on matrigel-coated plates. Cells were passaged four times before considered mature and then further characterized. Experiments were performed with astrocytes growing on Thermanox™ plastic coverslips (Thermofisher) coated with matrigel in 24-well plates.

### iPSC-derived vmDAn generation

Four different iPSC, two PD (SP12 and SP13) and two Ctrl (SP11 and SP11#4) were differentiated into dopaminergic neurons using a combination of two previously published protocols for midbrain induction (Chambers et al., 2009; Kriks et al., 2011). Briefly, iPSC were maintained in conditioned HES medium for 5 days until they reached 70% of confluence, and were then cultured in SRM medium (day 0) (KO-DMEM, 15% KO serum, 1% NEAA, 1% GlutaMax and 1% P/S) for additional 5 days. After that, iPSCs were grown in Neurobasal medium, 1% N2, 2% B27 without vitamin A and 1% P/S. At day 12, N2 was removed from the medium until the end of the differentiation. Media were supplemented with SB-431542 (10 mM; day 0–day 5; Sigma), LDN193189 (100 nM; day 0–day 12; Miltenyi), CHIR99021 (3 mM; day 3–day 25; Miltenyi), purmorphamine (2 mM; day 1–day 5; Stemgent, Cambridge, Massachusetts,

www.stemgent.com), Smoothened Agonist (SAG) (1 mM; day 1–day 5; Tocris, Bristol, United Kingdom, www.tocris.com), Brain-Derived Neurotropic Factor (BDNF) (20 ng/ml; from day 12; Miltenyi), Glial Cell-Derived Neurotropic Factor (GDNF) (20 ng/ml; from day 12; Miltenyi), DAPT (10 mM; from day 12; Tocris), db-cAMP (500 mM; from day 12; Sigma, St. Louis, Missouri, www.sigmaaldrich.com), TGFB3 (1 ng/ml; from day 12; Miltenyi) and ascorbic acid (AA, 200 mM; from day 12; Sigma). On day twenty, cells were dissociated using Accutase (Merck, Kenilworth, New Jersey, www.merck.com), re-plated at 0,75 x $10^5$ cells per $cm^2$ on dishes pre-coated with polyornithine (15 μg/ml), laminin (1 μg/ml) and fibronectin (2 μg/ml) and cultured in Neurobasal medium with 2% B27 without vitamin A and 1% P/S with trophic factors (GDNF, BDNF, TGFB3, AA, cAMP and DAPT) until analysis. Quantification of neurons (at day 35 and 50) was assessed by confocal microscopy using anti-FOXA2, anti-MAP2, anti-TH, anti-GIRK2 and anti-DAT antibodies. After 35 days of differentiation 20% of overall cells stained positive for TH, a number which increased with time reaching 35% after 50 days, a point in which the neurons were considered as mature.

**Gene Editing**

<u>TALEN, CRISPR guideRNA (gRNA) and Donor Plasmid Design.</u> TALEN monomers were engineered as described elsewhere (Mussolino et al., 2011) in the Institute for Cell and Gene Therapy & Center for Chronic Immunodeficiency (University of Freiburg). They were composed of 19 RVDs and were fused to wild type FokI nuclease domains. Repeats containing the NN RVD were used for Guanidine recognition. Each monomer was inserted in a plasmid under the control of a modified CMV promoter (Alwin et al., 2005). Different TALEN monomers recognizing DNA motifs adjacent to the genomic G2019S mutation site were designed. The TALEN combination showing the highest cleavage efficiency in the T7EI assay was chosen for gene correction. CRISPR/Cas9 gRNAs targeting the last exon-3'UTR junction of the human *SNCA* gene were designed so that the spacer sequence overlapped the STOP codon. Complementary oligos encoding for the desired spacer sequences were annealed and ligated into the BbsI site of the Cas9/guideRNA co-expression plasmid px458 (Addgene #48138). In order to increase Cas9 expression in hPSC, CBh promoter was replaced by the full-length CAGGS promoter (pCAGGS-458). The gRNA with the highest cleavage efficiency in the T7EI assay was selected for the editing procedure. Donor templates for HDR were generated using standard molecular cloning procedures. Briefly, for *LRRK2* donor template, homology arms (HAs) spanning approximately 800 bp from a position 34 bp downstream *LRRK2* exon 42 were amplified from genomic DNA from either wild-type or *LRRK2* G2019S mutant hiPSC. A floxed selection cassette was placed in between the HAs. The selection cassette, pRex1-NeoR-SV40pA, was amplified from aMHC-eGFP-Rex-Neo (Kita-Matsuo et al., 2009) (Addgene; #21229) with primers containing the LoxP sites in the proper orientation.

Donor plasmid for knocking-in a FLAG tag fused C-terminal to the α-syn open reading frame (ORF) was engineered using the following elements. Two homology arms (HAs) spanning approximately 800 bp from both sides of the STOP codon. The sequence encoding for the FLAG-tag was placed right after the last codon of the *SNCA* ORF and before the STOP codon. A selection cassette (pRex1-NeoR) flanked by loxP sites was cloned between the STOP codon and the 3'HA.

The primers employed during the cloning procedure were: SNCA_gRNA2_OL-F 5'-TGGGAGCAAAGATATTTCTT-3', SNCA_gRNA2_OL-R 5'- AAGAAATATCTTTGCTCCCA-3' for cloning the guideRNA spacer sequence into the pCAGGS-458 plasmid; LRRK2_5-F_KpnI 5'- AAG GTA CCC CTT AAT ATC TAA CAT GAT TAG G-3', LRRK2_5-R_XhoI 5'- AAC TCG AGA AGA TAG AAT TAT GAG ACA GAC-3', Rex1_F-loxP-SalI 5'- AAG TCG ACA TAA CTT CGT ATA GCA TAC ATT ATA CGA AGT TAT GAC CGA TTC CTC CCG ATA AG-3', Neo_R-loxP-BamHI 5'- AA GGATCC ATAACTTCGTATA ATGTATGC TATACGAAGTTAT TAAGATACATTGATGAGTTTGGA-3', LRRK2_3-F_SpeI 5'- AAA CTA GTC AGG ATG GAT AAC CAC TGA C-3', LRRK2_3-R_NotI 5'- AAG CGG CCG CTC CCT AAA GAT AGA GTG TTC C-3' For LRRK G2019S correction;

and SNCA_5-F_XhoI 5'- AACTCGAGACTCAAGCTTAGGAACAAGGA-3', SNCA_5FLAG-R_SalI 5'-AAGTCGACATAACTTCGTATAGCATACATTATACGAAGTTATGACCGATTCCTCCCGATAAG-3', Rex1_F-loxP-SalI 5'-AAGTCGACATAACTTCGTATAGCATACATTATACGAAGTTATGACCGATTCCTCCCGATAAG-3', Neo_R-loxP-BamHI 5'-AAGGATCCATAACTTCGTATAGCATACATTATACGAAGTTATTAAGATACATTGATGAGTTTGGA-3', SNCA_3-F_BamHI 5'-AAGGATCCGAAATATCTTTGCTCCCAGT-3', SNCA_3-R_NotI 5'-AAGCGGCCGCTTAAGGAACCAGTGCATAC-3' for SNCA-FLAG donor plasmid construction.

CRISPR-mediated *SNCA* locus edition in hiPSC. The day before transfection, 800.000 Ctrl SP11 and PD SP12 iPSC lines were seeded in a 10cm plate coated with matrigel. The following day, hiPSC were co-transfected with a mix of 6 µg of Cas9-T2A-EGFP/gRNA, 9 µg of the donor plasmid, 45 µL of FuGENE HD (Promega) transfection reagent and KO-DMEM up to 750 µL. The transfection mixture was incubated for 15 minutes at RT and subsequently added to the cells dropwise. 50 µg/mL Geneticin (G-418; Melford Labs) selection was initiated 72h post-transfection and was maintained until the emerging colonies were transferred to another plate. Between 10 and 14 days after the initiation of the selection, colonies were large enough as to be screened. Half of the colony was sampled in order to check site-specific integration by means of PCR. Those colonies that were positive for the targeted recombination were transferred to a different well in order to be transfected with a CRE-recombinase expression plasmid. After CRE transfection, cells were singularized and seeded at a low density on top of an irradiated human fibroblast feeder layer in the presence of ROCK inhibitor (Miltenyi). Once, the colonies attained a certain size, they were isolated and screened for the excision of the selection cassette. Those clones whose both *SNCA* alleles were tagged with the FLAG epitope were expanded and characterized in terms of pluripotency and genome stability. The primers used for the screening procedure and the molecular characterization; SNCA_5Out_F (referred as 5'Out in **Fig. S4**) 5'-CTCACACAGACACACGAAAGG-3', FLAG_R 5'-AGCACCGAAATGCTGAGTG-3', Check_Rex1Neo_3HA_F 5'-CCCGTCTGTTGTGTGACTC-3', SNCA_3Out_R (referred as 3'Out in **Fig. S4**) 5'-ACGTAAAGCAAACATTGACAGG-3', SNCA_T7_F (referred as 5'In in **Fig. S4**) 5'-TGCATCCGGATCAGAACCTA-3', SNCA_T7_R (referred as 3'In in **Fig. S4**) 5'-AGCACCGAAATGCTGAGTG-3'.

TALEN-mediated LRRK2 G2019S correction in hiPSC. The day of transfection, cells were detached from a confluent 10-cm plate and were electroporated as small clumps with 15 ug of each TALEN monomer and 30 ug of a plasmid donor template. This plasmid contained two arms of homology (the left one bearing the wild type allele) placed at both sides of a floxed pRex1-NeoR-SV40pA selection cassette. The primers used for the screening procedure and the molecular characterization; LRRK2_Out_5'HA_F 5'- TCGTGATTGCGTGGGTC-3', LRRK2_Out_3'HA 5'-GCAGGAAACGAACTAGAACC -3', Check_Rex1Neo_5HA_R 5'-CTTATCGGGAGGAATCGGTC-3', Check_Rex1Neo_3HA_F 5'-CCCGTCTGTTGTGTGACTC-3', LRRK2_T7_F 5'-GGGACAAAGTGAGCACAG-3', LRRK2_T7_Sel_R 5'- CACAAGTGCCAACAATACC-3'.

**Astrocytes and vmDA co-culture**

$2x10^4$ astrocytes were plated per well in 24-well plates pre-coated with matrigel and let to generate a confluent monolayer. One week after, vmDAn (35-day old) were plated onto the astrocyte monolayer at $5x10^4$ cells per well. Co-cultures were carried out in Neurobasal medium, containing 1% PenStrep, 2% B27 supplement minus Vitamin A (ThermoFisher, 12587001). In addition, we tested the effect of the vmDAn medium in astrocytes alone without noticing any differences in astrocyte survival (data not shown). Cells were fixed and stained for the markers indicated in each experiment. For assessing vmDA neuron survival, the abundance of TH+ cells was counted using FIJI is Just ImageJ™ cell counter plugin.

**Astrocyte conditioned medium (indirect co-culture)**

$3x10^5$ astrocytes were plated per well on a matrigel coated 6-well plate in 2mL of CNTF medium. Each line was cultured for 14 days without changing the initial medium. At day 6, 1mL of fresh CNTF medium was added to each well. After the 14-day time-point, the medium was collected and frozen at -80ºC. Different ratio of Ctrl SP09 and PD SP12 astrocyte-conditioned medium was mixed (Ctrl and PD%: 100, 80/20, 50/50, 20/80, 100) to obtain medium to treat Ctrl SP11 neurons. Using the same paradigm, Ctrl SP11 neurons were treated with different ratio of Ctrl SP09 and PD SP12 α-syn-FLAG mixed medium (100, 80/20, 50/50, 20/80) to verify the effective transfer (contact independent) of the α-syn from the astrocyte conditioned medium to neurons. In contrast, to treat PD SP12 neurons, we only mixed Ctrl SP09 astrocyte-conditioned medium with Neurobasal Medium (% respectively, 100, 80/20, 50/50, 20/80).

**Immunocytochemistry**

Samples were fixed using 4% PFA for 15 minutes and then washed three times for 15 minutes with PBS. Samples were blocked and permeabilized with TBS++ with low triton (TBS, 3% Normal Donkey Serum, 0.01% Triton X-100) for 2 hours and subsequently incubated with the primary antibody for 48 hours at 4ºC. Primary antibodies used include mouse anti- CD44 (Abcam, ab6124), guinea pig anti-GFAP (Synaptic Systems, 173 004), rabbit anti-GFAP (Dako, Z0334), rabbit anti-S100β (Dako, 311), mouse anti-Vimentin IgM (Iowa, 3CB2), mouse anti-TUJ1 (Covance, MMS-435P), rabbit anti-MAP2 (Sant Cruz, sc-20172), rabbit anti-NG2 (Millipore, AB5320), guinea pig anti-GLT-1 (Millipore, AB1783), rabbit anti-Synapsin-I (Calbiochem, 574777), rabbit anti-LC3B (Cell Signaling, 2775), rabbit anti-LAMP-2A (Abcam, 18528), mouse anti-FLAG-M2 (Sigma, F3165), mouse α-syn (BD, 610787), mouse anti-α-syn (Agisera AS13 2718), sheep anti-TH (Pel-Freez, P60101-0), rabbit anti-TH (Santa Cruz, sc-

14007), mouse anti-LAMP1 (Iowa, H4A3). Samples were then washed with TBS 1x for 15 minutes three times, and blocked again for one hour at RT. Samples were incubated with secondary antibodies (1:200) for 2 hours at RT: Alexa Fluor 488 anti-Mouse IgG (Jackson 715-545-150), Cy3 anti-rabbit IgG (Jackson 711-165-152), DyLight 649 anti-Guinea pig IgG (Jackson 706-495-148), Alexa Fluor 647 anti-Sheep (Jackson 713-605-147), Cy™2 AffiniPure Donkey Anti-Rabbit IgG (H+L) (Jackson 711-225-152), Cy™3 AffiniPure Donkey Anti-Mouse IgG (H+L) (Jackson 715-165-151). Samples were then washed with TBS 1x for 15 minutes three times, incubated with nuclear staining DAPI (Invitrogen, 1:5000) for 10 minutes, mounted with PVA:DABCO and stored at 4ºC until imaged. Samples were imaged using an SP5 confocal microscope (Leica) and analyzed with FIJI is Just ImageJ™.

**RNA extraction and gene expression analysis**

The isolation of total mRNA was performed with the RNeasy Micro Kit and treated with RNase free DNase I (Qiagen). 500ng were used to synthesize cDNA with the SuperScript III Reverse Transcriptase Synthesis Kit (Invitrogen). Quantitative RT-PCR analyzes were done in triplicate using 2ng/ul cDNA with Platinum SYBR Green qPCR Super Mix (Invitrogen) in an ABI Prism 7000 thermocycler (Applied Biosystems). All results were normalized to β-actin.

**Stranded mRNA library preparation and sequencing**

Total RNA was assayed for quantity and quality using Qubit® RNA HS Assay (Life Technologies) and RNA 6000 Nano Assay on a Bioanalyzer 2100. The RNASeq libraries were prepared from total RNA using the TruSeq®Stranded mRNA LT Sample Prep Kit (Illumina Inc., Rev.E, October 2013). Briefly, 500ng of total RNA was used as the input material and was enriched for the mRNA fraction using oligo-dT magnetic beads. The mRNA was fragmented in the presence of divalent metal cations and at high temperature (resulting RNA fragment size was 80-250 nt, with the major peak at 130nt). The second strand cDNA synthesis was performed in the presence of dUTP instead of dTTP, this allowed to achieve the strand specificity. The blunt-ended double stranded cDNA was 3′adenylated and Illumina indexed adapters were ligated. The ligation product was enriched with 15 PCR cycles and the final library was validated on an Agilent 2100 Bioanalyzer with the DNA 7500 assay. The libraries were sequenced on HiSeq2000 (Illumina, Inc) in paired-end mode with a read length of 2x76 bp using TruSeq SBS Kit v4. We generated over 30 million paired-end reads for each sample in a fraction of a sequencing v4 flow cell lane, following the manufacturer's protocol. Image analysis, base calling and quality scoring of the run were processed using the manufacturer's software Real Time Analysis (RTA 1.18.66.3) and followed by generation of FASTQ sequence files by CASAVA. The RNA-seq data have been deposited in Gene Expression Omnibus (GEO) of the National Center for Biotechnology Information and are accessible through GEO Series accession number GSE116124 (www.ncbi.nlm.nih.gov/geo/query/acc.cgi?acc=GSE116124).

**Bioinformatics of RNA sequencing**

RNA-seq paired-end reads were mapped against the human reference genome (GRCh38_primary) using STAR version 2.5.3a (Dobin et al., 2013) with ENCODE parameters for long RNA. Annotated gene and

isoforms (gencode version 27) were quantified using RSEM version 1.3.0 with default parameters (Li and Dewey, 2011). Differential expression analysis was performed with DESeq2 version 1.10.1 (Love et al., 2014). Heatmaps were performed with the ‘pheatmap’ R package with Euclidean distances comparing hiPSC-astrocytes (Ctrl SP09 and PD SP12) to human cortical astrocytes and iPSC lines (Ctrl SP09 and PD SP12).

**ATP production assay**

ATP production was measured using the ATP Determination Kit (A22066, Molecular Probes), using a recombinant firefly luciferase and its substrate D-Luciferin. Each astrocyte line was tested by plating 2x$10^4$ cells per well of a 24-well plate. Cells were washed twice with ice-cold PBS. The cells were scraped with 100 µL of ATP buffer (100 nM Tris-HCl pH 7.75, 4 nmM EDTA), collected and flash frozen in liquid nitrogen, boilt for 3 minutes and kept on ice for 5 min. Samples were then centrifuged at 4ºC for 5 min at 13,000 rpm. The ATP content in the supernatant was measured with the ATP determination kit. Each reaction contained 1.25 µg/mL of firefly luciferase, 50 µM D-luciferin and 1 mM DTT in 1X Reaction Buffer. After a 15-min incubation, luminescence was measured (arbitrary units).

**Calcium imaging**

2x$10^4$ astrocytes were plated per well in 24-well plates. Live astrocytes at passage 4 were incubated with Fluro4-AM flurofore for 30 minutes slowly shaking at RT. Astrocytes were then imaged during 20 minutes using the Hokawo program. Recorded data was converted from video to images. The data was loaded into NeuroImage software where Calcium Activity Map and individual Calcium Graphs were generated. Data is further analyzed in a Matlab code made by Dr. Jordi Soriano laboratory.

**Cell viability assay**

Calcein Green AM (Thermofisher C3100MP) (1µM) and Propidium iodide (Sigma P4170) (1 mg/mL) were added to cells resuspended in 0.5 mL $Ca^{2+}$/$Mg^{2+}$-free PBS supplemented with 2%FBS. Flow cytometry analysis was performed on a Gallios flow cytometer using a 488nm laser for excitation and 525/40 nm and at 575/30 nm emission filters for recording Calcein Green AM and Propidium Iodide signal respectively. Interpretation of cytometry data was done using Kaluza Software (Beckman Coulter Inc, Brea, CA).

**CMA Activator (CA) treatment**

We used a CMA activator (CA) that operates through the release of the endogenous inhibition of the retinoic receptor-α signaling pathway over CMA (Anguiano et al., 2013). Astrocytes (PD SP13) were treated with 20µM CA for 4 days (conditions inducing maximal activity and no toxicity of CA as evaluated in preliminary dose-response analyses using mouse fibroblasts), then fixed and analyzed for α-syn accumulation. Co-cultures of Ctrl SP11 neurons on PD astrocytes (SP13 and SP12) started CA treatment from the 2nd week and it was added daily until the 4th week when cells were fixed, stained and analyzed for neuronal survival and α-syn accumulation within DA neurons and astrocytes.

**Protein extraction**

For pellet collection, $3x10^5$ cells per well of a matrigel-coated 6-well plate were plated and each well equated to one pellet. Live cells were washed twice with PBS and incubated for 6 minutes at 37ºC with accutase (Sigma). Cells were lifted and collected in washing medium containing FBS and centrifuged at 800rpm for 5 minutes. After centrifugation, cells were resuspended in cold PBS and centrifuged for 5 minutes at 4ºC at 600xg. Pellets were immediately stored at -80ºC for future use. For protein extraction, pellets were homogenized in 50mM Tris-HCl, pH 7.4/150 mM NaCl/0.5% Triton X-100/0.5% Nonidet P-40 and a mixture of proteinase inhibitors (Sigma, Roche tablet). Samples were then centrifuged at 15,000xg for 20 minutes at 4ºC. The resulting supernatant was normalized for protein using BCA kit (Pierce). For blotting oligomeric forms of α-syn, cells were lysed in Mila lysis buffer (0.5M Tris at pH 7.4 containing 0.5 methylenediaminetetraacetic acid at pH 8.0, 5M NaCl, 0.5% Na doxicholic, 0.5% Nonidet P-40, 1mM phenylmethylsulfonyl fluoride, bi-distilled water, protease and phosphatase inhibitor cocktails) (Roche Molecular Systems, Pleasanton, CA, USA), and then centrifuged for 15 min at 13,000rpm at 4°C (Ultracentrifuge Beckman with 70Ti rotor, CA, USA).

**Western blot (WB)**

Cell extracts were boiled at 100ºC for 5 minutes, followed by 7-15% SDS-PAGE, electrotransferred to PVDF membranes for 1.5 hours at 4ºC and blocked with 5% not-fat milk in 0.1M Tris-buffered saline (pH= 7.4) for 1 hour. Membranes were incubated O/N at 4ºC with primary antibodies diluted in TBS/3% BSA/0.1% TWEEN. After incubation with peroxidase-tagged secondary antibodies (1:10,000), membranes were revealed with ECL-plus chemiluminescence western blot kit (Amershan-Pharmacia Biotech). The following antibodies were used: mouse anti-α-syn (BD, 610787), rabbit anti-LAMP-2A (Abcam, 18528), rabbit anti-LC3B (Cell Signaling, 2775), rabbit anti-p62 (Enzo Life Science, BML-PW9860), mouse anti-β-actin (Millipore) and rabbit anti-α-tubulin Millipore). Films were scanned at 2,400 x 2,400 dpi (i800 MICROTEK high quality film scanner), and the densitometric analysis was performed using FIJI is Just ImageJ™. Other membranes were imaged using the ChemiTouch machine under the 'Optimal exposure' setting.

**KFERQ-DENDRA CMA reporter**

CMA activity was measured using a photo-switchable CMA fluorescent reporter with a CMA targeting motif fused N-terminally to PS-Dendra protein (KFERQ-PS-Dendra) (Koga et al., 2011; Park et al., 2015). $2x10^4$ astrocytes seeded in wells of 24-well plates were transduced after 6 days in culture. Three days later, cells were photo-switched with UV light for 3 minutes and then imaged after 52 hours to monitor CMA activity.

**CMA knockdown (shLAMP2A)**

$2x10^4$ astrocytes seeded in wells of 24-well plates were transduced after 12 days in culture with LV-shLAMP2A (Massey et al., 2008) or LV-shLuciferase as a control. Three days later, cells were fixed and stained for α-syn. The cellular area occupied by α-syn puncta was measured using a macro developed in FIJI is Just ImageJ™.

**LC3 flux assay**

$3x10^5$ astrocytes seeded in wells of 6-well plates were treated after 14 days in culture with 100 μM leupeptin (Sigma L2884) and 20 mM $NH_4Cl$ (Sigma A9434) for 2 hours in order to stop lysosomal proteolysis. The pellets were collected and protein extracts were electrophoresed in 13% SDS-PAGE gels. LC3-II flux was calculated as the difference between 2-hour drug treatment versus untreated.

**α-Syn flux assay**

$3x10^5$ astrocytes seeded in MW6 wells were treated after 14 days in culture with either 100 μM leupeptin (Sigma L2884) for 12 hours or with the proteoseomal inhibitor lactacystin (5mM, Enzo BML-PI104) for 2 hours. The pellets were collected and protein extracts were electrophoresed in 12.5% SDS-PAGE.

***LRRK2* G2019S overexpression**

$2x10^4$ astrocytes seeded in 24-well plates were co-transfected after 7 days in culture with 1 μg pDEST51-LRRK2-G2019S, which was a gift from Mark Cookson (Addgene plasmid # 29401) and 0.25ug of GFP expression plasmid as transfection control. Transfection was done using Lipofectamine Stem Reagent (Invitrogen) following manufacturer's instructions. Cells were then used for co-culture or fixed and analyzed after one week from the transfection. Cells were stained with an anti-V5-tag or anti-GFP antibody in order to estimate the efficiency of transfection both of astrocytes growing alone or when co-cultured with neurons. Astrocytes used for co-culture had the medium changed the day after the transfection and then during the neuronal plating. Ctrl SP11 vmDA neurons at day 35 were plated on top of the transfected astrocytes and maintained in culture following the previously described protocol for 4 weeks.

**Statistical analysis**

Statistical analyses of the obtained data were performed using two-tailed unequal variance Student *t*-tests and ANOVA (* $p<0.05$, ** $p<0.01$, *** $p<0.001$) and the mean and standard error of the mean were plotted using Prism (Mac OS X). Number of independent experiments (n) is indicated in each figure legend.
