# Supplementary material for: Patient-Specific iPSC-Derived Astrocytes Contribute to Non-Cell-Autonomous Neurodegeneration in Parkinson's Disease
Source: Stem Cell Reports. 2019 Jan 10;12(2):213–29. doi: 10.1016/j.stemcr.2018.12.011 (PMC6372974; doi:10.1016/j.stemcr.2018.12.011)
Supplement: Document S2. Article plus Supplemental Information [file mmc2.pdf]

Stem Cell Reports
Article
OPEN ACCESS

Patient-Specific iPSC-Derived Astrocytes Contribute to Non-Cell-Autonomous Neurodegeneration in Parkinson's Disease

Angelique di Domenico,[1,2,14] Giulia Carola,[1,2,14] Carles Calatayud,[1,2,3] Meritxell Pons-Espinal,[1,2] Juan Pablo Muñoz,[4] Yvonne Richaud-Patin,[3,5] Irene Fernandez-Carasa,[1,2] Marta Gut,[6] Armida Faella,[1,2] Janani Parameswaran,[1,2] Jordi Soriano,[7,8] Isidro Ferrer,[2,9] Eduardo Tolosa,[9,10] Antonio Zorzano,[4] Ana Maria Cuervo,[11] Angel Raya,[3,5,12,15,*] and Antonella Consiglio[1,2,13,15,*]

[1]Department of Pathology and Experimental Therapeutics, Bellvitge University Hospital-IDIBELL, Hospitalet de Llobregat, Barcelona 08908, Spain
[2]Institute of Biomedicine of the University of Barcelona (IBUB), Barcelona 08028, Spain
[3]Center of Regenerative Medicine in Barcelona (CMRB), Hospital Duran i Reynals, Hospitalet de Llobregat, Barcelona 08908, Spain
[4]Institute for Research in Biomedicine (IRB), Carrer Baldiri Reixac 10, Barcelona 08028, Spain
[5]Centre for Networked Biomedical Research on Bioengineering, Biomaterials, and Nanomedicine (CIBER-BBN), Madrid 28029, Spain
[6]Centre Nacional d'Anàlisi Genòmica (CNAG-CRG), Parc Científic de Barcelona, Barcelona 08028, Spain
[7]Departament de Física de la Matèria Condensada, Universitat de Barcelona, Barcelona 08028, Spain
[8]Universitat de Barcelona Institute of Complex Systems (UBICS), Barcelona 08028, Spain
[9]Centre for Networked Biomedical Research on Neurodegenerative Diseases (CIBERNED), Madrid 28049, Spain
[10]Department of Neurology, Hospital Clinic de Barcelona, Institut d'Investigacions Biomédiques August Pi i Sunyer (IDIBAPS), University of Barcelona (UB), Barcelona 08036, Spain
[11]Albert Einstein College of Medicine, Bronx, NY 10461, USA
[12]Institució Catalana de Recerca i Estudis Avançats (ICREA), Barcelona 08010, Spain
[13]Department of Molecular and Translational Medicine, University of Brescia, Brescia 25121, Italy
[14]Co-first author
[15]Co-senior author
*Correspondence: araya@cmrb.eu (A.R.), consiglio@ub.edu (A.C.)
https://doi.org/10.1016/j.stemcr.2018.12.011

SUMMARY

Parkinson's disease (PD) is associated with the degeneration of ventral midbrain dopaminergic neurons (vmDAns) and the accumulation of toxic α-synuclein. A non-cell-autonomous contribution, in particular of astrocytes, during PD pathogenesis has been suggested by observational studies, but remains to be experimentally tested. Here, we generated induced pluripotent stem cell-derived astrocytes and neurons from familial mutant *LRRK2* G2019S PD patients and healthy individuals. Upon co-culture on top of PD astrocytes, control vmDAns displayed morphological signs of neurodegeneration and abnormal, astrocyte-derived α-synuclein accumulation. Conversely, control astrocytes partially prevented the appearance of disease-related phenotypes in PD vmDAns. We additionally identified dysfunctional chaperone-mediated autophagy (CMA), impaired macroautophagy, and progressive α-synuclein accumulation in PD astrocytes. Finally, chemical enhancement of CMA protected PD astrocytes and vmDAns via the clearance of α-synuclein accumulation. Our findings unveil a crucial non-cell-autonomous contribution of astrocytes during PD pathogenesis, and open the path to exploring novel therapeutic strategies aimed at blocking the pathogenic cross talk between neurons and glial cells.

INTRODUCTION

Parkinson's disease (PD) is the second most prevalent neurodegenerative disease after Alzheimer's disease, affecting 7 to 10 million people worldwide (Global Burden of Disease Study Collaborators, 2015). PD is characterized by a significant loss of ventral midbrain dopaminergic neurons (vmDAns) in the substantia nigra pars compacta. The presence of intracellular protein aggregates of α-synuclein (α-syn) in the surviving vmDAns has been reported in postmortem PD tissue (Greenamyre and Hastings, 2004). Most PD cases are sporadic (85%), but familial mutations are accountable for 15% of patients (Lill, 2016). Mutations in the gene encoding leucine-rich repeat kinase 2 (*LRRK2*), causing an autosomal dominant form of PD, account for 5% of familial cases and 2% of sporadic cases (Gilks et al., 2005; Nichols et al., 2005). LRRK2 is a highly complex protein with both GTPase and protein kinase domains involved in several cellular functions, including autophagy (Cookson, 2016; Orenstein et al., 2013; Su et al., 2015).

Correlations between mutant LRRK2 and several pathogenic mechanisms linked to PD progression have been previously reported, including alterations in autophagy and consequent accumulation of α-syn (Cookson, 2017). Neuronal mutant LRRK2 toxicity was found to depend on LRRK2 levels and α-syn accumulation as opposed to kinase activity or inclusion bodies in induced pluripotent stem cell (iPSC)-derived neurons (Skibinski et al., 2014). During PD pathogenesis, mutant LRRK2 was found to directly bind LAMP2A, the receptor responsible for chaperone-mediated autophagy (CMA) normally used by both LRRK2 and α-syn for degradation (Orenstein et al., 2013). This binding blocks the proper functioning of the CMA translocation complex, resulting in defective CMA, leading to the accumulation of α-syn and cell death.

This is an open access article under the CC BY license (http://creativecommons.org/licenses/by/4.0/).

iPSCs derived from healthy individuals and patients have accelerated advances in developing genuinely human experimental models of diseases (Zeltner and Studer, 2015). In the case of PD, previous studies by our groups and others have generated iPSCs from patients with PD associated with *LRRK2* mutations, and described the appearance of disease-specific phenotypes in iPSC-derived neurons, including impaired axonal outgrowth and deficient autophagic vacuole clearance (Heman-Ackah et al., 2017; Nguyen et al., 2011; Sanchez-Danes et al., 2012; Skibinski et al., 2014). Moreover, dopaminergic (DA) neurons from *LRRK2*-mutant iPSCs displayed alterations in CMA that were, at least in part, responsible for the abnormal accumulation of α-syn observed in these cells, which predated any morphological signs of neurodegeneration (Orenstein et al., 2013).

Studies investigating PD pathogenesis have been mostly focused on the mechanisms underlying vmDAn degeneration and death. However, there is evidence of astrocytes accumulating α-syn during PD through postmortem analysis (Braak et al., 2007; Wakabayashi et al., 2000). Altered α-syn released by axon terminals in the surrounding synapses is taken up by astrocytes, supporting the hypothesis of the spread of α-syn through neuron-astrocyte interactions (Braak et al., 2007; Lee et al., 2010). Overexpression of mutant *SNCA* in primary astrocytes altered their normal functioning and impaired proper blood-brain barrier control and glutamate homeostasis, and eventually resulted in a significant loss of vmDAns (Gu et al., 2010). In a study using human brain homogenates from PD patients with Lewy bodies, α-syn was found to be taken up and spread from astrocytes to neurons, leading to neuronal death (Cavaliere et al., 2017). As a result, a role of astrocyte dysfunction in PD pathogenesis is emerging (Booth et al., 2017).

In the present study, we generated patient-specific iPSC-derived astrocytes and vmDAns from PD patients with the *LRRK2* G2019S mutation and healthy individuals. We consistently generated a population of human vmDAns *in vitro* that expressed postmitotic dopaminergic markers and fired action potentials. Subsequently, we co-cultured healthy iPSC-derived vmDAns with iPSC-derived astrocytes expressing the mutated form of *LRRK2* associated with PD. In co-culture experiments, we detected a significant decrease in the number of vmDAns in the presence of *LRRK2*-PD astrocytes, which correlated with the abnormal accumulation of astrocyte-derived α-syn. Conversely, control astrocytes were able to partially rescue disease-related phenotypes in *LRRK2*-PD vmDAns during co-culture. A more in-depth investigation revealed impaired autophagic machinery, as well as progressive accumulation of endogenous α-syn in PD astrocytes, compared with control astrocytes. By treating the cells with an activator of CMA, we were able to prevent the appearance of PD-related phenotypes in patients' astrocytes. Overall, our findings represent a direct indication that dysfunctional astrocytes play a crucial role during PD pathogenesis and may have broad implications for future intervention in early stages of PD.

Table 1. Summary of the Healthy Controls and Patients Used in This Study

Code	Status	Sex	Age at Biopsy	Mutation	Isogenic Control
SP09	control	M	66	no	
SP11	control	M	52	no	
SP17	control	F	48	no	
SP06	Parkinson's disease	M	44	*LRRK2* G2019S	
SP12	Parkinson's disease	F	63	*LRRK2* G2019S	
SP13	Parkinson's disease	F	68	*LRRK2* G2019S	*LRRK2* G2019S corrected

RESULTS

Generation and Characterization of iPSC-Derived Patient-Specific Astrocytes

To establish an *in vitro* human cellular model for dissecting the interplay between neurons and astrocytes in PD, we first derived astrocyte-like cells from iPSCs, using a previously published protocol (Serio et al., 2013). Specifically, astrocyte cultures were successfully established from iPSC lines from three PD patients carrying the G2019S mutation in the *LRRK2* gene (PD SP06, PD SP12, and PD SP13) and two healthy age-matched controls (Ctrl SP09 and Ctrl SP17) (see Table 1 and Tables S1 and S2 for a summary of the iPSC lines used, and Experimental Procedures for details on their origin). Immunocytochemistry (ICC) detection of key astrocyte markers showed robust expression of CD44, glial fibrillary acidic protein (GFAP), and S100 calcium-binding protein β (S100β), as well as of the excitatory amino acid transporter 2 (EAAT2, also known as GLT1), in all human iPSC-derived astrocytes (Figure 1A). No evident contamination by other cell types, such as neurons or oligodendroglial progenitors, was found as assessed by immunostaining with anti-MAP2 or NG2 antibody, respectively (Figures 1A and 1B). The astrocytic identity was further confirmed by quantitative RT-PCR of *GFAP* and additional astrocyte-specific genes, including *MLC1*, *SOX9*, *ALDH1L1*, *AQP4*, *DIO2*, and *SLC4A4*, which were expressed in Ctrl and PD astrocytes, and in human primary astrocytes, but not in iPSCs (Figures S1A–S1C).

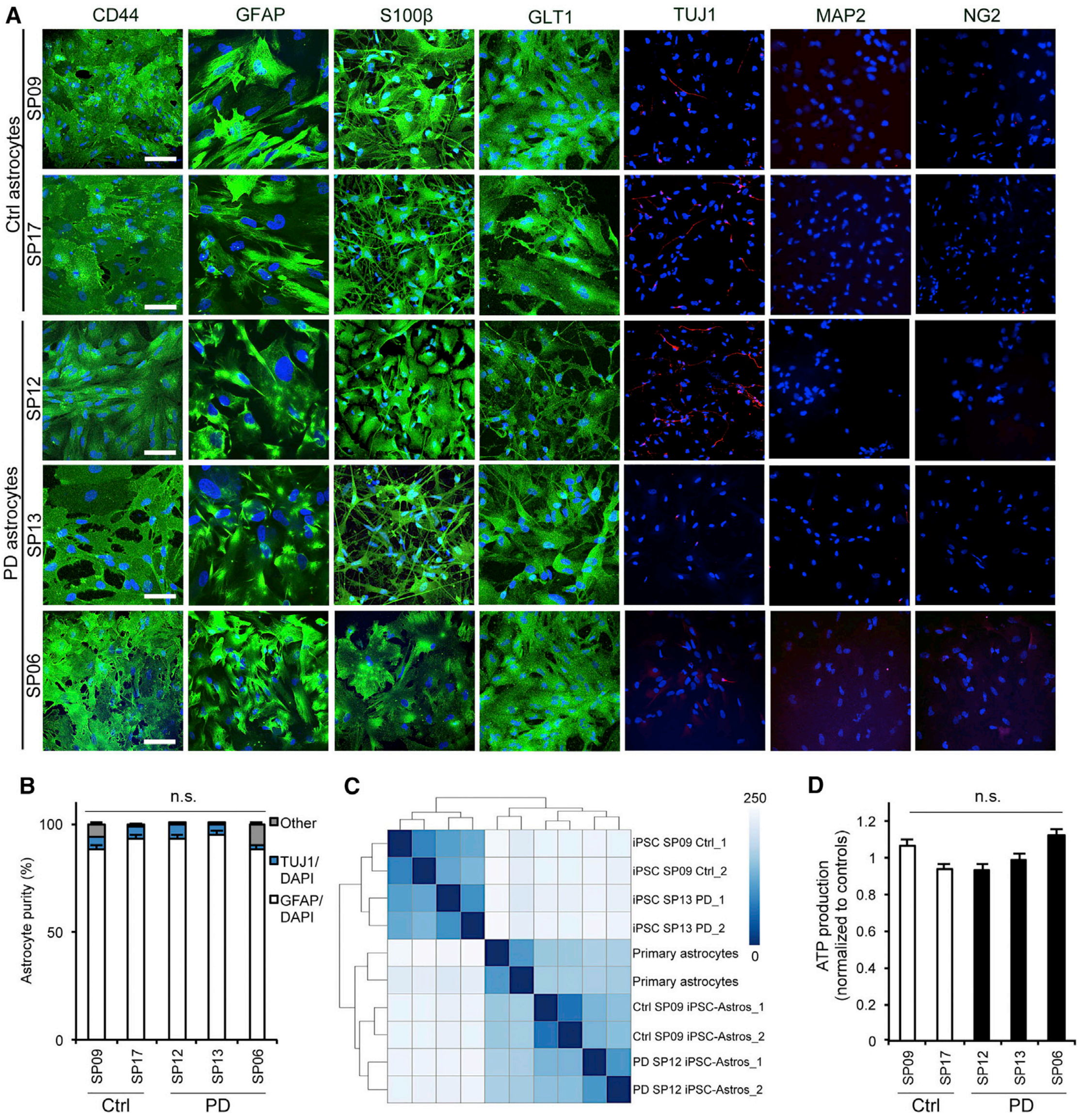

Figure 1. iPSC-Derived Patient-Specific Astrocyte Generation and Characterization

(A) Representative ICC images of astrocytes from two Ctrl iPSC lines (Ctrl SP09 and Ctrl SP17) and three PD iPSC lines (PD SP12, PD SP13, and PD SP06) staining positive for CD44 (astrocytic precursor marker), GFAP (general astrocytes), S100β (mature astrocytes), and GLT1 (excitatory amino acid transporter 2), and negative for TUJ1 (immature neurons), MAP2 (mature neurons), and NG2 (oligodendrocytes) expression. Number of independent astrocyte lines generated from iPSC per patient = 3. Number of independent experiments per astrocyte line generated = 3. Scale bar, 100 μm.

(B) Astrocyte cultures are composed of approximately 95% astrocytes, 4% neurons, and 1% other (n = 3).

(C) Heatmap showing sample similarities taking the rlog transformed data and Euclidean distances between samples. iPSC-derived astrocyte (Ctrl SP09 and PD SP12) samples cluster closer to the human primary astrocytes than the corresponding iPSC group (n = 2).

(D) Functional ATP production luminescence (counts normalized to controls) in both Ctrl (SP09 and SP17) and PD (SP13, SP12, and SP06) astrocytes (n = 3). Data are expressed as mean ± SEM, unpaired two-tailed Student's t test.

To validate astrocyte cell type identity, we also tested the expression of astrocyte-specific genes revealed by the Human Astrocyte RNA-Seq database (www.brainrnaseq.org/) in our iPSC-derived astrocytes, through RNA sequencing. We found that the transcriptomic profile of both Ctrl and PD iPSC-derived astrocytes was closer to that of human primary astrocytes than to that of their corresponding iPSC line, thus confirming their identity (Figure 1C). We next determined the functional maturation of iPSC-derived astrocytes by confirming their capacity

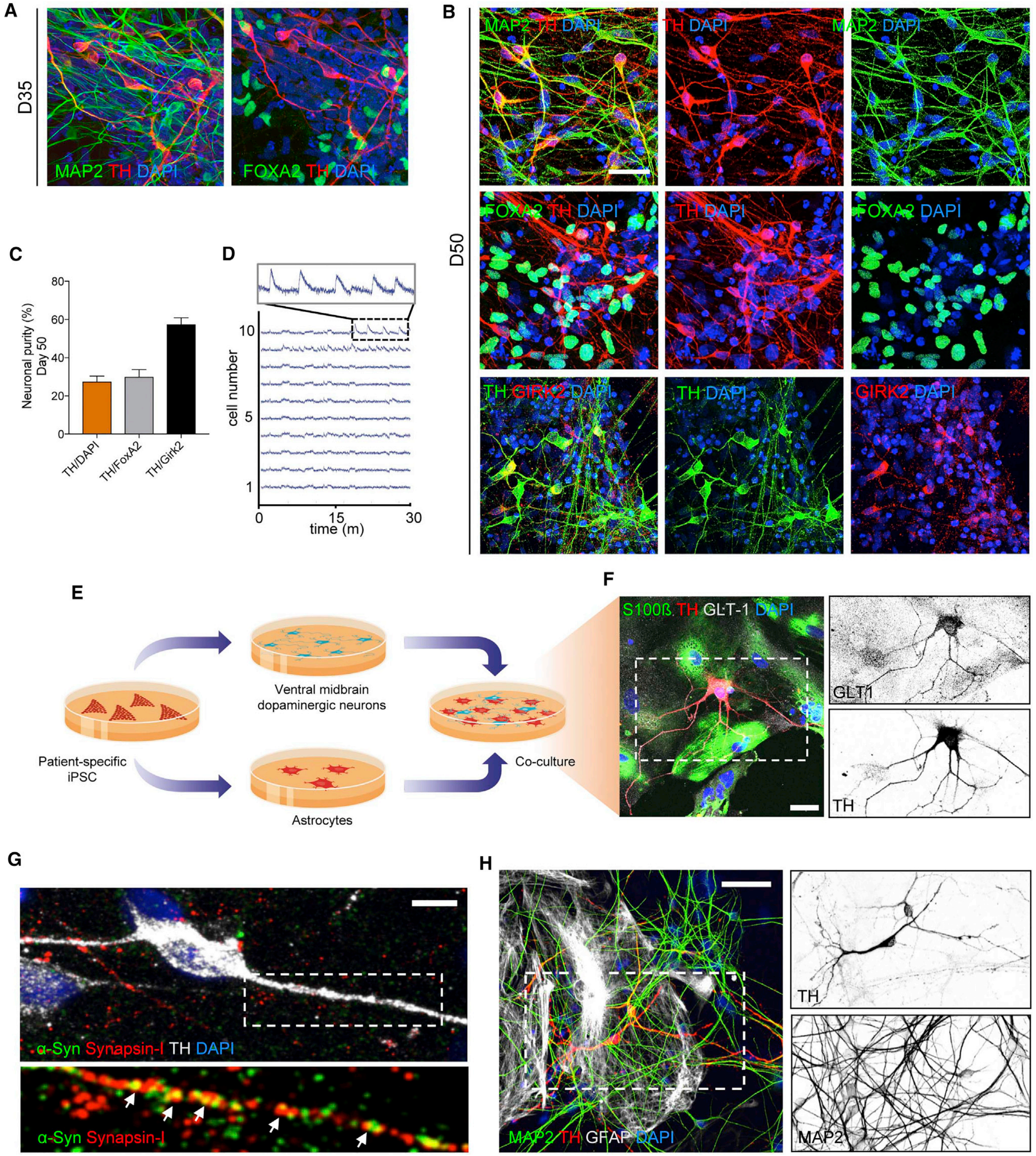

Figure 2. vmDAn Generation, Characterization, and Co-culture Setup

(A and B) Representative immunofluorescence images of Ctrl SP11 vmDAn after (A) 35 or (B) 50 days of neuronal differentiation. iPSC-derived neural cultures express markers specific for neurons (MAP2), DAns (TH), and midbrain-type DAns (FOXA2 and GIRK2). Scale bar, 20 μm.

(C) Percentage of differentiated cells that stained positive for TH and double positive for TH and FOXA2 and TH and GIRK2 after 50 days of differentiation (n = 4).

(legend continued on next page)

to produce ATP (Figure 1D) and propagate intercellular Ca^{2+} waves (Figures S1D–S1H). Indeed, by using the Fluo-4 AM Ca^{2+} indicator, recordings from Ctrl and PD astrocytes showed a heterogeneous pattern of Ca^{2+} fluctuations under basal conditions, revealing their functionality. All together, these data support the successful generation of highly pure populations of functionally equivalent astrocyte-like cells, which represent a continuous source of human Ctrl and PD astrocytes for subsequent analyses.

Generation of vmDAns and Setup of Neuron-Astrocyte Co-culture System

To investigate whether astrocytes contribute to PD pathogenesis, we established a co-culture system of iPSC-derived astrocytes and iPSC-derived vmDAns. PD vmDAns were generated from iPSC lines from two PD patients carrying the G2019S mutation in the *LRRK2* gene (iPSC lines PD SP12 and PD SP13), whereas Ctrl vmDAns were obtained from two independent iPSC clones (Ctrl SP11 and Ctrl SP11#4) from a healthy age-matched control (see Tables 1, S1, and S2 for a summary of the iPSC lines used). To differentiate iPSCs toward vmDAns, we used a combination of two previously published (Chambers et al., 2009; Kriks et al., 2011) midbrain floor-plate differentiation protocols that was comparably effective in all iPSC lines analyzed. Under these conditions, ~20% of cells in the cultures were committed to DA neuron fate by day 35 of differentiation, as judged by the expression of tyrosine hydroxylase (TH) and forkhead box protein A2 (FOXA2) (Figure 2A). By day 50 of differentiation, the percentage of TH^{+} neurons reached ~30%, most of which also expressed the A9-domain-specific marker G-protein-activated inward rectifier potassium channel 2 (GIRK2), and displayed spontaneous action potential firing (Figures S2B–S2D). For co-culture experiments with control astrocytes, we dissociated vmDAn cultures after 35 days of differentiation and plated them onto a confluent layer of Ctrl iPSC-derived astrocytes (Figure 2E). After 4 weeks of co-culture, we found that astrocyte-neuron glutamate exchange was present through glutamate transporter 1 (GLT1) expression (Figure 2F) and neuronal synapse formation (Figure 2G). Accordingly, an overall healthy neuronal network comprising MAP2-positive cells was formed upon co-culture (Figure 2H).

Control vmDAns Show Morphological Signs of Neurodegeneration when Co-cultured with PD Astrocytes

We then examined the effects of astrocytes expressing mutated *LRRK2* on the survival of Ctrl iPSC-derived vmDAns upon co-culture (Figure 3A). After 2 weeks of co-culture with PD astrocytes, Ctrl vmDAns displayed morphological alterations, including shortened neurites, and significantly decreased cell survival compared with co-cultures with Ctrl astrocytes (Figures S2A–S2C). These alterations were much more evident after 4 weeks of co-culture, when Ctrl vmDAns cultured on top of PD astrocytes showed extensive signs of neurodegenerative phenotypes (fewer and shorter neurites, and abundance of beaded-necklace neurites) and severely compromised cell survival (less than ~25% of control), compared with co-cultures with control astrocytes (Figures 3B–3F). The fact that the numbers of vmDAns did not change significantly during the co-culture with control astrocytes, but progressively declined when co-cultured with PD astrocytes, strongly suggests that vmDAns were lost under the latter conditions as a result of neurodegeneration, rather than a blockade in vmDAn differentiation or maturation.

Viability tests of both Ctrl and PD astrocytes at 2 and 4 weeks of co-culture revealed highly similar values, indicating that neurodegenerative signs displayed by Ctrl vmDAns were not caused by a dying PD astrocyte (Figure S2D). Interestingly, vmDAn neurodegeneration upon co-culture with PD astrocytes was specific to this type of neuron, because non-dopaminergic neurons ($TH^{-}/MAP2^{+}$) did not significantly change in numbers or morphology after co-culture with Ctrl or PD astrocytes (Figures S2E–S1J). All together, these results indicate a neurotoxic capacity of PD astrocytes toward vmDAns, with no effects on other neuronal types concomitantly present in cultures.

Control vmDAns Accumulate α-syn when Co-cultured with PD Astrocytes

Given the relevance of α-syn in the context of PD pathogenesis (Braak et al., 2007), we sought to examine whether

(D) Calcium wave flux recording over 30 min with calcium tracer Fluo-8 AM of vmDAns at day 50 (n = 3).
(E) Diagram of co-culture system.
(F) Representative ICC images of 4-week co-culture staining positive for Ctrl SP11 vmDAns (TH), Ctrl SP17 astrocytes (S100β), excitatory amino acid transporter 2 (GLT1), and nuclear DAPI. Scale bar, 20 μm.
(G) Representative ICC images of presynaptic markers α-syn and synapsin-1 of a Ctrl SP11 vmDAn (TH) on the top of Ctrl SP11 astrocytes after 4 weeks in co-culture. Scale bar, 10 μm.
(H) Representative ICC images of Ctrl SP11 vmDAns (TH) and mature neurons (MAP2) on the top of Ctrl SP09 astrocytes (GFAP) during a 4-week co-culture period. Scale bar, 20 μm.
Boxed area on the left in (F), (G), and (H) is shown on the right.

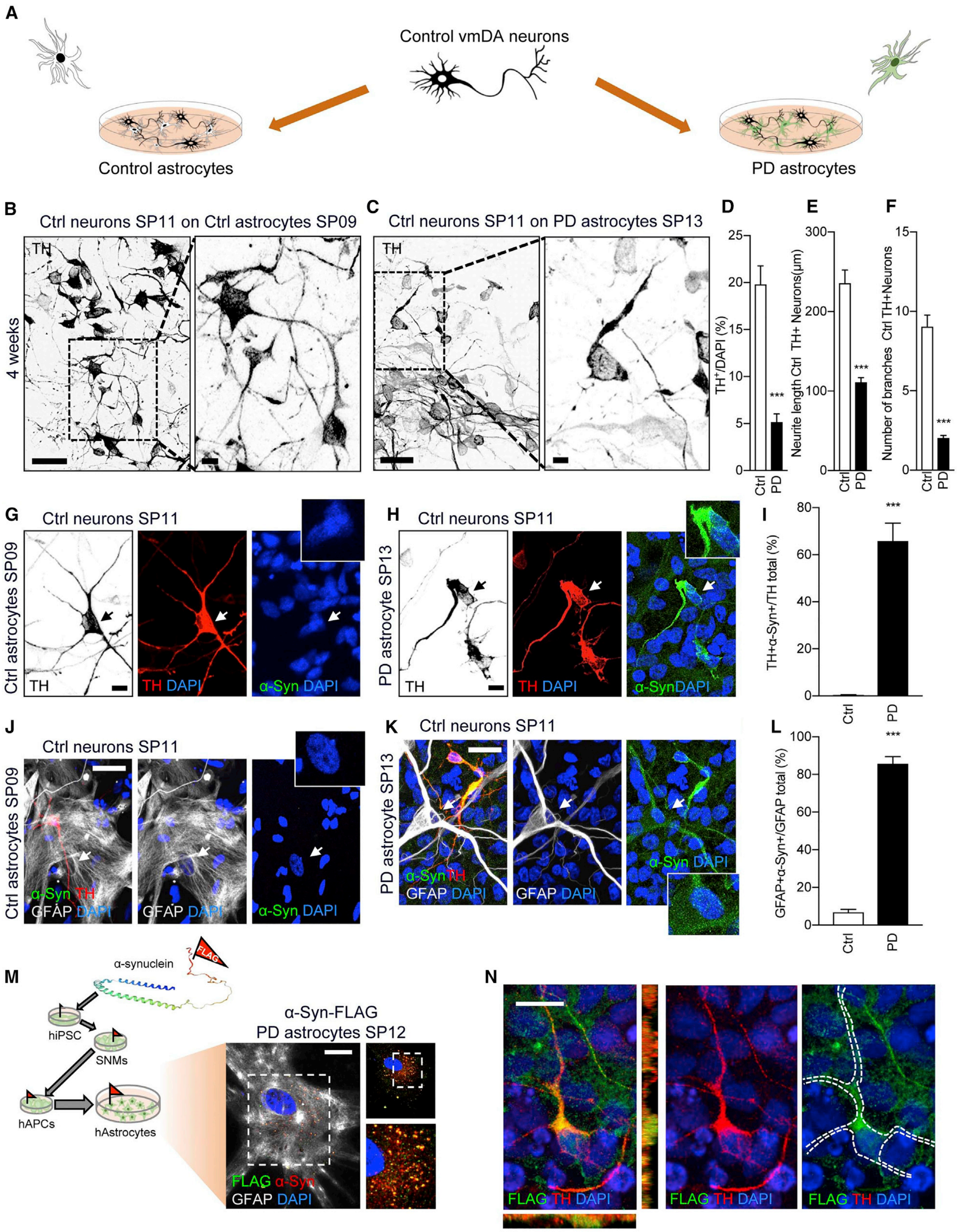

(legend on next page)

vmDAns co-cultured with PD astrocytes abnormally accumulated α-syn. α-syn was barely detectable in the cytoplasm of Ctrl vmDAns cultured alone (data not shown) or when co-cultured with Ctrl astrocytes (Figure 3G). In contrast, Ctrl vmDAns accumulated high levels of α-syn throughout the neurites and cell body after 4 weeks of co-culture with PD astrocytes (Figures 3H and 3I). Notably, while Ctrl astrocytes had undetectable levels of α-syn (Figure 3J), PD astrocytes displayed high levels of α-syn (Figures 3K and 3L), raising the intriguing possibility that α-syn from PD astrocytes might be transferred to Ctrl vmDAns. To directly address if this was the case, we genetically engineered two iPSC lines (representing one PD patient and one healthy control) using CRISPR/Cas9 technology so that the endogenous α-syn would be tagged with a FLAG peptide (α-syn-FLAG iPSC lines; Figures S2K and S2L and Tables S2 and S3). α-syn-FLAG-tagged astrocytes were generated and fully characterized (Figure S2M). As expected, PD α-syn-FLAG tagged astrocytes accumulated abnormally high levels of α-syn, which co-localized with anti-FLAG staining (Figure 3M). More importantly, the co-culture of Ctrl vmDAns on top of α-syn-FLAG-tagged PD astrocytes for 4 weeks resulted in FLAG-tagged α-syn accumulation in neurons, demonstrating the direct transfer of astrocytic α-syn to neurons (Figure 3N).

In addition to co-culturing cells with direct glia-neuron contact, we tested the effect of culturing Ctrl vmDAns with medium conditioned by Ctrl or PD astrocytes at different concentrations (Figure S3A). Exposure of Ctrl vmDAns to PD astrocyte-conditioned medium for 1 week, even at low concentrations, resulted in α-syn accumulation, morphological alterations suggestive of neurodegeneration, and decreased cell survival (Figures S3B–S3H), indicating that PD astrocytes secrete a molecule(s) that is toxic to vmDAns. Direct uptake by vmDAns of α-syn from conditioned medium was tested by exposing Ctrl vmDAns to medium collected from PD α-syn-FLAG-tagged astrocytes (Figures S3I–S3J), suggesting that the neurotoxic effect of PD astrocytes on vmDAns is, at least in part, mediated by secretion of α-syn.

Control Astrocytes Partially Rescue Neurodegeneration of PD vmDAns

We have previously shown that vmDAns derived from PD-iPSCs show signs of neurodegeneration (including reduced numbers of neurites and neurite arborization, as well as accumulation of abnormal α-syn in the soma) after 50 days of culture, which are not evident in Ctrl vmDAns (Sanchez-Danes et al., 2012). To test whether the neurodegeneration could be rescued or prevented by healthy astrocytes, we co-cultured PD vmDAns with Ctrl or PD astrocytes. After a 4-week co-culture, PD vmDAns showed a partially recovered neurite number and complex neurite arborization when co-cultured on control astrocytes, compared with co-cultures with PD astrocytes (Figures 4A–4E). This rescue was partial, since PD vmDAns

Figure 3. Ctrl Neurons Show Signs of Neurodegeneration and Accumulate α-syn when Co-cultured with PD Astrocytes

(A) Scheme representing co-culture system of Ctrl neurons on the top of Ctrl or PD astrocytes for 4 weeks.

(B and C) Representative ICC images of tyrosine hydroxylase (TH, black) from co-cultures of Ctrl SP11 neurons with (B) Ctrl SP09 astrocytes and (C) PD SP13 astrocytes for 4 weeks. Images on the right show a magnification of the area boxed in the left images; scale bars, (left) 20 μm and (right) 0.2 μm.

(D) Percentage of TH^+/DAPI of Ctrl SP11 and Ctrl SP11#4 neurons when co-cultured with Ctrl SP09, Ctrl SP17, or PD SP12, PD SP13, and PD SP06 astrocytes for 4 weeks (n = 3 per combination).

(E and F) (E) Neurite length quantification and (F) number of branches of Ctrl SP11 TH-positive neurons and Ctrl SP11#4 TH-positive neurons when co-cultured on Ctrl SP09, Ctrl SP17, and Ctrl SP11 astrocytes or PD SP12, PD SP13, and PD SP06 astrocytes for 4 weeks (n = 3); 30 neurons counted per experiment.

(G and H) Representative ICC images of Ctrl SP11 vmDAns co-cultured with (G) Ctrl SP09 and (H) PD SP13 astrocytes after 4 weeks and stained for TH (vmDAn), α-syn, and DAPI. Arrows indicate the selected cell for which an insert is shown at higher magnification. Scale bar, 0.2 μm.

(I) Quantitative analysis of the percentage of vmDAns stained positive for α-syn when Ctrl SP11 and Ctrl SP11#4 neurons were co-cultured with Ctrl SP09, Ctrl SP17, or PD SP12, PD SP13, and PD SP06 astrocytes for 4 weeks (n = 3).

(J and K) Representative ICC images of (J) Ctrl SP09 or (K) PD SP13 astrocytes co-cultured with Ctrl SP11 vmDAns for 4 weeks, stained for TH (vmDAn), GFAP (astrocytes), α-syn, and DAPI. Arrows indicate the selected cell for which an insert is shown at higher magnification. Scale bar, 20 μm.

(L) Quantitative analysis of the percentage of astrocytes stained positive for α-syn when Ctrl SP11 and Ctrl SP11#4 neurons were co-cultured with Ctrl SP09, Ctrl SP17, or PD SP12, PD SP13, and PD SP06 astrocytes for 4 weeks (n = 3).

(M) Scheme representing the generation of CRISPR/Cas9 edited α-syn-FLAG astrocyte line. Representative image of α-syn-FLAG PD SP12 astrocyte (GFAP) showing perfect α-syn (red) and FLAG (green) co-localization. Scale bar, 20 μm.

(N) Representative ICC image depicting astrocyte-derived FLAG (green) inside of a TH-positive Ctrl SP11 neuron (red) during a 4-week co-culture period with PD SP12 α-syn-FLAG astrocytes (n = 3). Dashed line shows the outline of the cell. Scale bar, 10 μm.

Data are expressed as mean ± SEM, unpaired two-tailed Student's t test, ***$p < 0.001$.

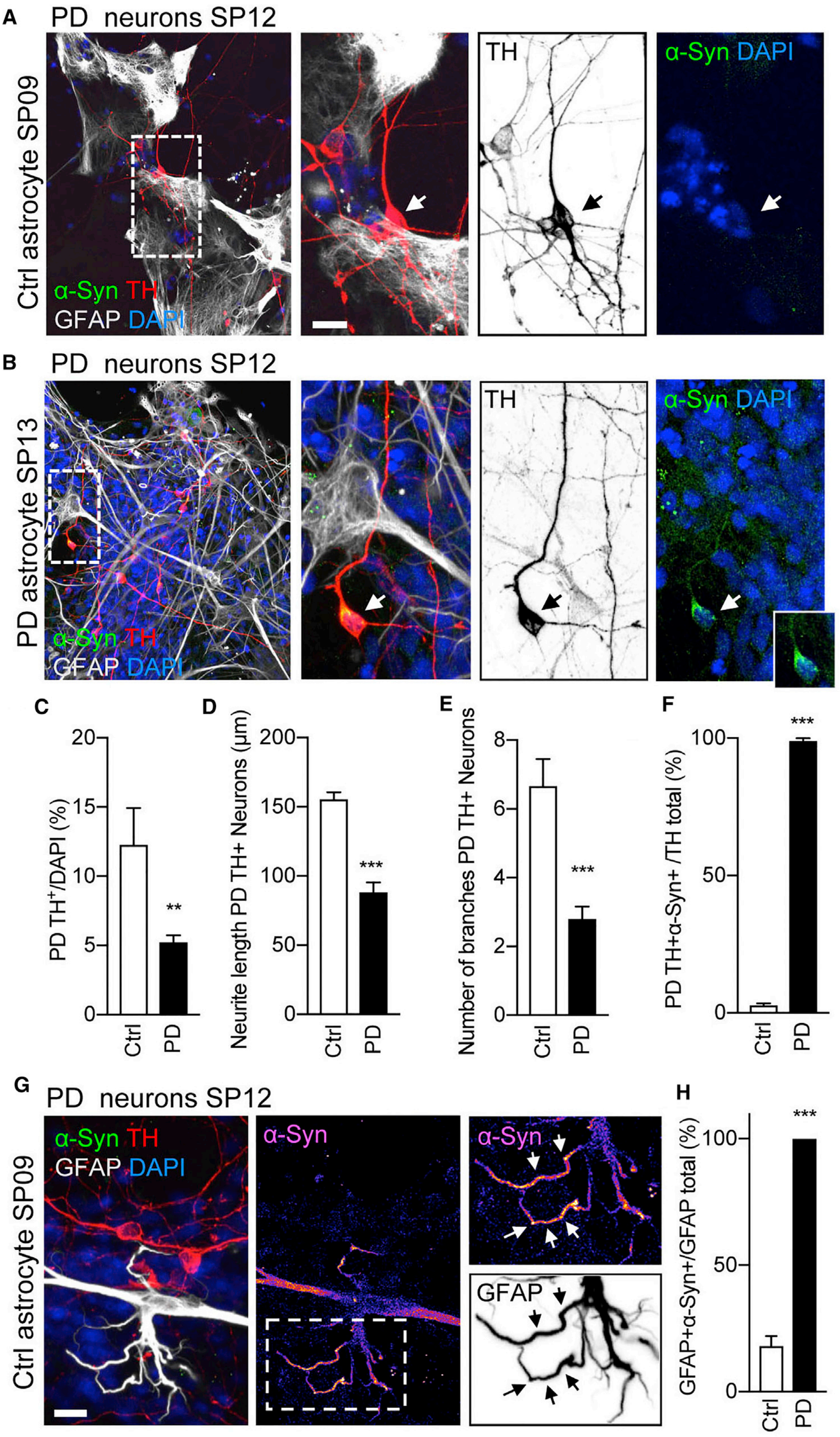

(legend on next page)

co-cultured with control astrocytes did not reach the levels of cell survival and complex neurite arborization seen in co-cultures of Ctrl vmDAns and Ctrl astrocytes (compare Figures 4A and 4C–4E with 3B and 4D–4F). Moreover, co-culture with Ctrl astrocytes also prevented the accumulation of α-syn in PD vmDAns that was evident in co-cultures with PD astrocytes (Figures 4A, 4B, and 4F). Notably, most Ctrl astrocytes when co-cultured with PD vmDAns adopted a flat morphology with moderate levels of α-syn; however, some harbored a hypertrophic morphology with retracted processes that accumulated very high levels of α-syn (Figures 4G and 4H), suggesting that reactive astrocytes may contribute to the clearance of vmDAn α-syn accumulation. Culture of PD vmDAns with medium conditioned by Ctrl astrocytes also rescued cell survival, morphological alterations, and α-syn accumulation (Figures S3K–S3S), indicating that direct neuronal-glial contact was not necessary for the neuroprotective effect.

We next investigated the causative role of the genetic background of patient-specific astrocyte cells by ectopically expressing mutated *LRRK2* G2019S in Ctrl astrocytes. In these experiments, Ctrl astrocytes were transfected with a plasmid expressing V5-tagged *LRRK2* G2019S, or with a Ctrl plasmid expressing GFP, and analyzed 7 days after transfection for the presence of α-syn. Astrocytes transfected with *LRRK2* G2019S exhibited diffuse cytoplasmic accumulations of α-syn (Figure S4A), which were not present in GFP-transfected cells (Figure S4B). The transfection efficiencies (30%–40%, as evaluated by co-staining for V5/GFAP or GFP/GFAP) were comparable under both conditions (Figure S4C). Next, we co-cultured Ctrl vmDAns for 4 weeks with *LRRK2* G2019S-transfected Ctrl astrocytes, or with GFP-transfected astrocytes as a control, and we found α-syn accumulation in 50% of the TH^+ neurons only in co-cultures with *LRRK2* G2019S-transfected astrocytes (Figures S4D–S4G). Overall in these cultures we found decreased survival of vmDAns and evident morphological alterations (Figures S4H–S4I), including fewer and shorter neurites compared with vmDAns cultured on top of GFP-transfected astrocytes, indicating that the expression of pathogenic LRRK2 in Ctrl astrocytes is deleterious for the survival of dopaminergic neurons.

For the converse experiment, we generated isogenic PD astrocytes lacking the *LRRK2* G2019S mutation by CRISPR/Cas9-mediated gene editing of PD iPSCs (iPSC line PD SP13, from here on referred to as PD iso), and fully characterized these cells (Figures S5A–S5E). Abnormal α-syn accumulation did not occur in gene-corrected astrocytes, in contrast with their isogenic mutant counterparts (Figures S5F and S5G). Moreover, co-culturing gene-corrected astrocytes with Ctrl vmDAns for 4 weeks prevented the accumulation of α-syn and decrease in neuron survival observed when Ctrl vmDAns were co-cultured with PD astrocytes (Figures S5H–S5J), further supporting that the expression of mutant *LRRK2* in astrocytes is pathogenic to Ctrl vmDAns.

Dysfunctional Chaperone-Mediated Autophagy and Progressive α-syn Accumulation in PD Astrocytes

Since PD astrocytes displayed higher levels of α-syn compared with controls, we next investigated possible differences in α-syn turnover in these cells. Degradation of α-syn in lysosomes occurs in large extent through CMA (Cuervo et al., 2004; Martinez-Vicente et al., 2008). To investigate possible changes in CMA in PD astrocytes, we first stained at 6 and 14 days for both α-syn and LAMP2A, the receptor for CMA (Figures 5A and S6A). Ctrl astrocytes showed LAMP2A in the perinuclear area (perinuclear lysosomal positioning occurs during CMA activation; Kiffin et al., 2004) and barely detectable levels of α-syn at both 6 and 14 days (Figures 5A, 5B, and S6A). In contrast, PD astrocytes displayed LAMP2A-positive vesicles all around

Figure 4. PD Neurons Restore Arborized Morphology and Accumulate Less α-syn when Co-cultured with Ctrl Astrocytes

(A and B) Representative ICC images of PD SP12 vmDAns during 4-week co-cultures with (A) Ctrl SP09 or (B) PD SP13 astrocytes stained for TH (vmDAns), α-syn, GFAP (astrocytes), and DAPI. Images on the right show a magnification of the area boxed in the left images. Arrows indicate the selected cell for which an insert is shown at higher magnification with α-syn accumulation. Scale bar, 20 μm.

(C) Quantitative analysis of the percentage of PD SP12 vmDAns remaining after 4-week co-culture with Ctrl SP09, PD SP13, and PD SP12 astrocytes (n = 3).

(D and E) (D) Neurite length quantification and (E) number of branches of PD SP12 TH-positive neurons when co-cultured on PD SP12 or PD SP13 astrocytes for 4 weeks compared with the wild-type condition Ctrl SP11 neurons on Ctrl SP09, Ctrl SP17, and Ctrl SP11 astrocytes for 4 weeks (n = 3); 40 neurons counted per experiment.

(F) Quantitative analysis of the percentage of PD SP12 vmDAns that stained positive for α-syn when co-cultured on the top of Ctrl SP09, Ctrl SP17, Ctrl SP11, PD SP13, and PD SP12 astrocytes for 4 weeks (n = 3).

(G) Immunofluorescence analysis of PD SP12 neurons on the top of Ctrl SP09 astrocytes stained for TH, GFAP, α-syn, and DAPI. Images on the right show a magnification of the area boxed in the left image. Arrows in the inset shows α-syn accumulation inside Ctrl SP09 astrocyte processes. Inset scale bar, 20 μm.

(H) Quantitative analysis of the percentage of astrocytes that stained positive for α-syn after being cultured with PD SP12 neurons for 4 weeks. Ctrl astrocytes were derived from SP09, SP11, and SP17 iPSCs, while PD astrocytes were derived from SP12 and SP13 iPSCs (n = 3). Data are expressed as mean ± SEM, unpaired two-tailed Student's t test, $^{**}p < 0.01$, $^{***}p < 0.001$).

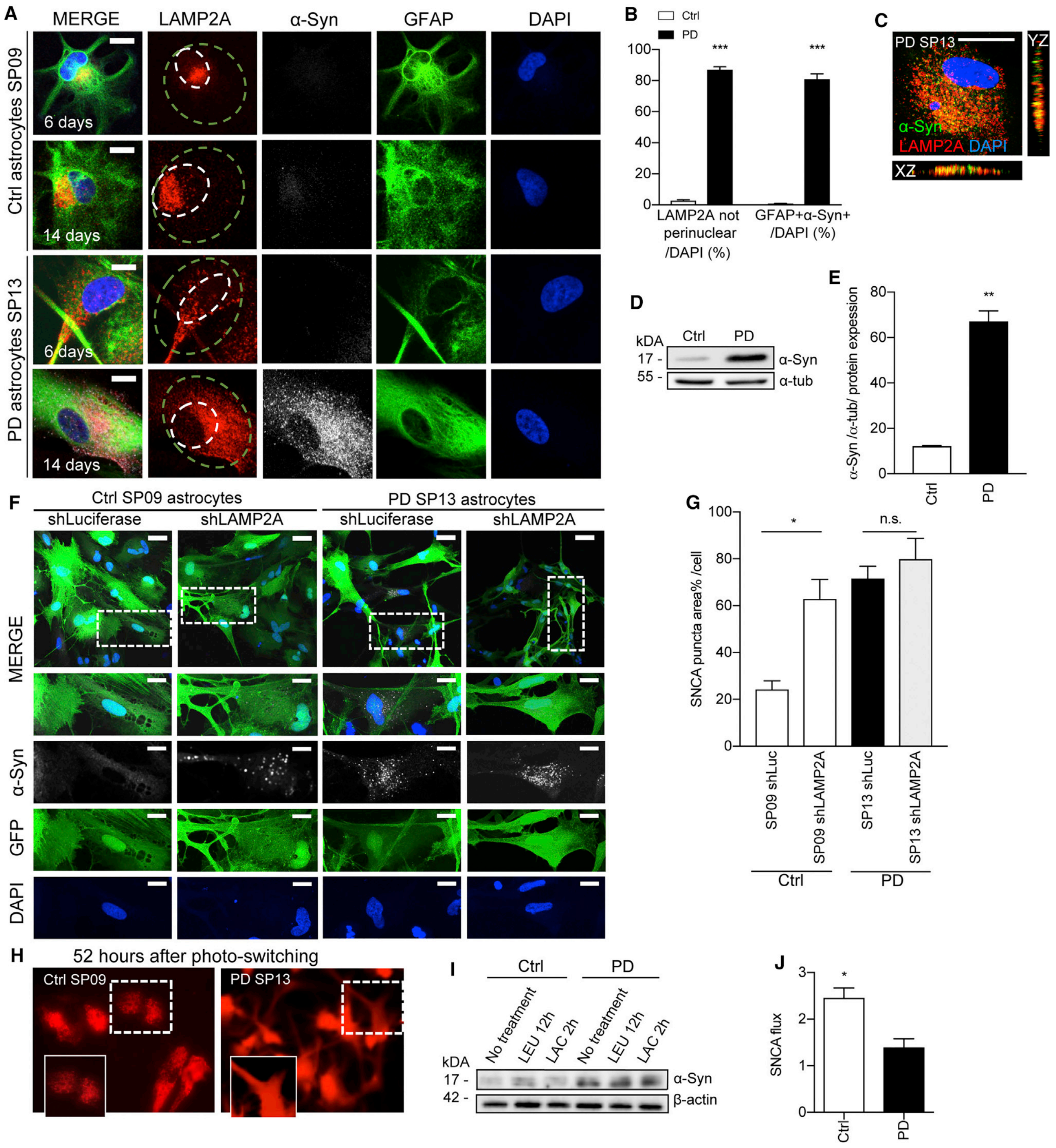

Figure 5. Altered CMA and α-syn Accumulation in LRRK2-PD Astrocytes

(A) Representative ICC images of CMA receptor (LAMP2A), astrocyte marker GFAP, α-syn, and nuclear marker DAPI in Ctrl SP09 and PD SP13 astrocytes at 6 and 14 days. Scale bar, 20 μm. Smaller white circles represent perinuclear area, whereas larger green circle represents non-perinuclear area.

(B) Percentage of astrocytes with LAMP2A-positive puncta positioning outside of perinuclear area and percentage of astrocytes that stained positive for α-syn. Astrocyte lines used in the experiment were Ctrl SP09, Ctrl SP17, PD SP12, and SP13 (n = 3).

(C) Representative ICC image of positive co-localization of LAMP2A and α-syn in PD SP13 astrocytes. Scale bar, 10 μm.

(legend continued on next page)

the cell body as early as 6 days, which continued to be present after 14 days (Figures 5A, 5B, and S6A). Moreover, abnormal accumulation of α-syn was confirmed in PD astrocytes after 14 days of culture, compared with Ctrl astrocytes (Figures 5A–5E). Interestingly, this accumulation was not present after 6 days of culture, suggesting progressive α-syn accumulation over the 14-day time period. Co-localization analyses of α-syn with the LAMP2A receptor revealed a positive co-localization that was more evident in PD astrocytes (Figures 5C and S6B). CMA substrates are usually rapidly internalized and degraded inside lysosomes, but we have previously described a similar persistent association of α-syn with LAMP2A-positive lysosomes in PD models due to blockage in α-syn translocation inside lysosomes (Orenstein et al., 2013). These findings suggest, thus, a similar CMA blockage in the PD astrocytes at the receptor level. Also supportive of reduced α-syn degradation, western blot analysis confirmed a higher monomeric protein level of α-syn in PD astrocytes compared with controls (Figures 5D, 5E, and S7A). By using an antibody that detects specifically oligomeric α-syn, we were able to detect other pathogenic forms of α-syn in PD astrocytes, which were similar to those of PD postmortem brain tissue (Figure S7B).

To investigate the contribution of the defect in CMA to the progressive accumulation of α-syn in PD astrocytes, we next performed a knockdown of LAMP2A using lentiviral-mediated short hairpin RNA (shRNA) targeting and silencing the LAMP2A spliced transcript (shLAMP2A), or an shRNA targeting the Luciferase gene (shLuc) as a control (Figure 5F). The shLuc control astrocytes displayed an expected low level of α-syn, whereas after shLAMP2A transduction, there was a statistically significant ($p < 0.001$) 2.5-fold increase in α-syn puncta, comparable to the levels observed in PD astrocytes (Figure 5G). Knockdown of LAMP2A did not change α-syn puncta levels in PD astrocytes, further suggesting defective CMA for α-syn in these cells. CMA activity was monitored using a photoactivatable CMA reporter, KFREQ-Dendra (Koga et al., 2011), in all astrocyte lines at 52 hr after photoactivation (Figures 5H and S6C). KFREQ-Dendra is present in the cytosol (diffuse fluorescent pattern) but as it is delivered to lysosomes via CMA it changes to a fluorescent punctate pattern. Ctrl astrocytes displayed these puncta, indicative of functional CMA, whereas the signal in PD astrocytes remained diffused in the cytosol, suggestive of an inactive CMA.

Since PD astrocytes displayed higher levels of α-syn compared with Ctrl astrocytes, we next investigated possible differences in α-syn turnover in these cells. α-syn has previously been shown to undergo degradation both by the ubiquitin/proteasome system and by autophagy (Cuervo et al., 2004; Webb et al., 2003); therefore α-syn flux in the presence of lysosomal and proteasome inhibitors (leupeptin and lactacystin, respectively) was evaluated in Ctrl and PD astrocytes at 14 days (Figures 5I, 5J, and S7D). An increase of ~40% in α-syn levels was found in Ctrl astrocytes after leupeptin treatment, while this increase was not found in PD astrocytes analyzed under the same conditions, indicating an impaired flux. No changes were found in either Ctrl or PD astrocytes after lactacystin treatment (Figures 5I, 5J, and S7D). These findings suggest major alterations in α-syn proteostasis due to poor degradation by lysosomal systems in PD astrocytes.

Impaired Macroautophagy in PD Astrocytes

Cells often respond to blockage in CMA by upregulating other autophagic pathways such as macroautophagy (Massey et al., 2006; Schneider et al., 2015). However, altered macroautophagy has also been reported in the context of PD (Sanchez-Danes et al., 2012; Winslow et al., 2010). To investigate the status of macroautophagy, the endo/lysosomal marker LAMP1, autophagosome marker LC3, astrocyte marker GFAP, and nuclear DAPI were used during ICC on all astrocyte lines at both 6 and 14 days. In Ctrl astrocytes, there was lysosomal LAMP1 staining in the perinuclear area and very few visible autophagic vacuoles (LC3-positive vesicles) at both 6 and 14 days (Figures 6A, 6B, and S6D). In PD astrocytes, as for LAMP2A, LAMP1-positive vesicles lost the preferable perinuclear distribution and were found throughout the entire cell (Figures 6A, 6B,

(D and E) (D) Western blot of α-syn and α-tubulin as a loading control and (E) quantification in Ctrl SP09 and PD SP13 astrocytes after 14 days in culture (n = 4).
(F) Representative ICC images of Ctrl SP09 and PD SP13 astrocytes after 14 days of transduction with either LV-shLAMP2A or LV-shLuciferase (as a control) stained for α-syn, GFP, and DAPI. Boxed areas highlight the region for which high magnification images are shown. Scale bars, 20 and 10 μm, respectively.
(G) Percentage of α-syn puncta area per cell in Ctrl SP09 and PD SP13 astrocytes transduced with LV-shLuciferase or LV-shLAMP2A (n = 3).
(H) KFERQ-DENDRA (CMA reporter) in Ctrl SP09 and PD SP13 astrocytes 52 hr after photo-switching with UV light (n = 3). Images in the insets at the bottom are a magnification of the boxed area.
(I and J) (I) Western blot of α-syn and β-actin as a loading control and (J) quantification of α-syn flux ratio normalized to β-actin in Ctrl SP09 and PD SP13 after the addition of inhibitors of lysosomal proteolysis (leupeptin [LEU], 100 μM) for 12 hr and proteasomal degradation (lactacystin [LAC], 5 μM) for 2 hr (n = 3).
Data are expressed as mean ± SEM, unpaired two-tailed Student's t test, *$p < 0.05$, **$p < 0.01$, ***$p < 0.001$.

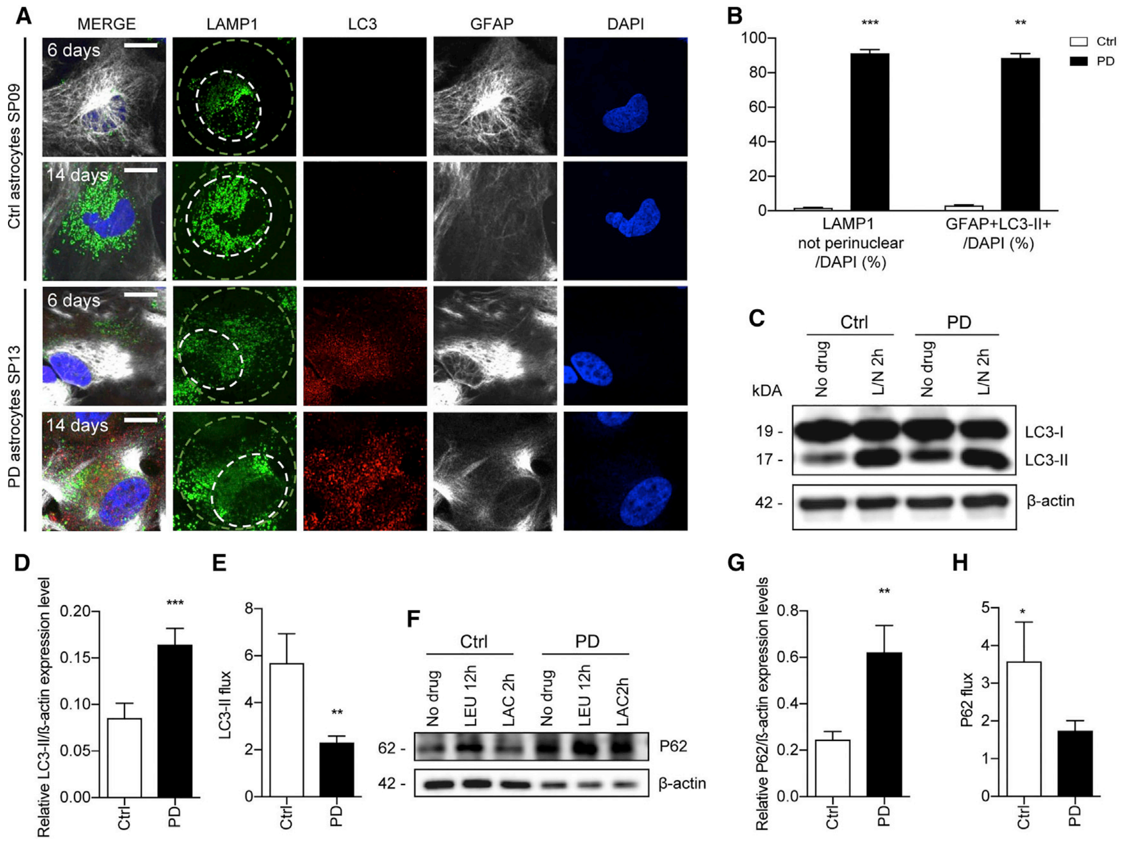

Figure 6. Dysfunctional Macroautophagy in LRRK2-PD Astrocytes

(A) Representative ICC images of lysosomal protein marker LAMP1 and autophagosome marker LC3 in Ctrl SP09 and PD SP13 astrocytes (GFAP) at 6 and 14 days. Smaller white circles represent perinuclear area, whereas larger green circle represents non-perinuclear area. Scale bar, 20 μm.

(B) Percentage of astrocytes with LAMP1-positive puncta positioning outside of perinuclear area and percentage of astrocytes that stained positive for LC3-II. Astrocyte lines used in the experiment were Ctrl SP09 and SP13 (n = 3).

(C–E) (C) Western blot of LC3-II protein levels and β-actin as loading control with corresponding quantification of (D) the LC3-II basal expression and (E) LC3-II flux with or without lysosomal inhibitors NH_4Cl and leupeptin (L/N) for 2 hr in Ctrl SP09 and PD SP13 astrocytes (n = 3).

(F–H) (F) Western blot of p62 protein levels and β-actin as loading control with corresponding quantification of (G) the P62 basal expression and (H) P62 flux without inhibitors or with inhibitors leupeptin for 12 hr and lactacystin for 2 hr in Ctrl SP09 and PD SP13 astrocytes (n = 3).

Data are expressed as mean ± SEM, unpaired two-tailed Student's t test, $*p < 0.05$, $**p < 0.01$, $***p < 0.001$.

and S6D). In addition, there was a marked increase in autophagic vacuoles starting as early as 6 days that continued increasing through the 14-day time point (Figures 6A, 6B, and S6D). Most of the accumulated LC3-positive vesicles in PD astrocytes did not co-localize with LAMP1 lysosomes (Figures S6E and S6F), suggesting that they were autophagosomes that persisted in these cells due to their poor clearance by lysosomes.

In agreement with the immunofluorescence studies, western blot analyses detected higher basal levels of LC3-II in PD astrocytes compared with Ctrls (Figures 6C, 6D, and S7C). To monitor the autophagy flux and to gain insights into the mechanism behind the accumulated LC3-II levels in PD astrocytes, both Ctrl and PD astrocytes were treated with leupeptin and NH_4Cl, inhibitors of lysosomal proteolysis, to inhibit LC3-II degradation. Under these conditions, PD astrocytes exhibited lower increase in LC3-II levels compared with controls, suggesting an impairment of the autophagy flux in PD astrocytes (Figures 6E and S7C). Moreover, we found higher p62 levels in PD astrocytes at baseline compared with controls, and lower flux ratio in the presence of inhibitor (Figures 6F–6H and S7E). Overall these findings suggest that reduced function in both autophagic pathways, CMA and macroautophagy,

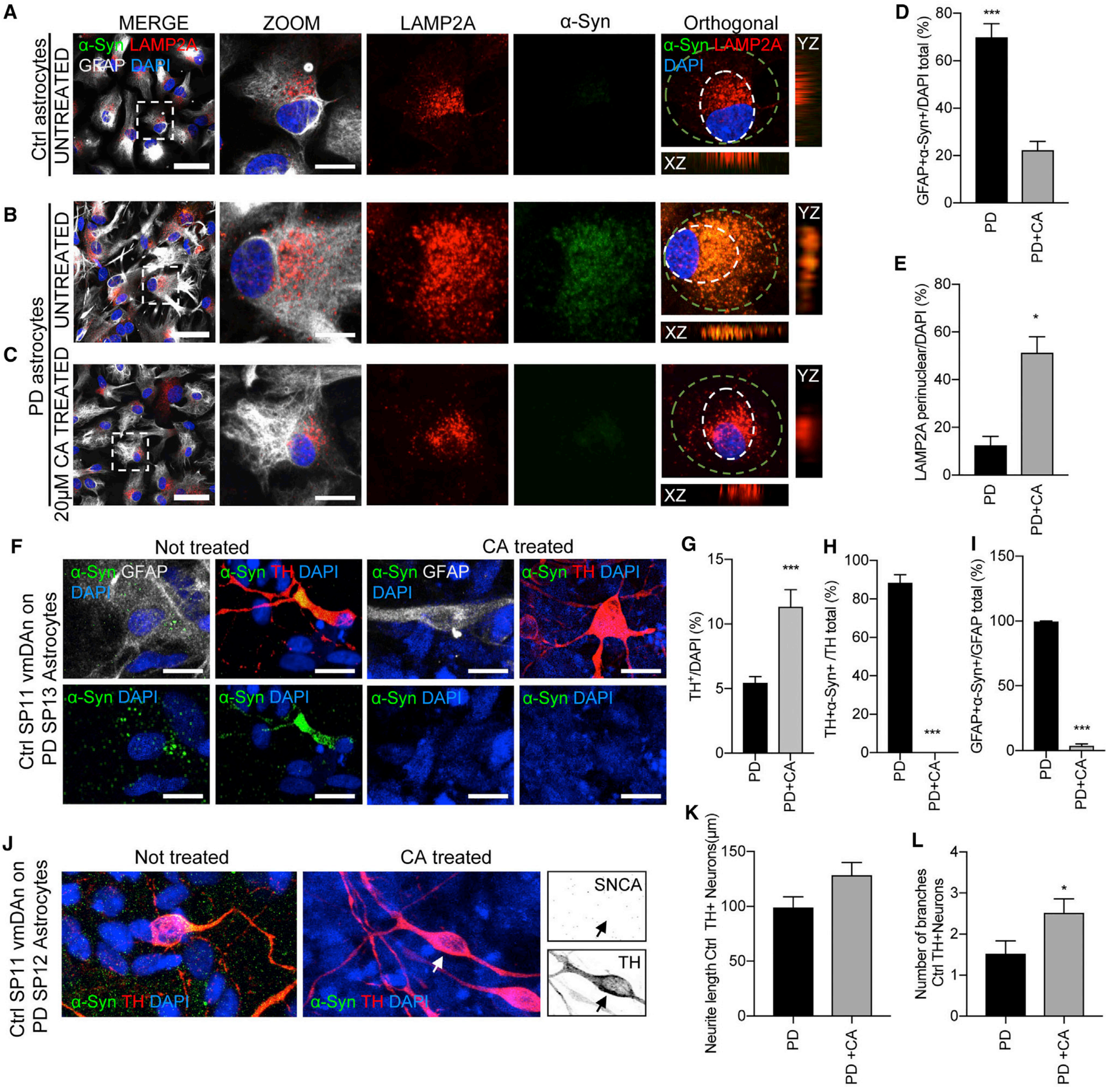

Figure 7. CMA Activator Drug Treatment Rescues α-syn Accumulation in Neurons Cultured with PD Astrocytes

(A and B) Representative ICC images of (A) Ctrl and (B) PD astrocytes after 2 weeks in culture without treatment stained for LAMP2A, α-syn, GFAP, and DAPI. Orthogonal views reveal positive co-localization of α-syn to LAMP2A in PD untreated sample. Images on the right show a magnification of the area boxed in the left images. Dashed circles outline the perinuclear area of the cell. Scale bars, 100 and 20 μm in merge and zoom, respectively.

(C) Representative ICC images of PD astrocytes after 20 μg of CA drug treatment stained for LAMP2A, α-syn, GFAP, and DAPI. Scale bars, 100 and 20 μm in merge and zoom, respectively.

(D and E) (D) Quantitative analysis of PD astrocytes (SP13 and SP12) stained positive for α-syn either not treated or treated with CA; (E) quantitative analysis of the same astrocytes under the same conditions, showing LAMP2A puncta in the perinuclear area (n = 3).

(F) Representative ICC images of 4-week Ctrl SP11 vmDAns co-cultured on PD SP13 astrocytes (left) or treated with CA for 2 weeks (right). Samples were stained for GFAP, TH, α-syn, and DAPI. Scale bars, 20 μm.

(legend continued on next page)

contribute to the altered α-syn proteostasis observed in PD astrocytes.

Restoration of α-syn Proteostasis in PD Astrocytes Alone and during Co-culture of Control Neurons with PD Astrocytes

Intracellular accumulation of α-syn has been shown to contribute to cellular toxicity in PD and to further disrupt functioning of cellular proteostasis systems (reviewed in Abeliovich and Gitler, 2016). We next investigated whether α-syn accumulation in PD astrocytes could be ameliorated by enhancing lysosomal activity. PD astrocytes were treated with a CMA activator (CA), which operates through the release of the endogenous inhibition of the retinoic receptor-α signaling pathway over CMA (Anguiano et al., 2013). Cells were treated with a concentration of 20 μM for 5 days and levels of α-syn were analyzed by immunofluorescence (Figure 7). LAMP2A-positive lysosomes in PD astrocytes treated with the CA (Figures 7C–7E) recovered the perinuclear distribution observed in Ctrl cells (Figure 7A) compared with when not treated (Figure 7B), suggesting reactivation of CMA in these cells. Consistent with higher CMA activity, CA-treated cells had significantly lower α-syn content than untreated cells (Figures 7C–7E).

In addition, we treated PD astrocytes when in co-culture with Ctrl neurons (Figure 7F). Under untreated conditions, Ctrl neurons accumulate astrocytic α-syn and show signs of neurodegeneration. Interestingly, the treatment with CA cleared out α-syn not only in astrocytes, but also in vmDAns, partially restored neuron survival, and decreased the number of TH-positive cells with a degenerative morphology (Figures 7G–7L). These findings suggest that although multiple protein degradation pathways fail to efficiently degrade α-syn in PD cells, reactivation of one of these pathways, in our case CMA, is able to restore functional α-syn proteostasis.

DISCUSSION

Astrocytes from three PD patients carrying the G2019S mutation in the *LRRK2* gene and two healthy individuals were successfully generated using a previously published protocol and fully characterized. By implementing a patient iPSC-based co-culture model, in this study we describe a role for PD astrocytes in midbrain neuronal cell death. Specifically, in a 4-week co-culture system, we found morphological alterations resembling those of neurodegeneration, such as short and few neurites as well as beaded necklace-like neurites, typically observed in neurons upon transport alterations (Fu et al., 2005; Garrido et al., 2011), and increased neuronal loss in Ctrl neurons co-cultured with PD astrocytes. We interpret these altered phenotypes as representing PD astrocyte-induced vmDAn neurodegeneration. An alternative explanation could be that PD astrocytes impinged on the differentiation and/or maturation of DAn progenitors in our iPSC-derived co-culture system. However, while we cannot formally rule out this possibility, two lines of evidence strongly argue against it playing a significant role in the phenotypes described here. First, we used vmDA neural differentiation cultures at 35 days of differentiation for our co-culture experiments. At this time, most vmDAns are already committed in fate (TH^+/$FOXA2^+$, see Figure 2A), but are still at a stage of maturation that does not compromise their survival upon cell dissociation and plating on top of the astrocyte cultures. Second, the numbers of vmDAns at different time points along the co-culture experiments showed progressive decline in co-cultures with PD astrocytes, but no significant changes when co-cultured with Ctrl astrocytes (Figure S2C). These results indicate that few new TH^+ neurons are generated during co-culture, and further support that the decreased numbers of vmDAns observed upon co-culture with PD astrocytes are a consequence of vmDAn degeneration. Importantly, the altered phenotypes were specifically observed in the subpopulation of midbrain dopaminergic neurons, as numbers of $MAP2^+$/TH^- neurons did not change significantly upon co-culture with Ctrl or PD astrocytes. In accordance with this, it has been already shown that α-syn toxicity was responsible for nigrostriatal neuronal cell death in midbrain cultures (Petrucelli et al., 2002), a relevant finding regarding the particular vulnerability of nigral neurons in PD. However, it remains to be tested whether, in prolonged culture, PD astrocytes also impair the survival of TH^- populations.

Postmortem brain tissue of PD patients revealed α-syn accumulation in astrocytes (Wakabayashi et al., 2000).

(G–I) (G) Quantitative analysis of the percentage of vmDAns remaining after being co-cultured with PD SP12 or PD SP13 (without treatment or treated with CA) for 4 weeks. Percentage of the (H) vmDAns or (I) astrocytes that stained positive for α-syn 4 weeks after the same co-culture conditions (n = 6).
(J) Representative ICC images of 4-week Ctrl SP11 vmDAns co-cultured on PD SP12 astrocytes with or without CA treatment for 2 weeks. Samples were stained for TH, α-syn, and DAPI. Arrows indicate the absence of α-syn accumulation in the selected TH-positive cell. Scale bars, 20 μm.
(K and L) (K) Neurite length quantification and (L) number of branches of 4-week Ctrl SP11 vmDAns when co-cultured on PD SP12 or PD SP13 astrocytes with or without CA treatment for 2 weeks (n = 4); 20 neurons counted per experiment.
Data are expressed as mean ± SEM, unpaired two-tailed Student's t test, $^*p<0.05$; $^{***}p < 0.001$.

It has been previously described that astrocytes accumulate neuronal-derived α-syn as a mechanism of neuroprotection (Booth et al., 2017). Indeed, in our study we found that Ctrl astrocytes accumulate α-syn when co-cultured with PD neurons and partially rescued the morphological phenotype of neurodegeneration and clearance of neuronal α-syn. This behavior suggests a neuroprotective effect via inflammatory-mediated activation of the Ctrl astrocytes. In addition, by using a CRISPR/Cas9 gene-edited cell line tagging the endogenous *SNCA* locus with an FLAG tag, our results reveal that PD astrocytes also accumulate and transfer α-syn to the surrounding neurons, suggesting that astrocytes actively contribute to the distribution of α-syn.

Taking into account that our PD astrocytes come from patients carrying the *LRRK2* G2019S mutation, we investigated whether disease-specific phenotypes related to the mutation were present. The α-syn accumulation in our co-culture system indicated a disruption in the way α-syn is usually degraded in PD astrocytes. Degradation of α-syn has been shown to occur by both proteasome and autophagic pathways, and conversely, high levels of α-syn have been demonstrated to be toxic for both systems (Tanaka et al., 2001; Webb et al., 2003; Winslow et al., 2010). Here we found that lysosomal degradation of α-syn was severely inhibited in PD astrocytes. We have previously described in PD neurons that one of the early events in the dysfunction of the proteostasis systems in these cells is the disruption of CMA by mutant LRRK2 binding to the LAMP2A, thus causing the accumulation of α-syn (Orenstein et al., 2013). Here we demonstrate that CMA is also altered in PD astrocytes and confirm that α-syn degradation by CMA in these cells was almost completely abolished.

The increase in intracellular levels of α-syn, due to its poor degradation in PD astrocytes by CMA, may contribute to precipitating malfunctioning of other proteostasis mechanisms, such as the proteasome and macroautophagy. In fact, we demonstrated that macroautophagy was also markedly impaired in these cells, by showing higher basal levels of autophagic vacuoles (LC3-II) and the autophagic cargo p62, and reduced autophagic flux (for both LC3-II and p62). The lower co-localization between the autophagosomal and lysosomal markers observed in PD astrocytes suggests that the reduced autophagic flux is due to a defect in autophagosome/lysosome fusion, similar to that previously described in PD neurons.

Taking into account the coordinate functioning of the proteolytic systems, and the fact that CMA disruption seems to occur early during the development of PD pathology, we attempted to restore normal α-syn proteostasis by enhancing CMA activity. Our findings in cells treated with a chemical activator of CMA suggest that upregulation of CMA is still possible in these cells and that this intervention is sufficient to return levels of α-syn close to those in Ctrl cells. Although α-syn was cleared, restoration during a co-culture with Ctrl neurons was only partial in terms of neurite length and number, suggesting that the neurodegeneration observed could also be due to other non-α-syn-related factors secreted by PD astrocytes.

Overall, our findings propose a specific role for astrocytes in mediating dopaminergic cell death during PD. PD-specific phenotypes specifically related to dysfunctions in the pathways of protein degradation have been observed in PD astrocytes and not in Ctrl astrocytes. Dysfunctional CMA, progressive α-syn accumulation, and glia-to-neuron transfer found in our PD astrocytes are all aspects that can compromise neuronal survival during PD pathogenesis. Future studies will identify whether additional factors other than α-syn are being secreted by (or lacking in) PD astrocytes, and thus contributing to triggering vmDA neuronal cell death. iPSC-based technology allows for the proper recapitulation of patient-specific disease-related phenotypes, which will aid in the discovery of new therapies.

EXPERIMENTAL PROCEDURES

Experimental procedures are also provided in Supplemental Information.

iPSC-Derived Astrocyte Generation and Culture

The parental iPSC lines used in our studies were previously generated and fully characterized (Sanchez-Danes et al., 2012). The generation and use of human iPSCs in this work were approved by the Spanish competent authorities (Commission on Guarantees concerning the Donation and Use of Human Tissues and Cells of the Carlos III National Institute of Health). iPSCs were differentiated into astrocytes following a previously published protocol (Serio et al., 2013). See Supplemental Information for more details.

iPSC-Derived vmDAn Generation

Four different iPSCs, two PD (SP12 and SP13) and two Ctrl (SP11 and SP11#4), were differentiated into dopaminergic neurons using a combination of two previously published protocols for midbrain induction (Chambers et al., 2009; Kriks et al., 2011). Detailed methods are provided in Supplemental Information.

ICC

ICC on cell cultures was performed as described in Supplemental Information.

Statistical Analysis

Statistical analyses of the obtained data were performed using two-tailed unequal variance Student's t tests and ANOVA (*$p < 0.05$, **$p < 0.01$, ***$p < 0.001$), and the mean and standard error of the mean were plotted using Prism (Mac OS X). Number of independent experiments (n) is indicated in each figure legend.

SUPPLEMENTAL INFORMATION

Supplemental Information includes Supplemental Experimental Procedures, seven figures, and three tables and can be found

with this article online at https://doi.org/10.1016/j.stemcr.2018.12.011.

AUTHOR CONTRIBUTIONS

Conceptualization, A.C. and A.R.; Methodology, I.F., J.P.M., A.Z., A.M.C., and J.S.; Investigation, Y.R.P, A.D., G.C., C.C., M.P.-E., M.G., I.F.-C., J.P., and A.F.; Validation, A.C. and A.R.; Writing – Original Draft, A.D.; Writing – Review & Editing, A.C. and A.R.; Visualization, A.D.; Resources, A.Z., A.M.C., J.S., E.T., A.C., and A.R.; Funding Acquisition, A.C.; Supervision, A.C.

ACKNOWLEDGMENTS

The authors are indebted to the patients with PD who have participated in this study. The authors thank Chrysanthi Blithikioti for helping with some co-culture experiments and ICC, Neus Bayó-Puxan for her advice on western blotting, Jose Miquel Andres Vaquero (CMRB) for performing flow cytometry analysis, and David Maynar for excellent artwork. We are thankful to Mark Cookson for the LRRK2 G2019S plasmid (Addgene Plasmid No. 29401). We are grateful to the Advanced Fluorescence Microscopy Unit of the Institute of Biomedicine of the University of Barcelona (especially to Elena Rebollo Arredondo). Research from the authors' laboratories is supported by the European Research Council (ERC) (2012-StG-311736-PD-HUMMODEL), the Spanish Ministry of Economy and Competitiveness (MINECO) (FIS2016-78507-C2-2-P, SAF2015-69706-R, and BFU2016-80870-P), Instituto de Salud Carlos III (ISCIII/FEDER) (Red de Terapia Celular [TerCel] RD16/0011/0024), AGAUR (2014-SGR-878 and 2017-SGR-899), and CERCA Program/Generalitat de Catalunya. A.D. is supported by the PD-HUMMODEL ERC-Ideas PhD fellowship. C.C. and G.C. are partially supported by predoctoral fellowships from the Spanish Ministry of Education (MEC) (FPU12/03332) and MINECO (BES-2014-069603), respectively.

Received: April 7, 2018
Revised: December 11, 2018
Accepted: December 13, 2018
Published: January 10, 2019

SUPPORTING CITATIONS

The following references appear in the Supplemental Information: Alwin et al., 2005; Kita-Matsuo et al., 2009; Massey et al., 2008; Mussolino et al., 2011; Park et al., 2015.

REFERENCES

Abeliovich, A., and Gitler, A.D. (2016). Defects in trafficking bridge Parkinson's disease pathology and genetics. Nature *539*, 207–216.

Alwin, S., Gere, M.B., Guhl, E., Effertz, K., Barbas, C.F., 3rd, Segal, D.J., Weitzman, M.D., and Cathomen, T. (2005). Custom zinc-finger nucleases for use in human cells. Mol. Ther. *12*, 610–617.

Anguiano, J., Garner, T.P., Mahalingam, M., Das, B.C., Gavathiotis, E., and Cuervo, A.M. (2013). Chemical modulation of chaperone-mediated autophagy by retinoic acid derivatives. Nat. Chem. Biol. *9*, 374–382.

Booth, H.D.E., Hirst, W.D., and Wade-Martins, R. (2017). The role of astrocyte dysfunction in Parkinson's disease pathogenesis. Trends Neurosci. *40*, 358–370.

Braak, H., Sastre, M., and Del Tredici, K. (2007). Development of alpha-synuclein immunoreactive astrocytes in the forebrain parallels stages of intraneuronal pathology in sporadic Parkinson's disease. Acta Neuropathol. *114*, 231–241.

Cavaliere, F., Cerf, L., Dehay, B., Ramos-Gonzalez, P., De Giorgi, F., Bourdenx, M., Bessede, A., Obeso, J.A., Matute, C., Ichas, F., et al. (2017). In vitro alpha-synuclein neurotoxicity and spreading among neurons and astrocytes using Lewy body extracts from Parkinson disease brains. Neurobiol. Dis. *103*, 101–112.

Chambers, S.M., Fasano, C.A., Papapetrou, E.P., Tomishima, M., Sadelain, M., and Studer, L. (2009). Highly efficient neural conversion of human ES and iPS cells by dual inhibition of SMAD signaling. Nat. Biotechnol. *27*, 275–280.

Cookson, M.R. (2016). Cellular functions of LRRK2 implicate vesicular trafficking pathways in Parkinson's disease. Biochem. Soc. Trans. *44*, 1603–1610.

Cookson, M.R. (2017). Mechanisms of mutant LRRK2 neurodegeneration. Adv. Neurobiol. *14*, 227–239.

Cuervo, A.M., Stefanis, L., Fredenburg, R., Lansbury, P.T., and Sulzer, D. (2004). Impaired degradation of mutant alpha-synuclein by chaperone-mediated autophagy. Science *305*, 1292–1295.

Fu, L., Gao, Y.S., Tousson, A., Shah, A., Chen, T.L., Vertel, B.M., and Sztul, E. (2005). Nuclear aggresomes form by fusion of PML-associated aggregates. Mol. Biol. Cell *16*, 4905–4917.

Garrido, M., Tereshchenko, Y., Zhevtsova, Z., Taschenberger, G., Bahr, M., and Kugler, S. (2011). Glutathione depletion and overproduction both initiate degeneration of nigral dopaminergic neurons. Acta Neuropathol. *121*, 475–485.

Gilks, W.P., Abou-Sleiman, P.M., Gandhi, S., Jain, S., Singleton, A., Lees, A.J., Shaw, K., Bhatia, K.P., Bonifati, V., Quinn, N.P., et al. (2005). A common LRRK2 mutation in idiopathic Parkinson's disease. Lancet *365*, 415–416.

Global Burden of Disease Study Collaborators. (2015). Global, regional, and national incidence, prevalence, and years lived with disability for 301 acute and chronic diseases and injuries in 188 countries, 1990–2013: a systematic analysis for the Global Burden of Disease Study 2013. Lancet *386*, 743–800.

Greenamyre, J.T., and Hastings, T.G. (2004). Biomedicine. Parkinson's—divergent causes, convergent mechanisms. Science *304*, 1120–1122.

Gu, X.L., Long, C.X., Sun, L., Xie, C., Lin, X., and Cai, H. (2010). Astrocytic expression of Parkinson's disease-related A53T alpha-synuclein causes neurodegeneration in mice. Mol. Brain *3*, 12.

Heman-Ackah, S.M., Manzano, R., Hoozemans, J.J.M., Scheper, W., Flynn, R., Haerty, W., Cowley, S.A., Bassett, A.R., and Wood, M.J.A. (2017). Alpha-synuclein induces the unfolded protein response in Parkinson's disease SNCA triplication iPSC-derived neurons. Hum. Mol. Genet. *26*, 4441–4450.

Kiffin, R., Christian, C., Knecht, E., and Cuervo, A.M. (2004). Activation of chaperone-mediated autophagy during oxidative stress. Mol. Biol. Cell *15*, 4829–4840.

Kita-Matsuo, H., Barcova, M., Prigozhina, N., Salomonis, N., Wei, K., Jacot, J.G., Nelson, B., Spiering, S., Haverslag, R., Kim, C., et al. (2009). Lentiviral vectors and protocols for creation of stable hESC lines for fluorescent tracking and drug resistance selection of cardiomyocytes. PLoS One *4*, e5046.

Koga, H., Martinez-Vicente, M., Macian, F., Verkhusha, V.V., and Cuervo, A.M. (2011). A photoconvertible fluorescent reporter to track chaperone-mediated autophagy. Nat. Commun. *2*, 386.

Kriks, S., Shim, J.W., Piao, J., Ganat, Y.M., Wakeman, D.R., Xie, Z., Carrillo-Reid, L., Auyeung, G., Antonacci, C., Buch, A., et al. (2011). Dopamine neurons derived from human ES cells efficiently engraft in animal models of Parkinson's disease. Nature *480*, 547–551.

Lee, H.J., Suk, J.E., Patrick, C., Bae, E.J., Cho, J.H., Rho, S., Hwang, D., Masliah, E., and Lee, S.J. (2010). Direct transfer of alpha-synuclein from neuron to astroglia causes inflammatory responses in synucleinopathies. J. Biol. Chem. *285*, 9262–9272.

Lill, C.M. (2016). Genetics of Parkinson's disease. Mol. Cell Probes *30*, 386–396.

Martinez-Vicente, M., Talloczy, Z., Kaushik, S., Massey, A.C., Mazzulli, J., Mosharov, E.V., Hodara, R., Fredenburg, R., Wu, D.C., Follenzi, A., et al. (2008). Dopamine-modified alpha-synuclein blocks chaperone-mediated autophagy. J. Clin. Invest. *118*, 777–788.

Massey, A.C., Follenzi, A., Kiffin, R., Zhang, C., and Cuervo, A.M. (2008). Early cellular changes after blockage of chaperone-mediated autophagy. Autophagy *4*, 442–456.

Massey, A.C., Kaushik, S., Sovak, G., Kiffin, R., and Cuervo, A.M. (2006). Consequences of the selective blockage of chaperone-mediated autophagy. Proc. Natl. Acad. Sci. U S A *103*, 5805–5810.

Mussolino, C., Morbitzer, R., Lutge, F., Dannemann, N., Lahaye, T., and Cathomen, T. (2011). A novel TALE nuclease scaffold enables high genome editing activity in combination with low toxicity. Nucleic Acids Res. *39*, 9283–9293.

Nguyen, H.N., Byers, B., Cord, B., Shcheglovitov, A., Byrne, J., Gujar, P., Kee, K., Schule, B., Dolmetsch, R.E., Langston, W., et al. (2011). LRRK2 mutant iPSC-derived DA neurons demonstrate increased susceptibility to oxidative stress. Cell Stem Cell *8*, 267–280.

Nichols, W.C., Pankratz, N., Hernandez, D., Paisan-Ruiz, C., Jain, S., Halter, C.A., Michaels, V.E., Reed, T., Rudolph, A., Shults, C.W., et al. (2005). Genetic screening for a single common LRRK2 mutation in familial Parkinson's disease. Lancet *365*, 410–412.

Orenstein, S.J., Kuo, S.H., Tasset, I., Arias, E., Koga, H., Fernandez-Carasa, I., Cortes, E., Honig, L.S., Dauer, W., Consiglio, A., et al. (2013). Interplay of LRRK2 with chaperone-mediated autophagy. Nat. Neurosci. *16*, 394–406.

Park, C., Suh, Y., and Cuervo, A.M. (2015). Regulated degradation of Chk1 by chaperone-mediated autophagy in response to DNA damage. Nat. Commun. *6*, 6823.

Petrucelli, L., O'Farrell, C., Lockhart, P.J., Baptista, M., Kehoe, K., Vink, L., Choi, P., Wolozin, B., Farrer, M., Hardy, J., et al. (2002). Parkin protects against the toxicity associated with mutant alpha-synuclein: proteasome dysfunction selectively affects catecholaminergic neurons. Neuron *36*, 1007–1019.

Sanchez-Danes, A., Richaud-Patin, Y., Carballo-Carbajal, I., Jimenez-Delgado, S., Caig, C., Mora, S., Di Guglielmo, C., Ezquerra, M., Patel, B., Giralt, A., et al. (2012). Disease-specific phenotypes in dopamine neurons from human iPS-based models of genetic and sporadic Parkinson's disease. EMBO Mol. Med. *4*, 380–395.

Schneider, J.L., Villarroya, J., Diaz-Carretero, A., Patel, B., Urbanska, A.M., Thi, M.M., Villarroya, F., Santambrogio, L., and Cuervo, A.M. (2015). Loss of hepatic chaperone-mediated autophagy accelerates proteostasis failure in aging. Aging Cell *14*, 249–264.

Serio, A., Bilican, B., Barmada, S.J., Ando, D.M., Zhao, C., Siller, R., Burr, K., Haghi, G., Story, D., Nishimura, A.L., et al. (2013). Astrocyte pathology and the absence of non-cell autonomy in an induced pluripotent stem cell model of TDP-43 proteinopathy. Proc. Natl. Acad. Sci. U S A *110*, 4697–4702.

Skibinski, G., Nakamura, K., Cookson, M.R., and Finkbeiner, S. (2014). Mutant LRRK2 toxicity in neurons depends on LRRK2 levels and synuclein but not kinase activity or inclusion bodies. J. Neurosci. *34*, 418–433.

Su, Y.C., Guo, X., and Qi, X. (2015). Threonine 56 phosphorylation of Bcl-2 is required for LRRK2 G2019S-induced mitochondrial depolarization and autophagy. Biochim. Biophys. Acta *1852*, 12–21.

Tanaka, Y., Engelender, S., Igarashi, S., Rao, R.K., Wanner, T., Tanzi, R.E., Sawa, A., L Dawson, V., Dawson, T.M., and Ross, C.A. (2001). Inducible expression of mutant alpha-synuclein decreases proteasome activity and increases sensitivity to mitochondria-dependent apoptosis. Hum. Mol. Genet. *10*, 919–926.

Wakabayashi, K., Hayashi, S., Yoshimoto, M., Kudo, H., and Takahashi, H. (2000). NACP/alpha-synuclein-positive filamentous inclusions in astrocytes and oligodendrocytes of Parkinson's disease brains. Acta Neuropathol. *99*, 14–20.

Webb, J.L., Ravikumar, B., Atkins, J., Skepper, J.N., and Rubinsztein, D.C. (2003). Alpha-Synuclein is degraded by both autophagy and the proteasome. J. Biol. Chem. *278*, 25009–25013.

Winslow, A.R., Chen, C.W., Corrochano, S., Acevedo-Arozena, A., Gordon, D.E., Peden, A.A., Lichtenberg, M., Menzies, F.M., Ravikumar, B., Imarisio, S., et al. (2010). alpha-Synuclein impairs macroautophagy: implications for Parkinson's disease. J. Cell Biol. *190*, 1023–1037.

Zeltner, N., and Studer, L. (2015). Pluripotent stem cell-based disease modeling: current hurdles and future promise. Curr. Opin. Cell Biol. *37*, 102–110.

Stem Cell Reports, Volume *12*

Supplemental Information

Patient-Specific iPSC-Derived Astrocytes Contribute to Non-Cell-Autonomous Neurodegeneration in Parkinson's Disease

Angelique di Domenico, Giulia Carola, Carles Calatayud, Meritxell Pons-Espinal, Juan Pablo Muñoz, Yvonne Richaud-Patin, Irene Fernandez-Carasa, Marta Gut, Armida Faella, Janani Parameswaran, Jordi Soriano, Isidro Ferrer, Eduardo Tolosa, Antonio Zorzano, Ana Maria Cuervo, Angel Raya, and Antonella Consiglio

Supplementary Information

Patient-specific iPSC-derived astrocytes contribute to non-cell autonomous neurodegeneration in Parkinson's disease

Angelique di Domenico,[1,2,14] Giulia Carola,[1,2,14] Carles Calatayud,[1,2,3] Meritxell Pons-Espinal,[1,2] Juan Pablo Muñoz,[4] Yvonne Richaud-Patin,[3,5] Irene Fernandez-Carasa,[1,2] Marta Gut,[6] Armida Faella,[1,2] Janani Parameswaran,[1,2] Jordi Soriano,[7,8] Isidro Ferrer,[2,9] Eduardo Tolosa,[9,10] Antonio Zorzano,[4] Ana Maria Cuervo,[11] Angel Raya, [3,5,12,*] Antonella Consiglio[1,2,13,*,‡]

[1] Department of Pathology and Experimental Therapeutics, Bellvitge University Hospital-IDIBELL, 08908 Hospitalet de Llobregat, Spain.

[2] Institute of Biomedicine of the University of Barcelona (IBUB), Barcelona 08028, Spain.

[3] Center of Regenerative Medicine in Barcelona (CMRB), Hospital Duran i Reynals, Hospitalet de Llobregat, 08908 Barcelona, Spain.

[4] Institute for Research in Biomedicine (IRB), Barcelona 08028, Spain

[5] Centre for Networked Biomedical Research on Bioengineering, Biomaterials and Nanomedicine (CIBER-BBN), Madrid 28029, Spain.

[6] Centre Nacional d'Anàlisi Genòmica (CNAG-CRG), Parc Científic de Barcelona, Barcelona 08028, Spain.

[7] Departament d'Estructura i Constituents de la Matèria, Universitat de Barcelona, Barcelona 08028, Spain.

[8] Universitat de Barcelona Institute of Complex Systems (UBICS), Barcelona 08028, Spain.

[9] Centre for Networked Biomedical Research on Neurodegenerative Diseases (CIBERNED), Madrid 28049, Spain.

[10] Department of Neurology, Hospital Clínic de Barcelona, Institut d'Investigacions Biomèdiques August Pi i Sunyer (IDIBAPS), University of Barcelona (UB), Barcelona 08036, Spain.

[11] Albert Einstein College of Medicine, 300 Morris Park Ave, Bronx, NY 10461, United States

[12] Institució Catalana de Recerca i Estudis Avançats (ICREA), Barcelona 08010, Spain

[13] Department of Molecular and Translational Medicine, University of Brescia, Brescia 25121, Italy

[14] Co-first author

* Correspondence should be addressed to araya@cmrb.eu (A.R.) OR consiglio@ub.edu (A.C.)

‡ Lead contact

*Equal contribution

Supplementary Information contains:

3 Supplementary Tables:

Table S1. Patient information used in the study. Healthy donors are referred to: SP09, SP11 and SP17. *LRRK2* G2019S are referred to: SP06, SP12 and SP13. Related to Figure 1.

Table S2. Summary of the characterization of iPSC lines used in this study. Related to Table 1 and all the Figures.

Table S3. Summary of phenotypes observed in the co-cultures experiments. Duration of the co-culture, neurons and astrocytes viability, α-syn accumulation and cell morphology are described in all conditions tested. Related to Figure 3 and 4.

7 Supplementary Figures

Fig. S1. iPSC derived astrocyte characterization. Related to Fig. 1.

Fig. S2. Ctrl vmDAn neuronal morphology upon co-cultured with PD astrocytes and viability analysis and CRISPR/Cas9 gene editing strategy tagging *SNCA* locus with FLAG tag. Related to Fig. 3.

Fig. S3. Effects of Ctrl and PD/PD-FLAG astrocyte conditioned medium on α-synuclein accumulation in Ctrl vmDA neurons. Related to Fig. 3 and 4.

Fig. S4. Effect of *LRRK2* G2019S transfected in control astrocytes. Related to Fig. 3.

Fig. S5. Schematic description of the generation of isogenic control line and phenotypic comparison between isogenic clones, alone or in co-culture with Ctrl-SP11 neurons. Related to Fig. 3 and 4.

Fig. S6. Altered CMA, dysfunctional macroautophagy and α-syn accumulation in PD astrocytes. Related to Fig. 5. and 6.

Fig. S7. Original western blots. Related to Fig. 5 and 6.

Supplementary Experimental Procedures

Table S1. Patient Information

Subject Identifier (**SP**)	*LRRK2* G2019S Patients			Healthy Donors		
	**SP12**	**SP13**	**SP06**	**SP09**	**SP17**	**SP11**
**Age at donation**	63	68	44	66	52	48
**Age at diagnosis**	50	57	33	N/A	N/A	N/A
**Gender**	Female	Female	Male	Male	Male	Female
**Ethnicity**	Caucasian	Caucasian	Caucasian	Caucasian	Caucasian	Caucasian
**Mutation**	G2019S	G2019S	G2019S	No	No	No
**PD diagnosis**	Criteria - fPD	Criteria - fPD	Criteria - fPD	No	No	No
**Family history**	Yes	Yes	Yes	N/A	N/A	N/A
**Unilateral onset**	Yes	Yes	Yes	N/A	N/A	N/A
**Resting tremor**	Yes	Yes	Yes	N/A	N/A	N/A
**Progressive disease**	Yes	Yes	Yes	N/A	N/A	N/A
**L-dopa responsive**	Yes	Yes	Yes	N/A	N/A	N/A
**L-dopa responsive >5yrs**	Yes	Yes	Yes	N/A	N/A	N/A
**L-dopa induced Chorea**	Yes	No	Yes	N/A	N/A	N/A
**Clinical course >10yrs**	Yes	Yes	Yes	N/A	N/A	N/A
**Head trauma**	No	No	No	N/A	N/A	N/A
**Dementia**	No	No	No	N/A	N/A	N/A
**Deep brain stimulation**	No	No	Yes	N/A	N/A	N/A
**Others**	Non-smoker. No alcohol consumption	Coffee drinker. Non-smoker. No alcohol consumption. No anti-inflammatory treatment	Coffee drinker. Smoker. No anti-inflammatory treatment	N/A	N/A	N/A
**Treatment**	Neupro 12mg, Pantoprazol 40mg, Mirapexin, Hidroferol, Sinemet Plus, Hidroclorotiazida	Digoxina, BoiK, Dinisor Retard, Mirapexin, Omeprazol, Sinemet Plus, Sintrom	Stalevo, amantadine, Rivotril, amitriptilina	N/A	N/A	N/A

*fPD = familial PD

Table S2. Summary of the characterization of iPSC lines used in this study. Related to Table 1 and all Figures

iPSC line	Reprogramming method	# iPSC clone generated	Morphology	Pluripotency gene expression[1]	Pluripotency protein expression[2]	Three germ layers[3]	Karyotype	Reference	# astrocyte generation
**Ctrl SP09**	Retrovirus	4	+	+	+	+	Normal	Sanchez Danes (EMBO Mol Med. 2012)	3
**Ctrl SP11**	Retrovirus	4	+	+	+	+	Normal	Sanchez Danes (EMBO Mol Med. 2012)	3
**Ctrl SP11#4**	Retrovirus	4	+	+	+	+	Normal	Sanchez [illegible]es (EMBO Mol Med. 2012)	3
**Ctrl SP11- α-syn-FLAG**	N/A	3	+	+	+	+	Normal	This study	2
**Ctrl SP17**	Retrovirus	4	+	+	+	+	Normal	Sanchez Danes (EMBO Mol Med. 2012)	3
**PD SP06**	Retrovirus	4	+	+	+	+	Normal	Sanchez Danes (EMBO Mol Med. 2012)	3
**PD SP12**	Retrovirus	4	+	+	+	+	Normal	Sanchez Danes (EMBO Mol Med. 2012)	3
**PD SP12-α-syn-FLAG**	N/A	3	+	+	+	+	Normal	This study	2
**PD SP13**	Retrovirus	4	+	+	+	+	Normal	Sanchez Danes (EMBO Mol Med. 2012)	3
**PD Iso**	N/A	3	+	+	+	+	Normal	This study	2

[1]Expression levels of *OCT4*, *SOX2*, *NANOG* and *LIN28* by qRT-PCR

[2]Expression of OCT4, NANOG, TRA 1-81 and SSEA4 by immunocytochemistry

[3]Expression of AFP (endoderm), SMA (mesoderm) and TUJ1 (ectoderm) by immunocytochemistry

Table S3. Summary of Phenotypes During Co-cultures

Co-culture		Phenotypes**										
							**Cell Morphology**					
**Line**		**Duration**	**Cell viability** (fold change*)		**Alpha-synuclein accumulation** (%/total cell type)		Neuron				Astrocyte	
Neuron	Astrocyte	weeks	Neuron	Astrocyte	Neuron	Astrocyte	Status	Average neurite intersection #/neuron	Average neurite length/neuron		Resting (flat, large)	Reactive (retracted)
SP11	SP09	2	1	1	0	0	immature	N/A	N/A		100%	0
	SP09	4	1	1	0	0	normal	10	304 microns		100%	0
	SP17	4	1	1	0	0	normal	10	311 microns		100%	0
	isoSP13	4	0,5	5,6	7%	38%	degenerated	1	112 microns		100%	0
	SP11 α-syn-FLAG	4	1	1	0	0	normal	10	302 microns		100%	0
	SP06	4	0,4	3	46%	81%	degenerated	1	80,3 microns		0	100%
	SP13	2	0,5	1	0	100%	immature	N/A	N/A		0	100%
	SP13	4	0,2	1,1	45%	100%	degenerated	2	108 microns		0	100%
	SP12	4	0,2	1,6	59%	100%	degenerated	2	101 microns		0	100%
	SP12 α-syn-FLAG	4	0,3	1,5	57%	100%	degenerated	2	103 microns		0	100%
	pDEST51-LRRK2-SP09	4	0,5	1,4	50%	100%	degenerated	N/A	N/A		0	100%
	pDEST51-LRRK2-SP17	4	0,6	1,6	53%	100%	degenerated	N/A	N/A		0	100%
SP11#4	SP06	4	0,2	0,6	100%	94%	degenerated	1,5	138 microns		0	100%
	SP13	4	0,1	0,3	97%	72%	degenerated	1	96 microns		0	100%
SP12	SP09	2	1	1	0	0	immature	N/A	N/A		100%	0
	SP09	4	1,5	1	0	18%	normal	5	155 microns		82%	18%
	SP11 α-syn-FLAG	4	1,5	1	0	12%	normal	5	162 microns		88%	12%
	SP13	2	0,5	1,2	100%	100%	immature	N/A	N/A		0	100%
	SP13	4	0,2	1,5	100%	100%	degenerated	2	88 microns		0	100%
	SP12	4	0,3	1,5	100%	100%	degenerated	2	85 microns		0	100%
	SP12 α-syn-FLAG	4	0,2	1,5	100%	100%	degenerated	2	91 microns		0	100%
SP13	isoSP13	4	0,2	8,4	5%	60%	degenerated	1	127,5 microns		100%	0

* cell number normalized to average of both Ctrl astrocytes (SP09 and SP17) per condition

** average of all independent tripicates

Ctrl (control)

PD (mutant)

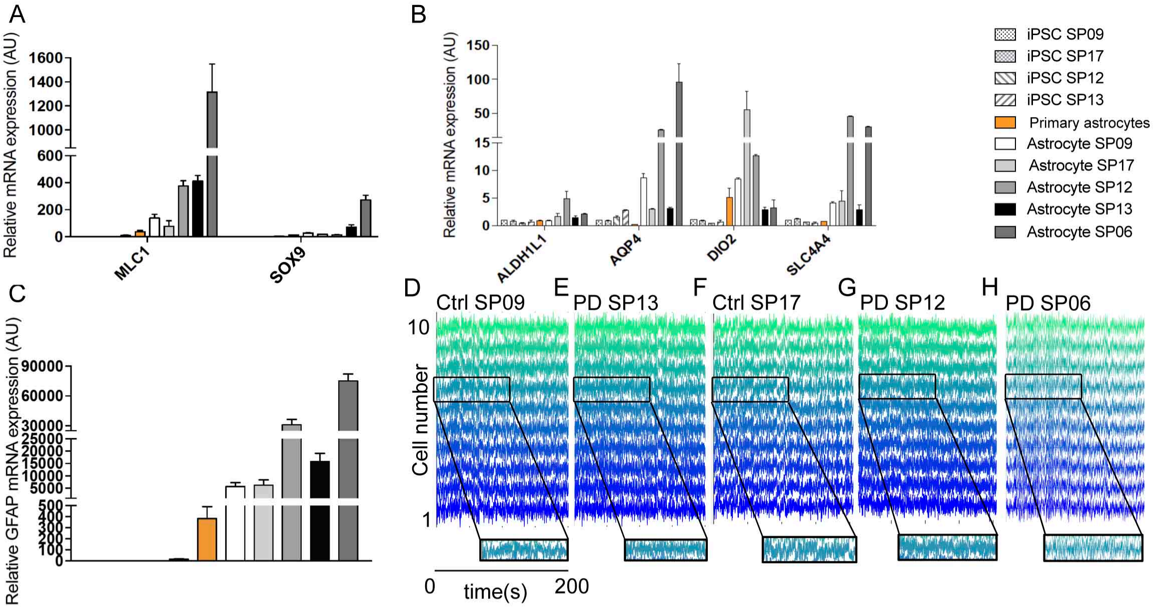

Figure S1. iPSC-derived astrocyte characterization.

(A-C) qRT-PCR analysis revealing comparable levels of *MLC1, SOX9, ALDH1L1, AQP4, DIO2, SLC4A4, GFAP* mRNA expression in Ctrl (SP09 and SP17) and PD (SP12, SP13 and SP06) iPSC-derived astrocytes, compared to human primary astrocytes and their corresponding iPSC.

(D-H) Graph representing single functional astrocyte calcium waves of Ctrl (SP09 and SP17) and PD (SP12, SP13 and SP06) astrocytes (n=3).

Data are expressed as mean ± s.e.m, unpaired two-tailed Student's t-test, *p<0.05, **p<0.01, ***p<0.001.

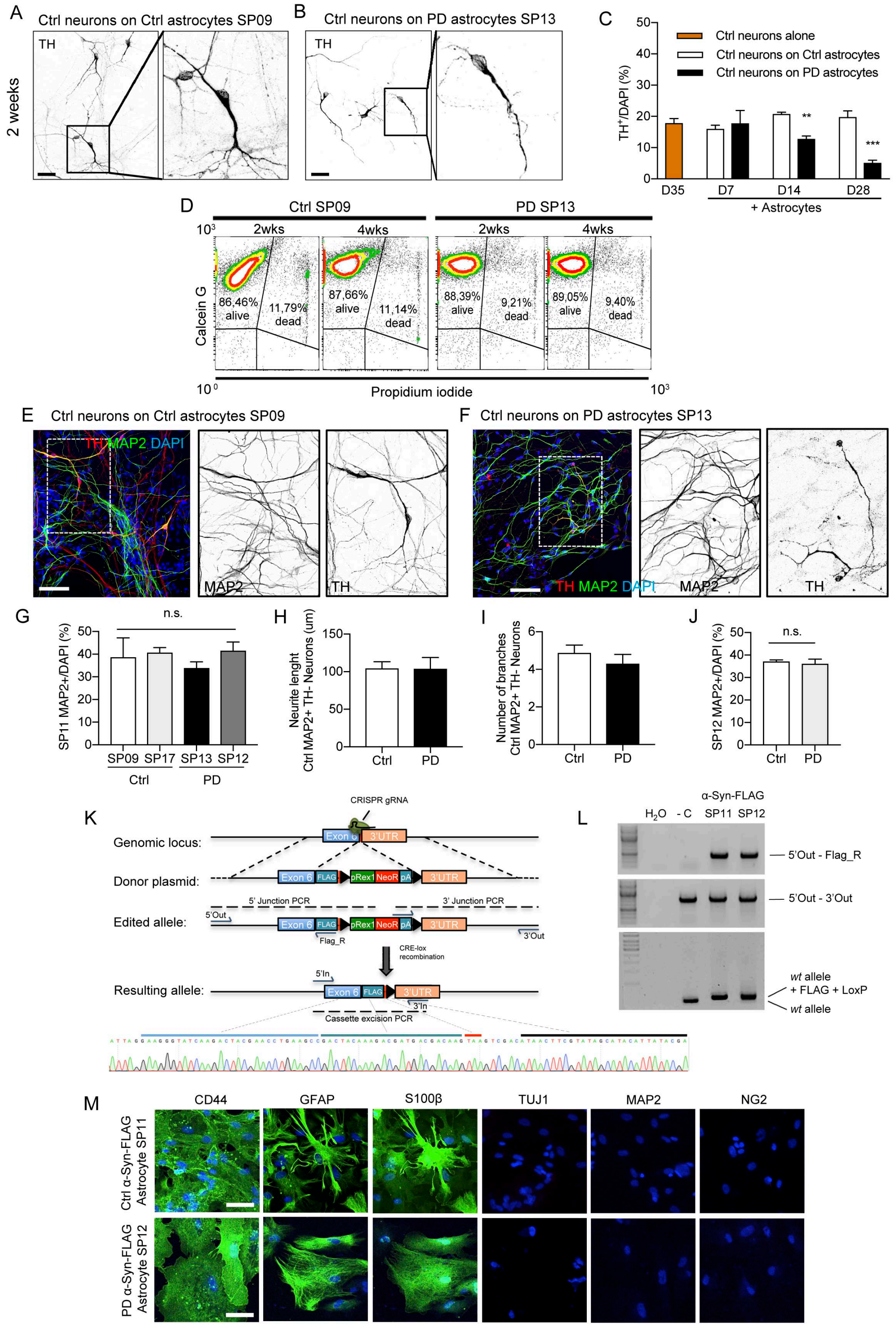

Figure S2. Ctrl vmDAn neuronal morphology upon co-cultured with PD astrocytes and viability analysis and CRISPR/Cas9 gene editing strategy tagging *SNCA* locus with FLAG tag.

(A-B) Representative images staining Ctrl SP11 vmDAn (TH, in black) on the top of Ctrl SP09 astrocytes (A) or PD SP13 astrocytes (B) during a 2-week co-culture period. Scale bar 20µm.

(C) Percentage of TH+/DAPI Ctrl SP11 neurons alone (orange bar) or when co-cultured with Ctrl SP09,

Ctrl SP17 or PD SP12, PD SP13 astrocytes after 7, 14 or 28 days of co-culture (n=3).
(D) Contour plot of Ctrl SP09 and PD SP13 astrocyte viability at 2 and 4 weeks.
(E-F) Representative image showing Ctrl SP11 vmDAn on the top of Ctrl SP09 (E) astrocytes or PD SP13 (F) astrocytes during a 4-week co-culture period. Samples were stained for TH, MAP2, and DAPI. Scale bar 20μm.
(G) Quantitative analysis of Ctrl SP11 neurons (MAP2 positive) remaining after 4 weeks in co-culture with Ctrl SP09, Ctrl SP17, PD SP12, or PD SP13 astrocytes (n=3).
(H-I) Quantification of the neurite length (H) and number of branches (I) of MAP2 positive TH negative neurons after 4 weeks of co-culture (n=3); total neurons counted per experiment n=30.
(J) Quantitative analysis of PD SP12 neurons (MAP2 positive) remaining after 4 weeks in co-culture with Ctrl SP09 or PD SP13 astrocytes (n=3).
Data are expressed as mean ± s.e.m, unpaired two-tailed Student's t-test, ***$p<0.001$.
(K) Scheme of the knock-in strategy of a FLAG-tag into the C terminus of the endogenous *SNCA* gene using CRISPR/Cas9. Blue arrows represent the primers used for the PCR screening procedure. Black triangles represent LoxP sites surrounding the selection cassette.
(L) PCR analysis of α-syn-FLAG knock-in iPSC lines Ctrl SP11 and PD SP12.
(M) Representative images of Ctrl SP11 and PD SP12 α-syn-FLAG astrocytes stained positive for CD44, GFAP, S100β and negative for TUJ1, MAP2, NG2. Scale bar 100μm.

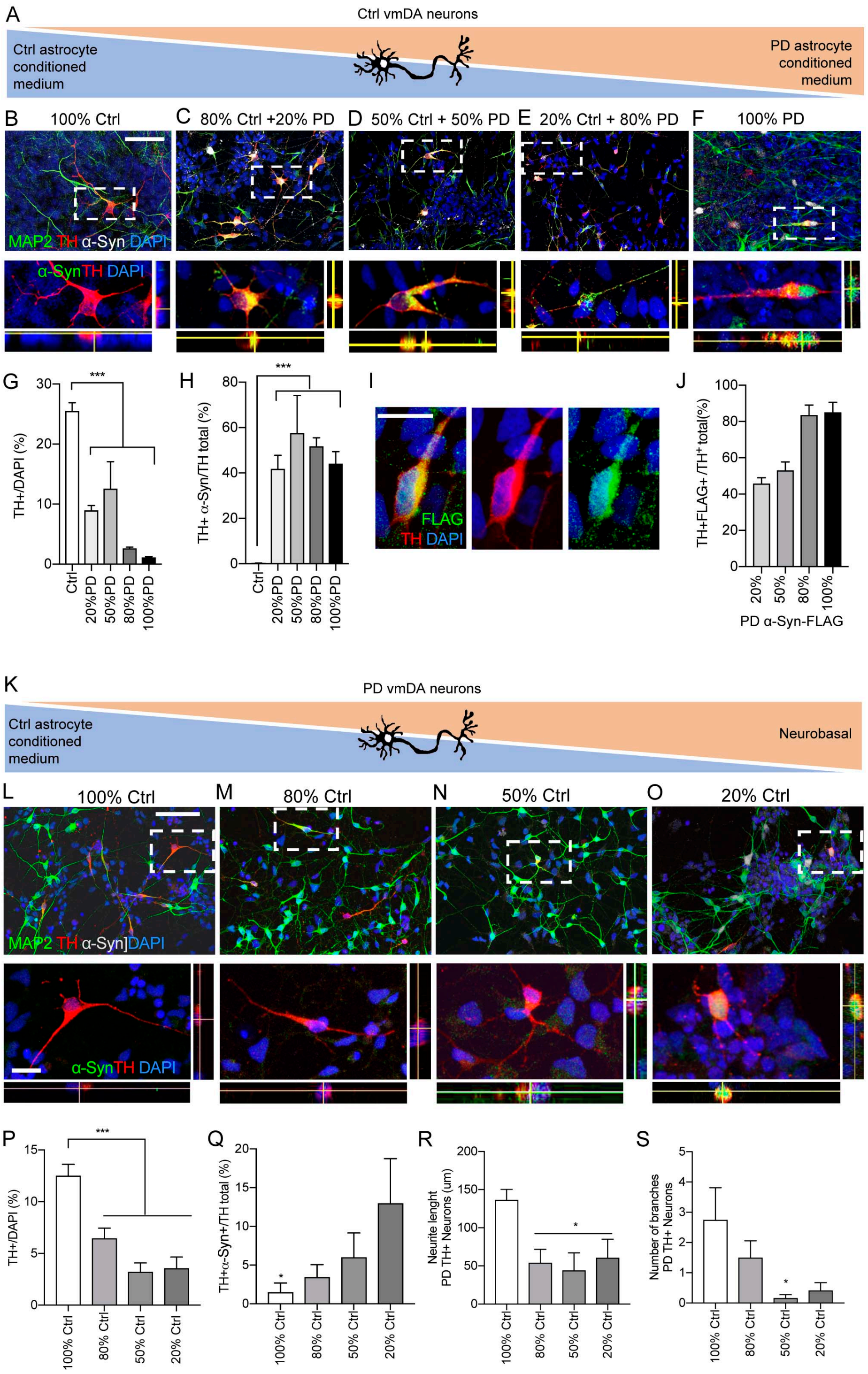

Figure S3. Effects of Ctrl and PD astrocyte conditioned medium on α-syn accumulation in Ctrl and PD vmDA neurons.

(A) Diagram representing experimental procedure for astrocyte conditioned medium (indirect contact co-culture) assay.

(B-F) Representative images showing morphology and the α-syn accumulation of Ctrl SP11 vmDAn treated with Ctrl or PD astrocytes-conditioned medium mixed in different proportions for 7 days: 100% Ctrl astrocyte medium (B) 80%Ctrl+20%PD (C), 50%Ctrl+50%PD (D), 20%Ctrl+80%PD (E) and 100%

PD (F) of Ctrl SP09 and PD SP12, respectively. Samples were stained for TH, MAP2, α-syn and DAPI. Scale bar 50μm for top picture and 20μm for all insets.

(G-H) Quantification of vmDAn over all cells (G), and those vmDAn accumulating α-syn (H) in Ctrl neurons treated with PD astrocyte-conditioned medium (n=3).

(I) Representative image showing a FLAG positive Ctrl SP11 neuron cultured with astrocyte-conditioned medium from PD SP12 α-syn -FLAG. Scale bar 20μm.

(J) Quantification of the percentage of Ctrl vmDAn stained positive for FLAG after being treated with astrocyte-conditioned medium from PD SP12 α-syn-FLAG (n=3).

All graphs plot mean ± s.e.m, unpaired two-tailed Student's t-test ***p<0.001.

(K) Diagram representing experimental procedure for astrocyte conditioned medium (indirect contact co-culture) assay.

(L-O) Representative images showing morphology and the α-syn accumulation of PD SP12 vmDAn treated with Ctrl astrocyte-conditioned medium mixed with basic neurobasal (NB) medium at different proportions for 7 days: 100% of Ctrl astrocyte-conditioned medium (L) 80%Ctrl+20%NB (M), 50%Ctrl+50%NB (N), 20%Ctrl+80%NB (O). Samples stained for TH, MAP2, α-syn and DAPI. Scale bar 50μm for top picture and 20μm for all insets.

(P-Q) Quantification of vmDAn over all cells (P), and those vmDAn accumulating α-syn (Q) in PD neurons treated with Ctrl astrocyte-conditioned medium (n=3).

(R-S) Quantification of neurite length (R) and number of branches (S) of PD vmDAn cultured with different concentrations of Ctrl astrocyte-conditioned medium (n=3); total neurons counted per experiment n=10.

All graphs plot mean ± s.e.m, unpaired two-tailed Student's t-test *p<0.05 and ***p<0.001.

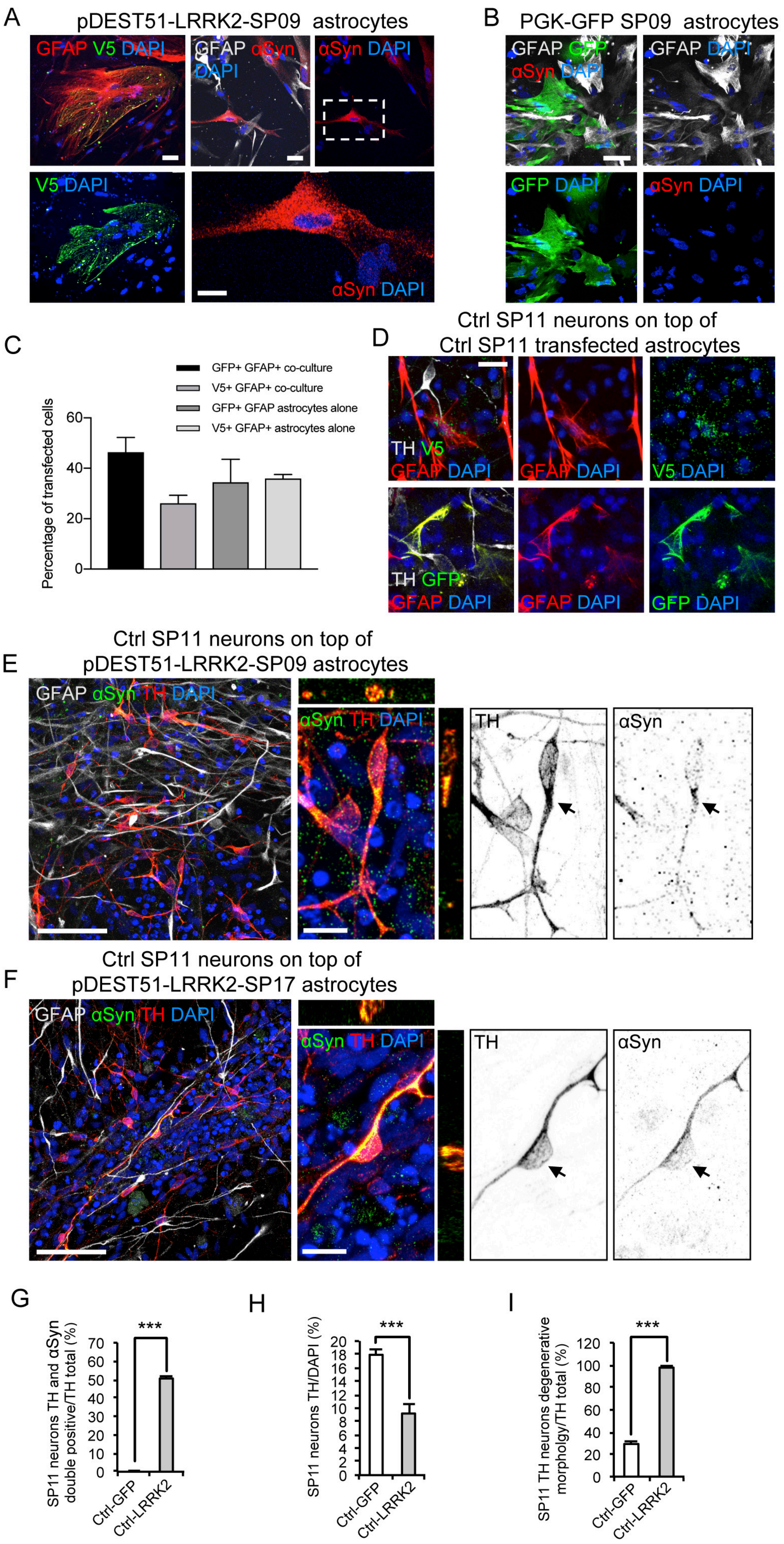

A
pDEST51-LRRK2-SP09 astrocytes
GFAP V5 DAPI
GFAP αSyn DAPI
αSyn DAPI
V5 DAPI
αSyn DAPI
B
PGK-GFP SP09 astrocytes
GFAP GFP αSyn DAPI
GFAP DAPI
GFP DAPI
αSyn DAPI
C
GFP+ GFAP+ co-culture
V5+ GFAP+ co-culture
GFP+ GFAP astrocytes alone
V5+ GFAP+ astrocytes alone
Percentage of transfected cells
60
40
20
0
D
Ctrl SP11 neurons on top of
Ctrl SP11 transfected astrocytes
TH V5 GFAP DAPI
GFAP DAPI
V5 DAPI
TH GFP GFAP DAPI
GFAP DAPI
GFP DAPI
E
Ctrl SP11 neurons on top of
pDEST51-LRRK2-SP09 astrocytes
GFAP αSyn TH DAPI
αSyn TH DAPI
TH
αSyn
F
Ctrl SP11 neurons on top of
pDEST51-LRRK2-SP17 astrocytes
GFAP αSyn TH DAPI
αSyn TH DAPI
TH
αSyn
G
SP11 neurons TH and αSyn double positive/TH total (%)
70
60
50
40
30
20
10
0
***
Ctrl-GFP
Ctrl-LRRK2
H
SP11 neurons TH/DAPI (%)
20
18
16
14
12
10
8
6
4
2
0
***
Ctrl-GFP
Ctrl-LRRK2
I
SP11 TH neurons degenerative morpholgy/TH total (%)
120
100
80
60
40
20
0
***
Ctrl-GFP
Ctrl-LRRK2

Figure S4. **Effect of p*LRRK2* G2019S transfected in control astrocytes.**

(A) Representative image of pDEST51-*LRRK2* G2019S-transfected Ctrl SP09 astrocytes stained positive for V5 epitope and showing α-syn accumulation after 14 days. Samples were stained for GFAP, V5 (control of transfection) and DAPI; or GFAP, α-syn and DAPI. Scale bar 50µm and 20µm for inset.

(B) Representative image of a Ctrl SP09 astrocyte stained positive for GFP and negative for α-syn 14 days after transfection with a GFP expression plasmid. Samples were stained for GFAP, GFP (control of transfection), α-syn and DAPI. Scale bar 20µm.

(C) Percentage of *LRRK2* G2019S (V5)- and control (GFP)-transfected astrocytes (Ctrl SP09 and Ctrl SP17) after 4 weeks in co-culture with Ctrl SP11 vmDAn or cultured alone for 2 weeks (n=3).

(D) Representative image of Ctrl SP11 vmDAn co-cultured with Ctrl SP11 GFP- or LRRK2- transfected astrocytes for 4 weeks. Samples were stained for TH, GFAP, V5 and DAPI or TH, GFAP, GFP and DAPI. Scale bar 20µm.

(E-F) Representative image of Ctrl SP11 vmDAn co-cultured with Ctrl SP09 (E) and Ctrl SP17 (F) astrocytes overexpressing *LRRK2* G2019S for 4-weeks. Samples stained for TH, GFAP, α-syn and DAPI. Scale bar, 50µm for large picture and 20µm for all zooms.

(G-I) Quantification of the percentage of Ctrl vmDAn showing α-syn accumulation (G) quantification of the percentage of survival vmDAn (H) and quantification of percentage of vmDAn showing degenerative morphology (I) after 4-week co-culture with GFP- or *LRRK2* G2019S- transfected Ctrl astrocytes (n=3). All graphs plot mean ± s.e.m, unpaired two-tailed Student's t-test, *$p<0.05$, **$p<0.01$, ***$p<0.001$.

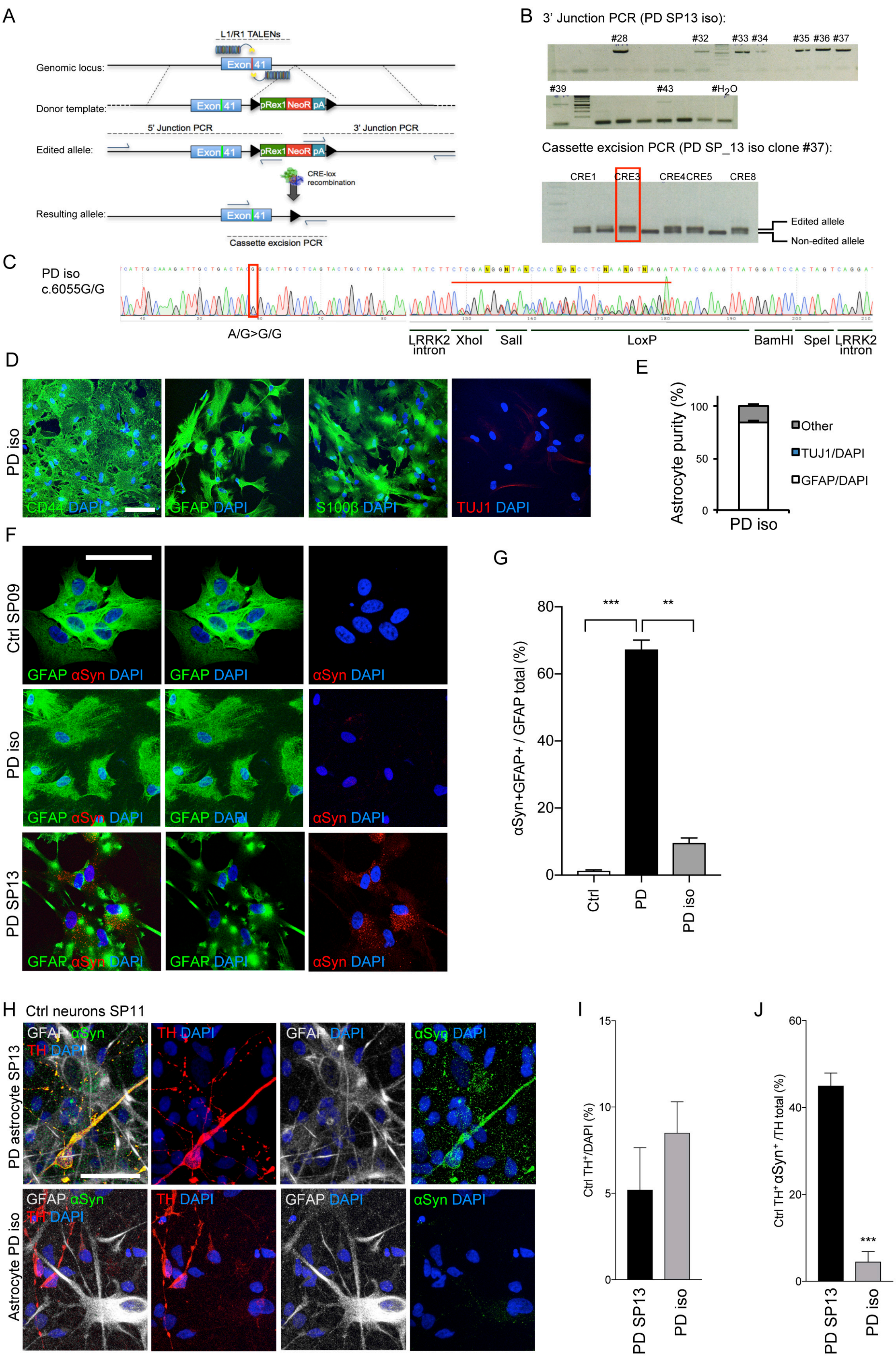
A
L1/R1 TALENs
Genomic locus:
Exon 41
Donor template:
pRex1 NeoR pA
5' Junction PCR
3' Junction PCR
Edited allele:
CRE-lox recombination
Resulting allele:
Cassette excision PCR
B
3' Junction PCR (PD SP13 iso):
#28 #32 #33 #34 #35 #36 #37
#39 #43 #H2O
Cassette excision PCR (PD SP_13 iso clone #37):
CRE1 CRE3 CRE4 CRE5 CRE8
Edited allele
Non-edited allele
C
PD iso
c.6055G/G
A/G>G/G
LRRK2 intron
XhoI
SalI
LoxP
BamHI
SpeI
LRRK2 intron
D
PD iso
CD44 DAPI
GFAP DAPI
S100β DAPI
TUJ1 DAPI
E
Astrocyte purity (%)
100
50
0
Other
TUJ1/DAPI
GFAP/DAPI
PD iso
F
Ctrl SP09
PD iso
PD SP13
GFAP αSyn DAPI
GFAP DAPI
αSyn DAPI
G
***
**
αSyn+GFAP+ / GFAP total (%)
80
60
40
20
0
Ctrl
PD
PD iso
H
Ctrl neurons SP11
PD astrocyte SP13
Astrocyte PD iso
GFAP αSyn TH DAPI
TH DAPI
GFAP DAPI
αSyn DAPI
I
Ctrl TH+/DAPI (%)
15
10
5
0
PD SP13
PD iso
J
Ctrl TH+ αSyn+ /TH total (%)
60
40
20
0
***
PD SP13
PD iso

Figure S5. Schematic description of the generation of isogenic control line and phenotypic comparison between isogenic clones, alone or in co-culture with Ctrl-SP11 neurons. (A) Scheme describing the gene editing process resulting in the generation of isogenic *LRRK2* G2019S iPSC line using TALENs. Blue arrows represent the primers used for the PCR screening procedure. Black triangles represent LoxP sites surrounding the selection cassette. Green and red bars in Exon 41 represent the *wild type* and the G2019S alleles respectively.

(B) Molecular analysis of the resistant clones checking proper pRex1-NeoR cassette integration and subsequent cassette excision in PD SP13 iPSC line. In the lower gel, the increase in size of the edited clone is due to the remaining LoxP site. Red rectangles indicate selected #37CRE3 clone.

(C) Sanger sequencing, confirmed both successful excision of the LoxP site-flanked cassette and the successful correction of the mutation.

(D) Representative images of PD iso astrocytes staining positive for CD44, GFAP, S100β and negative for TUJ1. Scale bar 100 μm.

(E) Astrocyte cultures of PD iso line are approximately composed of 84% astrocytes, 1% neurons and 15% other (n=3).

(F) Representative images of Ctrl SP09, PD iso and PD SP13 astrocytes at 14 days stained for GFAP and α-syn. Scale bar 50 μm.

(G) Percentage of PD SP13 and PD iso astrocytes positive for α-syn after 14 days in culture (n=3).

(H) Representative images of Ctrl SP11 vmDAn on top of PD SP13 (upper panel) and PD iso astrocytes (bottom panel). Samples were stained for TH, GFAP, α-syn and DAPI. Scale bar 20 μm.

(I-J) Quantification of the percentage of Ctrl vmDAn remaining after 4-week co-culture with PD SP13 or PD iso astrocytes (I). Percentage of those remaining vmDAn showing α-syn accumulation (J) (n=3).

All graphs plot mean ± s.e.m, unpaired two-tailed Student's t-test, *p<0.05, **p<0.01, ***p<0.001.

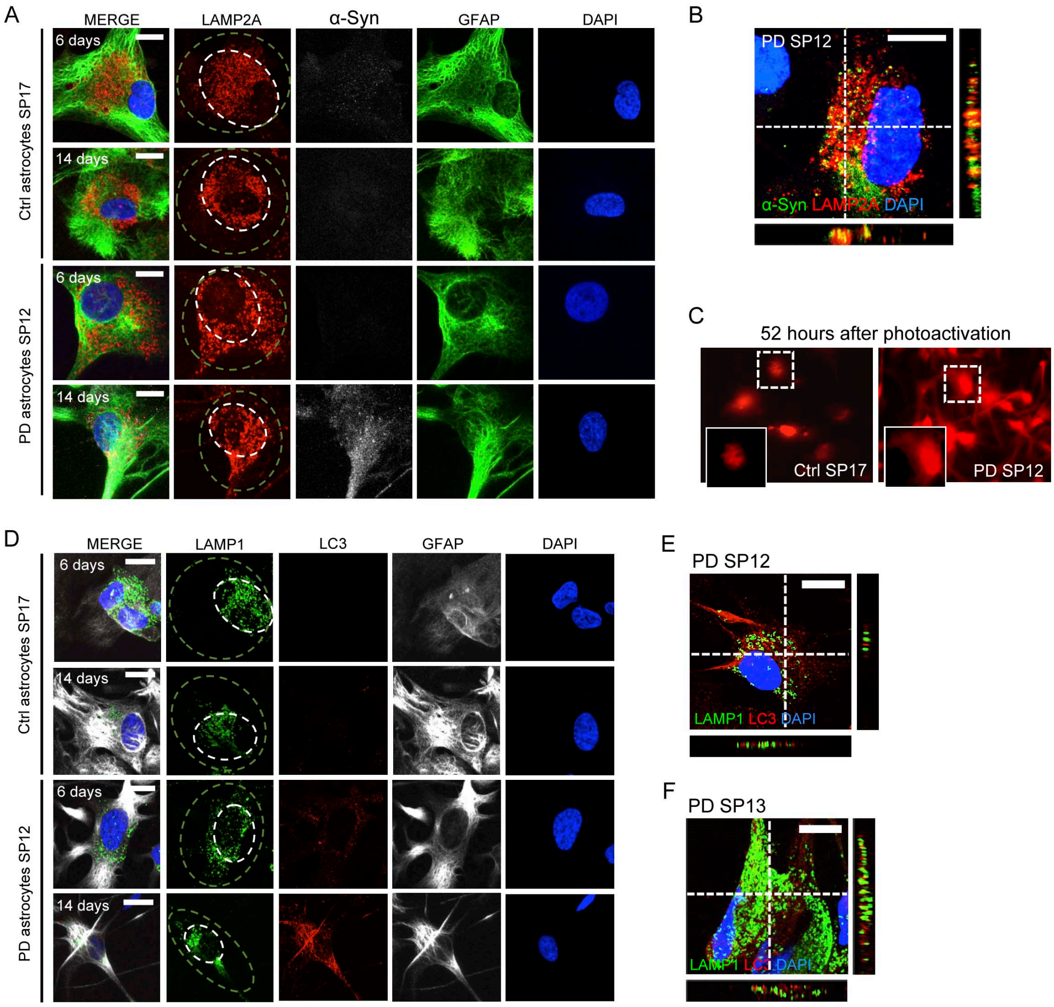

Figure S6. Altered CMA, dysfunctional macroautophagy and α-syn accumulation in PD astrocytes. (A) Representative images of Ctrl SP17 and PD SP12 astrocytes stained positive for the LAMP2A, GFAP, α-syn and DAPI after 6 and 14 days in culture. Scale bar 20µm. Smaller white circles represent perinuclear area, whereas larger green circles represent non-perinuclear area.

(B) Positive co-localization between LAMP2A and α-syn in PD SP12 astrocyte line. Scale bar 10µm.

(C) KFERQ-DENDRA (CMA reporter) in Ctrl SP17 and PD SP12 astrocytes 52 hours after photo-switching with UV light (n=3).

(D) Representative images of lysosomal protein marker LAMP1 and autophagosome marker LC3 in Ctrl SP17 and PD SP12 astrocytes (GFAP) at 6 and 14 days. Scale bar 20µm. Smaller white circles represent perinuclear area, whereas larger green circles represent non-perinuclear area (n=3).

(E-F) Lack of co-localization between lysosomes (LAMP1) and autophagosomes (LC3) in astrocyte lines PD SP12 and PD SP13. Scale bar 10µm.

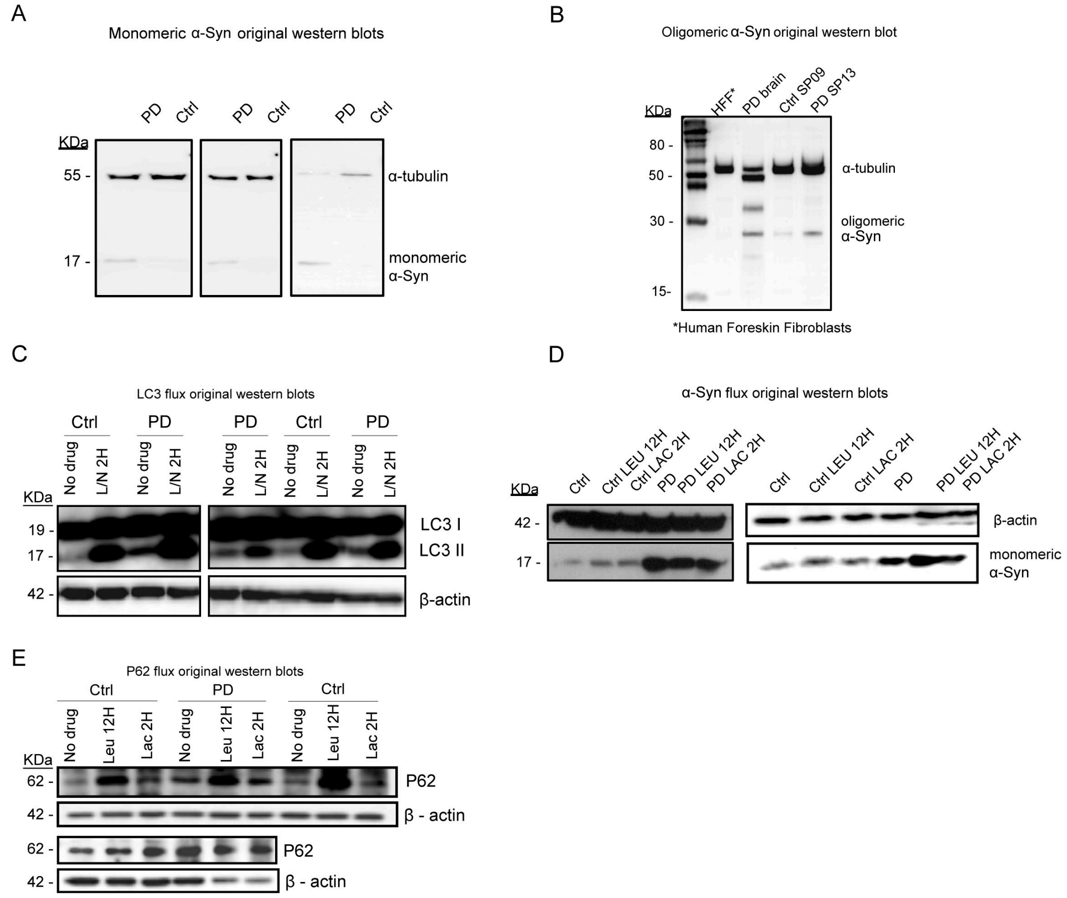

Figure S7. Original uncropped Western blots.

(A) Monomeric α-syn protein Western blots.

(B) Oligomeric α-syn protein Western blot.

(C) LC3 flux Western blots.

(D) α-Syn flux Western blots.

(E) p62 flux Western blots.

SUPPLEMENTAL EXPERIMENTAL PROCEDURES

iPSC-derived astrocyte generation and culture

The parental iPSC lines used in our studies were previously generated and fully characterized (Sanchez-Danes et al., 2012). Specifically, we used iPSC generated from three patients harboring the G2019S mutation on the *LRRK2* gene (PD SP06, PD SP12, and PD SP13), and from three healthy age-matched controls (Ctrl SP09, Ctrl SP11, and Ctrl SP17). The generation of isogenic control and α-syn-FLAG-tagged iPSC lines is described below. The generation and/or use of human iPSCs in this work were approved by the Spanish competent authorities (Commission on Guarantees concerning the Donation and Use of Human Tissues and Cells of the Carlos III National Institute of Health). All procedures were done in accordance with institutional guidelines and the human iPSC lines have been (or are in the process of being) deposited at the Spanish National Stem Cell Bank, according to the Spanish legislation. iPSC were differentiated into spherical neurospheres containing neuroectodermal progenitors and then differentiated toward an astrocytic lineage following a previously published protocol (Serio et al., 2013). First, the SNMs were grown in suspension for 28 days with Induction Medium (DMEM/F12, 1% N2 supplement, 0.1% B27 supplement (Life, 17504-044), 1% nonessential amino acids (NEAA), 1% penicillin/streptomycin (PenStrep), 1% Glutamax) supplemented with 20 ng/mL LIF (Sigma) and 20 ng/mL EGF (R&D Systems), and then for further 21 days with Propagation Medium (DMEM/F12, 1% N2 supplement, 0.1% B27 supplement, 1% NEAA, 1% PenStrep, 1% Glutamax) containing 20 ng/mL FGF-2 (PeproTech) and 20 ng/mL EGF (R&D Systems). Finally, SNMs were incubated with accutase (LabClinics) for 15 minutes at 37ºC, mechanically dissaggregated and plated on matrigel-coated plates as a monolayer. The monolayer of neural progenitors was cultured for 14 more days in Propagation Medium and then for 14 more days in CNTF medium (Neurobasal, 1% Glutamax, 1% PenStrep, 1% NEAA, 0.2% B27 supplement, 10 ng/mL CNTF (Prospec Cyt-272), a stage in which they were considered astrocyte progenitors and therefore characterized. These astrocyte progenitors were successfully frozen in Astrocyte Freezing Medium (90% FBS and 10% DMSO) and stored in liquid nitrogen for future use. When needed for an experiment, vials were thawed in medium containing FBS, resuspended in CNTF medium and plated on matrigel-coated plates. Cells were passaged four times before considered mature and then further characterized. Experiments were performed with astrocytes growing on Thermanox™ plastic coverslips (Thermofisher) coated with matrigel in 24-well plates.

iPSC-derived vmDAn generation

Four different iPSC, two PD (SP12 and SP13) and two Ctrl (SP11 and SP11#4) were differentiated into dopaminergic neurons using a combination of two previously published protocols for midbrain induction (Chambers et al., 2009; Kriks et al., 2011). Briefly, iPSC were maintained in conditioned HES medium for 5 days until they reached 70% of confluence, and were then cultured in SRM medium (day 0) (KO-DMEM, 15% KO serum, 1% NEAA, 1% GlutaMax and 1% P/S) for additional 5 days. After that, iPSCs were grown in Neurobasal medium, 1% N2, 2% B27 without vitamin A and 1% P/S. At day 12, N2 was removed from the medium until the end of the differentiation. Media were supplemented with SB-431542 (10 mM; day 0–day 5; Sigma), LDN193189 (100 nM; day 0–day 12; Miltenyi), CHIR99021 (3 mM; day 3–day 25; Miltenyi), purmorphamine (2 mM; day 1–day 5; Stemgent, Cambridge, Massachusetts,

www.stemgent.com), Smoothened Agonist (SAG) (1 mM; day 1–day 5; Tocris, Bristol, United Kingdom, www.tocris.com), Brain-Derived Neurotropic Factor (BDNF) (20 ng/ml; from day 12; Miltenyi), Glial Cell-Derived Neurotropic Factor (GDNF) (20 ng/ml; from day 12; Miltenyi), DAPT (10 mM; from day 12; Tocris), db-cAMP (500 mM; from day 12; Sigma, St. Louis, Missouri, www.sigmaaldrich.com), TGFB3 (1 ng/ml; from day 12; Miltenyi) and ascorbic acid (AA, 200 mM; from day 12; Sigma). On day twenty, cells were dissociated using Accutase (Merck, Kenilworth, New Jersey, www.merck.com), re-plated at 0,75 x 10^5 cells per cm^2 on dishes pre-coated with polyornithine (15 μg/ml), laminin (1 μg/ml) and fibronectin (2 μg/ml) and cultured in Neurobasal medium with 2% B27 without vitamin A and 1% P/S with trophic factors (GDNF, BDNF, TGFB3, AA, cAMP and DAPT) until analysis. Quantification of neurons (at day 35 and 50) was assessed by confocal microscopy using anti-FOXA2, anti-MAP2, anti-TH, anti-GIRK2 and anti-DAT antibodies. After 35 days of differentiation 20% of overall cells stained positive for TH, a number which increased with time reaching 35% after 50 days, a point in which the neurons were considered as mature.

Gene Editing

TALEN, CRISPR guideRNA (gRNA) and Donor Plasmid Design. TALEN monomers were engineered as described elsewhere (Mussolino et al., 2011) in the Institute for Cell and Gene Therapy & Center for Chronic Immunodeficiency (University of Freiburg). They were composed of 19 RVDs and were fused to wild type FokI nuclease domains. Repeats containing the NN RVD were used for Guanidine recognition. Each monomer was inserted in a plasmid under the control of a modified CMV promoter (Alwin et al., 2005). Different TALEN monomers recognizing DNA motifs adjacent to the genomic G2019S mutation site were designed. The TALEN combination showing the highest cleavage efficiency in the T7EI assay was chosen for gene correction. CRISPR/Cas9 gRNAs targeting the last exon-3'UTR junction of the human *SNCA* gene were designed so that the spacer sequence overlapped the STOP codon. Complementary oligos encoding for the desired spacer sequences were annealed and ligated into the BbsI site of the Cas9/guideRNA co-expression plasmid px458 (Addgene #48138). In order to increase Cas9 expression in hPSC, CBh promoter was replaced by the full-length CAGGS promoter (pCAGGS-458). The gRNA with the highest cleavage efficiency in the T7EI assay was selected for the editing procedure. Donor templates for HDR were generated using standard molecular cloning procedures. Briefly, for *LRRK2* donor template, homology arms (HAs) spanning approximately 800 bp from a position 34 bp downstream *LRRK2* exon 42 were amplified from genomic DNA from either wild-type or *LRRK2* G2019S mutant hiPSC. A floxed selection cassette was placed in between the HAs. The selection cassette, pRex1-NeoR-SV40pA, was amplified from aMHC-eGFP-Rex-Neo (Kita-Matsuo et al., 2009) (Addgene; #21229) with primers containing the LoxP sites in the proper orientation.

Donor plasmid for knocking-in a FLAG tag fused C-terminal to the α-syn open reading frame (ORF) was engineered using the following elements. Two homology arms (HAs) spanning approximately 800 bp from both sides of the STOP codon. The sequence encoding for the FLAG-tag was placed right after the last codon of the *SNCA* ORF and before the STOP codon. A selection cassette (pRex1-NeoR) flanked by loxP sites was cloned between the STOP codon and the 3'HA.

The primers employed during the cloning procedure were: SNCA_gRNA2_OL-F 5’-TGGGAGCAAAGATATTTCTT-3’, SNCA_gRNA2_OL-R 5’- AAGAAATATCTTTGCTCCCA-3’ for cloning the guideRNA spacer sequence into the pCAGGS-458 plasmid; LRRK2_5-F_KpnI 5’- AAG GTA CCC CTT AAT ATC TAA CAT GAT TAG G-3’, LRRK2_5-R_XhoI 5’- AAC TCG AGA AGA TAG AAT TAT GAG ACA GAC-3’, Rex1_F-loxP-SalI 5’- AAG TCG ACA TAA CTT CGT ATA GCA TAC ATT ATA CGA AGT TAT GAC CGA TTC CTC CCG ATA AG-3’, Neo_R-loxP-BamHI 5’- AA GGATCC ATAACTTCGTATA ATGTATGC TATACGAAGTTAT TAAGATACATTGATGAGTTTGGA-3’, LRRK2_3-F_SpeI 5’- AAA CTA GTC AGG ATG GAT AAC CAC TGA C-3’, LRRK2_3-R_NotI 5’- AAG CGG CCG CTC CCT AAA GAT AGA GTG TTC C-3’ For LRRK G2019S correction;

and SNCA_5-F_XhoI 5’- AACTCGAGACTCAAGCTTAGGAACAAGGA-3’, SNCA_5FLAG-R_SalI 5’-AAGTCGACATAACTTCGTATAGCATACATTATACGAAGTTATGACCGATTCCTCCCGATAAG-3’, Rex1_F-loxP-SalI 5’-AAGTCGACATAACTTCGTATAGCATACATTATACGAAGTTATGACCGATTCCTCCCGATAAG-3’, Neo_R-loxP-BamHI 5’-AAGGATCCATAACTTCGTATAGCATACATTATACGAAGTTATTAAGATACATTGATGAGTTTGGA-3’, SNCA_3-F_BamHI 5’-AAGGATCCGAAATATCTTTGCTCCCAGT-3’, SNCA_3-R_NotI 5’-AAGCGGCCGCTTAAGGAACCAGTGCATAC-3’ for SNCA-FLAG donor plasmid construction.

CRISPR-mediated *SNCA* locus edition in hiPSC. The day before transfection, 800.000 Ctrl SP11 and PD SP12 iPSC lines were seeded in a 10cm plate coated with matrigel. The following day, hiPSC were co-transfected with a mix of 6 µg of Cas9-T2A-EGFP/gRNA, 9 µg of the donor plasmid, 45 µL of FuGENE HD (Promega) transfection reagent and KO-DMEM up to 750 µL. The transfection mixture was incubated for 15 minutes at RT and subsequently added to the cells dropwise. 50 µg/mL Geneticin (G-418; Melford Labs) selection was initiated 72h post-transfection and was maintained until the emerging colonies were transferred to another plate. Between 10 and 14 days after the initiation of the selection, colonies were large enough as to be screened. Half of the colony was sampled in order to check site-specific integration by means of PCR. Those colonies that were positive for the targeted recombination were transferred to a different well in order to be transfected with a CRE-recombinase expression plasmid. After CRE transfection, cells were singularized and seeded at a low density on top of an irradiated human fibroblast feeder layer in the presence of ROCK inhibitor (Miltenyi). Once, the colonies attained a certain size, they were isolated and screened for the excision of the selection cassette. Those clones whose both *SNCA* alleles were tagged with the FLAG epitope were expanded and characterized in terms of pluripotency and genome stability. The primers used for the screening procedure and the molecular characterization; SNCA_5Out_F (referred as 5’Out in **Fig. S4**) 5’-CTCACACAGACACACGAAAGG-3’, FLAG_R 5’-AGCACCGAAATGCTGAGTG-3’, Check_Rex1Neo_3HA_F 5’-CCCGTCTGTTGTGTGACTC-3’, SNCA_3Out_R (referred as 3’Out in **Fig. S4**) 5’-ACGTAAAGCAAACATTGACAGG-3’, SNCA_T7_F (referred as 5’In in **Fig. S4**) 5’-TGCATCCGGATCAGAACCTA-3’, SNCA_T7_R (referred as 3’In in **Fig. S4**) 5’-AGCACCGAAATGCTGAGTG-3’.

TALEN-mediated LRRK2 G2019S correction in hiPSC. The day of transfection, cells were detached from a confluent 10-cm plate and were electroporated as small clumps with 15 ug of each TALEN monomer and 30 ug of a plasmid donor template. This plasmid contained two arms of homology (the left one bearing the wild type allele) placed at both sides of a floxed pRex1-NeoR-SV40pA selection cassette. The primers used for the screening procedure and the molecular characterization; LRRK2_Out_5'HA_F 5'- TCGTGATTGCGTGGGTC-3', LRRK2_Out_3'HA 5'-GCAGGAAACGAACTAGAACC -3', Check_Rex1Neo_5HA_R 5'-CTTATCGGGAGGAATCGGTC-3', Check_Rex1Neo_3HA_F 5'-CCCGTCTGTTGTGTGACTC-3', LRRK2_T7_F 5'-GGGACAAAGTGAGCACAG-3', LRRK2_T7_Sel_R 5'- CACAAGTGCCAACAATACC-3'.

Astrocytes and vmDA co-culture

$2x10^4$ astrocytes were plated per well in 24-well plates pre-coated with matrigel and let to generate a confluent monolayer. One week after, vmDAn (35-day old) were plated onto the astrocyte monolayer at $5x10^4$ cells per well. Co-cultures were carried out in Neurobasal medium, containing 1% PenStrep, 2% B27 supplement minus Vitamin A (ThermoFisher, 12587001). In addition, we tested the effect of the vmDAn medium in astrocytes alone without noticing any differences in astrocyte survival (data not shown). Cells were fixed and stained for the markers indicated in each experiment. For assessing vmDA neuron survival, the abundance of TH+ cells was counted using FIJI is Just ImageJ™ cell counter plugin.

Astrocyte conditioned medium (indirect co-culture)

$3x10^5$ astrocytes were plated per well on a matrigel coated 6-well plate in 2mL of CNTF medium. Each line was cultured for 14 days without changing the initial medium. At day 6, 1mL of fresh CNTF medium was added to each well. After the 14-day time-point, the medium was collected and frozen at -80ºC. Different ratio of Ctrl SP09 and PD SP12 astrocyte-conditioned medium was mixed (Ctrl and PD%: 100, 80/20, 50/50, 20/80, 100) to obtain medium to treat Ctrl SP11 neurons. Using the same paradigm, Ctrl SP11 neurons were treated with different ratio of Ctrl SP09 and PD SP12 α-syn-FLAG mixed medium (100, 80/20, 50/50, 20/80) to verify the effective transfer (contact independent) of the α-syn from the astrocyte conditioned medium to neurons. In contrast, to treat PD SP12 neurons, we only mixed Ctrl SP09 astrocyte-conditioned medium with Neurobasal Medium (% respectively, 100, 80/20, 50/50, 20/80).

Immunocytochemistry

Samples were fixed using 4% PFA for 15 minutes and then washed three times for 15 minutes with PBS. Samples were blocked and permeabilized with TBS++ with low triton (TBS, 3% Normal Donkey Serum, 0.01% Triton X-100) for 2 hours and subsequently incubated with the primary antibody for 48 hours at 4ºC. Primary antibodies used include mouse anti- CD44 (Abcam, ab6124), guinea pig anti-GFAP (Synaptic Systems, 173 004), rabbit anti-GFAP (Dako, Z0334), rabbit anti-S100β (Dako, 311), mouse anti-Vimentin IgM (Iowa, 3CB2), mouse anti-TUJ1 (Covance, MMS-435P), rabbit anti-MAP2 (Sant Cruz, sc-20172), rabbit anti-NG2 (Millipore, AB5320), guinea pig anti-GLT-1 (Millipore, AB1783), rabbit anti-Synapsin-I (Calbiochem, 574777), rabbit anti-LC3B (Cell Signaling, 2775), rabbit anti-LAMP-2A (Abcam, 18528), mouse anti-FLAG-M2 (Sigma, F3165), mouse α-syn (BD, 610787), mouse anti-α-syn (Agisera AS13 2718), sheep anti-TH (Pel-Freez, P60101-0), rabbit anti-TH (Santa Cruz, sc-

14007), mouse anti-LAMP1 (Iowa, H4A3). Samples were then washed with TBS 1x for 15 minutes three times, and blocked again for one hour at RT. Samples were incubated with secondary antibodies (1:200) for 2 hours at RT: Alexa Fluor 488 anti-Mouse IgG (Jackson 715-545-150), Cy3 anti-rabbit IgG (Jackson 711-165-152), DyLight 649 anti-Guinea pig IgG (Jackson 706-495-148), Alexa Fluor 647 anti-Sheep (Jackson 713-605-147), Cy™2 AffiniPure Donkey Anti-Rabbit IgG (H+L) (Jackson 711-225-152), Cy™3 AffiniPure Donkey Anti-Mouse IgG (H+L) (Jackson 715-165-151). Samples were then washed with TBS 1x for 15 minutes three times, incubated with nuclear staining DAPI (Invitrogen, 1:5000) for 10 minutes, mounted with PVA:DABCO and stored at 4ºC until imaged. Samples were imaged using an SP5 confocal microscope (Leica) and analyzed with FIJI is Just ImageJ™.

RNA extraction and gene expression analysis

The isolation of total mRNA was performed with the RNeasy Micro Kit and treated with RNase free DNase I (Qiagen). 500ng were used to synthesize cDNA with the SuperScript III Reverse Transcriptase Synthesis Kit (Invitrogen). Quantitative RT-PCR analyzes were done in triplicate using 2ng/ul cDNA with Platinum SYBR Green qPCR Super Mix (Invitrogen) in an ABI Prism 7000 thermocycler (Applied Biosystems). All results were normalized to β-actin.

Stranded mRNA library preparation and sequencing

Total RNA was assayed for quantity and quality using Qubit® RNA HS Assay (Life Technologies) and RNA 6000 Nano Assay on a Bioanalyzer 2100. The RNASeq libraries were prepared from total RNA using the TruSeq®Stranded mRNA LT Sample Prep Kit (Illumina Inc., Rev.E, October 2013). Briefly, 500ng of total RNA was used as the input material and was enriched for the mRNA fraction using oligo-dT magnetic beads. The mRNA was fragmented in the presence of divalent metal cations and at high temperature (resulting RNA fragment size was 80-250 nt, with the major peak at 130nt). The second strand cDNA synthesis was performed in the presence of dUTP instead of dTTP, this allowed to achieve the strand specificity. The blunt-ended double stranded cDNA was 3´adenylated and Illumina indexed adapters were ligated. The ligation product was enriched with 15 PCR cycles and the final library was validated on an Agilent 2100 Bioanalyzer with the DNA 7500 assay. The libraries were sequenced on HiSeq2000 (Illumina, Inc) in paired-end mode with a read length of 2x76 bp using TruSeq SBS Kit v4. We generated over 30 million paired-end reads for each sample in a fraction of a sequencing v4 flow cell lane, following the manufacturer's protocol. Image analysis, base calling and quality scoring of the run were processed using the manufacturer's software Real Time Analysis (RTA 1.18.66.3) and followed by generation of FASTQ sequence files by CASAVA. The RNA-seq data have been deposited in Gene Expression Omnibus (GEO) of the National Center for Biotechnology Information and are accessible through GEO Series accession number GSE116124 (www.ncbi.nlm.nih.gov/geo/query/acc.cgi?acc=GSE116124).

Bioinformatics of RNA sequencing

RNA-seq paired-end reads were mapped against the human reference genome (GRCh38_primary) using STAR version 2.5.3a (Dobin et al., 2013) with ENCODE parameters for long RNA. Annotated gene and

isoforms (gencode version 27) were quantified using RSEM version 1.3.0 with default parameters (Li and Dewey, 2011). Differential expression analysis was performed with DESeq2 version 1.10.1 (Love et al., 2014). Heatmaps were performed with the 'pheatmap' R package with Euclidean distances comparing hiPSC-astrocytes (Ctrl SP09 and PD SP12) to human cortical astrocytes and iPSC lines (Ctrl SP09 and PD SP12).

ATP production assay

ATP production was measured using the ATP Determination Kit (A22066, Molecular Probes), using a recombinant firefly luciferase and its substrate D-Luciferin. Each astrocyte line was tested by plating $2x10^4$ cells per well of a 24-well plate. Cells were washed twice with ice-cold PBS. The cells were scraped with 100 µL of ATP buffer (100 nM Tris-HCl pH 7.75, 4 nmM EDTA), collected and flash frozen in liquid nitrogen, boilt for 3 minutes and kept on ice for 5 min. Samples were then centrifuged at 4ºC for 5 min at 13,000 rpm. The ATP content in the supernatant was measured with the ATP determination kit. Each reaction contained 1.25 µg/mL of firefly luciferase, 50 µM D-luciferin and 1 mM DTT in 1X Reaction Buffer. After a 15-min incubation, luminescence was measured (arbitrary units).

Calcium imaging

$2x10^4$ astrocytes were plated per well in 24-well plates. Live astrocytes at passage 4 were incubated with Fluro4-AM flurofore for 30 minutes slowly shaking at RT. Astrocytes were then imaged during 20 minutes using the Hokawo program. Recorded data was converted from video to images. The data was loaded into NeuroImage software where Calcium Activity Map and individual Calcium Graphs were generated. Data is further analyzed in a Matlab code made by Dr. Jordi Soriano laboratory.

Cell viability assay

Calcein Green AM (Thermofisher C3100MP) (1µM) and Propidium iodide (Sigma P4170) (1 mg/mL) were added to cells resuspended in 0.5 mL Ca^{2+}/Mg^{2+}-free PBS supplemented with 2%FBS. Flow cytometry analysis was performed on a Gallios flow cytometer using a 488nm laser for excitation and 525/40 nm and at 575/30 nm emission filters for recording Calcein Green AM and Propidium Iodide signal respectively. Interpretation of cytometry data was done using Kaluza Software (Beckman Coulter Inc, Brea, CA).

CMA Activator (CA) treatment

We used a CMA activator (CA) that operates through the release of the endogenous inhibition of the retinoic receptor-α signaling pathway over CMA (Anguiano et al., 2013). Astrocytes (PD SP13) were treated with 20µM CA for 4 days (conditions inducing maximal activity and no toxicity of CA as evaluated in preliminary dose-response analyses using mouse fibroblasts), then fixed and analyzed for α-syn accumulation. Co-cultures of Ctrl SP11 neurons on PD astrocytes (SP13 and SP12) started CA treatment from the 2nd week and it was added daily until the 4th week when cells were fixed, stained and analyzed for neuronal survival and α-syn accumulation within DA neurons and astrocytes.

Protein extraction

For pellet collection, $3x10^5$ cells per well of a matrigel-coated 6-well plate were plated and each well equated to one pellet. Live cells were washed twice with PBS and incubated for 6 minutes at 37ºC with accutase (Sigma). Cells were lifted and collected in washing medium containing FBS and centrifuged at 800rpm for 5 minutes. After centrifugation, cells were resuspended in cold PBS and centrifuged for 5 minutes at 4ºC at 600xg. Pellets were immediately stored at -80ºC for future use. For protein extraction, pellets were homogenized in 50mM Tris-HCl, pH 7.4/150 mM NaCl/0.5% Triton X-100/0.5% Nonidet P-40 and a mixture of proteinase inhibitors (Sigma, Roche tablet). Samples were then centrifuged at 15,000xg for 20 minutes at 4ºC. The resulting supernatant was normalized for protein using BCA kit (Pierce). For blotting oligomeric forms of α-syn, cells were lysed in Mila lysis buffer (0.5M Tris at pH 7.4 containing 0.5 methylenediaminetetraacetic acid at pH 8.0, 5M NaCl, 0.5% Na doxicholic, 0.5% Nonidet P-40, 1mM phenylmethylsulfonyl fluoride, bi-distilled water, protease and phosphatase inhibitor cocktails) (Roche Molecular Systems, Pleasanton, CA, USA), and then centrifuged for 15 min at 13,000rpm at 4°C (Ultracentrifuge Beckman with 70Ti rotor, CA, USA).

Western blot (WB)

Cell extracts were boiled at 100ºC for 5 minutes, followed by 7-15% SDS-PAGE, electrotransferred to PVDF membranes for 1.5 hours at 4ºC and blocked with 5% not-fat milk in 0.1M Tris-buffered saline (pH= 7.4) for 1 hour. Membranes were incubated O/N at 4ºC with primary antibodies diluted in TBS/3% BSA/0.1% TWEEN. After incubation with peroxidase-tagged secondary antibodies (1:10,000), membranes were revealed with ECL-plus chemiluminescence western blot kit (Amershan-Pharmacia Biotech). The following antibodies were used: mouse anti-α-syn (BD, 610787), rabbit anti-LAMP-2A (Abcam, 18528), rabbit anti-LC3B (Cell Signaling, 2775), rabbit anti-p62 (Enzo Life Science, BML-PW9860), mouse anti-β-actin (Millipore) and rabbit anti-α-tubulin Millipore). Films were scanned at 2,400 x 2,400 dpi (i800 MICROTEK high quality film scanner), and the densitometric analysis was performed using FIJI is Just ImageJ™. Other membranes were imaged using the ChemiTouch machine under the 'Optimal exposure' setting.

KFERQ-DENDRA CMA reporter

CMA activity was measured using a photo-switchable CMA fluorescent reporter with a CMA targeting motif fused N-terminally to PS-Dendra protein (KFERQ-PS-Dendra) (Koga et al., 2011; Park et al., 2015). $2x10^4$ astrocytes seeded in wells of 24-well plates were transduced after 6 days in culture. Three days later, cells were photo-switched with UV light for 3 minutes and then imaged after 52 hours to monitor CMA activity.

CMA knockdown (shLAMP2A)

$2x10^4$ astrocytes seeded in wells of 24-well plates were transduced after 12 days in culture with LV-shLAMP2A (Massey et al., 2008) or LV-shLuciferase as a control. Three days later, cells were fixed and stained for α-syn. The cellular area occupied by α-syn puncta was measured using a macro developed in FIJI is Just ImageJ™.

LC3 flux assay

$3x10^5$ astrocytes seeded in wells of 6-well plates were treated after 14 days in culture with 100 µM leupeptin (Sigma L2884) and 20 mM NH_4Cl (Sigma A9434) for 2 hours in order to stop lysosomal proteolysis. The pellets were collected and protein extracts were electrophoresed in 13% SDS-PAGE gels. LC3-II flux was calculated as the difference between 2-hour drug treatment versus untreated.

α-Syn flux assay

$3x10^5$ astrocytes seeded in MW6 wells were treated after 14 days in culture with either 100 µM leupeptin (Sigma L2884) for 12 hours or with the proteoseomal inhibitor lactacystin (5mM, Enzo BML-PI104) for 2 hours. The pellets were collected and protein extracts were electrophoresed in 12.5% SDS-PAGE.

***LRRK2* G2019S overexpression**

$2x10^4$ astrocytes seeded in 24-well plates were co-transfected after 7 days in culture with 1 µg pDEST51-LRRK2-G2019S, which was a gift from Mark Cookson (Addgene plasmid # 29401) and 0.25ug of GFP expression plasmid as transfection control. Transfection was done using Lipofectamine Stem Reagent (Invitrogen) following manufacturer's instructions. Cells were then used for co-culture or fixed and analyzed after one week from the transfection. Cells were stained with an anti-V5-tag or anti-GFP antibody in order to estimate the efficiency of transfection both of astrocytes growing alone or when co-cultured with neurons. Astrocytes used for co-culture had the medium changed the day after the transfection and then during the neuronal plating. Ctrl SP11 vmDA neurons at day 35 were plated on top of the transfected astrocytes and maintained in culture following the previously described protocol for 4 weeks.

Statistical analysis

Statistical analyses of the obtained data were performed using two-tailed unequal variance Student *t*-tests and ANOVA (* $p<0.05$, ** $p<0.01$, *** $p<0.001$) and the mean and standard error of the mean were plotted using Prism (Mac OS X). Number of independent experiments (n) is indicated in each figure legend.
